# The Application of Reversible Intramolecular Sulfonamide Ligation to Modulate Reactivity in Organometallic Ruthenium(II) Diamine Complexes

**DOI:** 10.3390/molecules25020244

**Published:** 2020-01-07

**Authors:** Samuel A. Kemp, Timothy J. Prior, Huguette Savoie, Ross W. Boyle, Benjamin S. Murray

**Affiliations:** Department of Chemistry and Biochemistry, University of Hull, Cottingham Road, Hull HU6 7RX, UK; samkemp1996@aol.com (S.A.K.); T.Prior@hull.ac.uk (T.J.P.); H.Savoie@hull.ac.uk (H.S.); R.W.Boyle@hull.ac.uk (R.W.B.)

**Keywords:** metallodrugs, bioorganometallic chemistry, ruthenium, cancer, sulfonamide

## Abstract

Metallation of biomacromolecular species forms the basis for the anticancer activity of many metallodrugs. A major limitation of these compounds is that their reactivity is indiscriminate and can, in principle, occur in healthy tissue as well as cancerous tissue, potentially leading to side effects in vivo. Here we present pH-dependent intramolecular coordination of an arene-tethered sulfonamide functionality in organometallic ruthenium(II) ethylenediamine complexes as a route to controlling the coordination environment about the central metal atom. Through variation of the sulfonamide R group and the length of the tether linking it to the arene ligand the acidity of the sulfonamide NH group, and hence the pH-region over which regulation of metal coordination occurs, can be modulated. Intramolecular sulfonamide ligation controlled the reactivity of complex **4** within the physiologically relevant pH-region, rendering it more reactive towards 5ʹ-GMP in mildly acidic pH-conditions typical of tumour tissue compared to the mildly alkaline pH-conditions typical of healthy tissue. However, the activation of **4** by ring-opening of the chelate was found to be a slow process relative to the timescale of typical cell culture assays and members of this series of complexes were found not to be cytotoxic towards the HT-29 cell line. These complexes provide the basis for the development of analogues of increased potency where intramolecular sulfonamide ligation regulates reactivity and therefore cytotoxicity in a pH-dependent, and potentially, tissue-dependent manner.

## 1. Introduction

Metallodrugs offer much potential in the development of new chemotherapeutics for the treatment of cancer [1,2,3,4]. The careful choice of the metal ion, controlling properties such as coordination geometry, metal oxidation state and redox chemistry, offers the potential of modes of activity not possible with purely organic chemotherapeutics. Metallodrugs that exert anticancer activity often do so through coordination to a variety of biomacromolecular targets. However, a major limitation is that such coordination can occur in both cancerous and healthy tissue, leading to off-target reactivity that would lead to potentially serious side effects in vivo. Rendering metal-centered reactivity dependent on the local tissue environment could lead to reduced off-target reactivity, for example metallodrugs with increased reactivity in tumors could be devised by exploiting the difference in extracellular pH between healthy (7.2–7.4) and cancerous tissue (6.5–6.9) [5]. Several studies have reported metallodrugs possessing reactivity profiles that fulfil some of these aims where the coordination environment around the central metal atom has been rendered pH-dependent.

**Figure 1:**
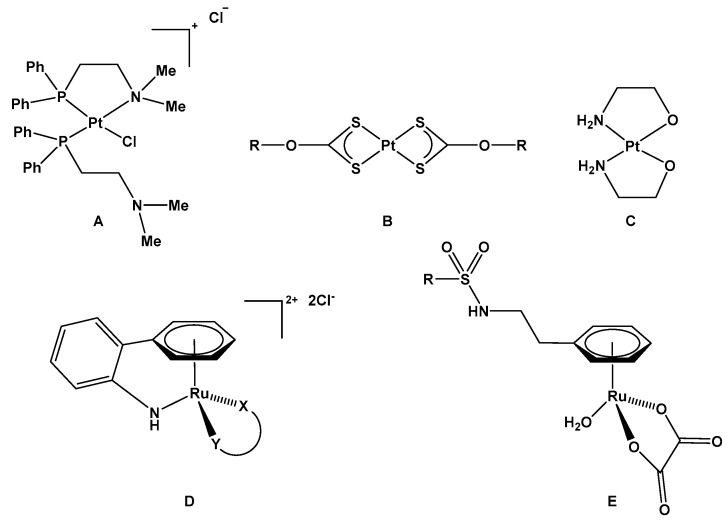
Examples of metallodrugs previously investigated for their pH-dependent reactivity (**A**) *cis*-[PtCl(Me_2_N(CH_2_)_2_PPh_2_-*N*,*P*)(Me_2_N(CH_2_)_2_PPh_2_-*P*)]Cl; (**B**) the general structure of bis(*O*-alkyldithiocarbonato)platinum(II) complexes; (**C**) (*SP*-4-2)-bis(2-aminoethanolato-κ^2^ *N*,*O*)platinum(II); (**D**) general structure of [Ru{η^6^:κ^1^-C_6_H_5_(C_6_H_4_)NH_2_}(XY)]^2+^ complexes; (**E**) structure of [Ru(η^6^-C_6_H_5_CH_2_CH_2_NHR)(C_2_O_4_)(H_2_O)] (R = Ms, Tf) complexes.

Sadler et al. explored the reversible and pH-dependent equilibrium between ring-open and ring-closed forms of bis(aminophosphine) Pt(II) complexes [6,7]. pH titrations with *cis*-[PtCl(Me_2_N(CH_2_)_2_PPh_2_-*N*,*P*)(Me_2_N(CH_2_)_2_PPh_2_-*P*)]Cl (Figure 1A) yielded a p*K*_a_ value of 6.9 and the equilibrium was also found to be dependent on Cl^−^ concentration. *cis*-[PtCl(Me_2_N(CH_2_)_2_PPh_2_-*N*,*P*)(Me_2_N(CH_2_)_2_PPh_2_-*P*)]Cl was reported to be active against several cell lines, in conditions in which the open form of the complex would be present, but both *cis*-[PtCl(Me_2_N(CH_2_)_2_PPh_2_-*N*,*P*)(Me_2_N(CH_2_)_2_PPh_2_-*P*)]Cl and the ring closed form were found to react rapidly with 5′-GMP. This indicates the closed form of the complex is unable to regulate coordination of DNA to the metal center, although coordination of *S*-donor ligands to Pt(II) was prevented in the ring-closed form of the complex. A series of bis(*O*-alkyldithiocarbonato)platinum(II) complexes (Figure 1B) were found to be more active at pH 6.8 versus 7.4 with average IC_50_ (pH 7.0)/IC_50_ (pH 6.8) ratios >2 found across six tumor cell lines for selected complexes [8]. The increase in activity of the complexes at acidic pH was attributed to increased ligand protonation and consequential destabilization of the complex, leading to increased reactivity. A bis(2-aminoethanolato-κ^2^ *N*,*O*) platinum(II) complex (Figure 1C) was also shown to exhibit increased reactivity toward 5ʹ-GMP at pH 6.0 versus pH 7.4 [9], although reactivity under the latter pH conditions was still appreciable. Reactivity towards DNA was not detected at pH 7.4 but found to be greatly increased at pH 6.0 [10] and cell culture experiments revealed increased cytotoxicity toward the A549 cell line at pH 6.0 versus 7.0 [9]. Platinum(II) complexes based on 1,3-dihydroxyacetone oxime ligands were also shown to exhibit pH-dependent cytotoxicity profiles against the SW480 and A549 cell lines (pH 6.0 versus pH 7.4), again related to a pH-dependent ring-opening activation process to increase the reactivity of the complex [11].

The pH-dependent intramolecular chelation of a range of ligands in half-sandwich ruthenium complexes to regulate ligand coordination at the ruthenium center has also been explored. Scrase et al. reported that a L-2,3-diaminopropionic acid ligand coordinated in a tridentate manner to a ruthenium-η^6^-arene moiety was found to be labilized on lowering of pH, leading to partial reactivity of the complex on incubation with a protected methionine ligand at pH 2.5 [12]. Pizarro et al. have shown reversible intramolecular chelation can be achieved in half-sandwich ruthenium complexes utilizing an hemilabile 2-aminobiphenyl arene ligand [13]. The closed form of the complexes, achieved through an arene ligand η^6^:κ^1^-C_6_H_5_(C_6_H_4_)NH_2_ coordination mode (Figure 1D), dominates at neutral pH (the p*K*_a_ value for the pendant amine functionality was found to be ca. 2.5), and were found to be unreactive with respect to coordination to 5′-GMP. The complexes were found to be non-cytotoxic in cell culture experiments, in accordance with the lack of reactivity observed under these pH conditions. Pizarro et al. have also developed ruthenium(II) piano-stool complexes utilizing phenylacetic acid as the arene ligand, enabling a 5-membered intramolecular chelate to be formed via carboxylate *O*-coordination to ruthenium [14]. Several complexes of the structure [Ru(η^6^-C_6_H_5_CH_2_COOH)(XY)Cl]Cl/Na were devised and -COOH p*K*_a_ values in the range 2.8–3.5 were reported, indicating the closed form of the complexes predominates under physiologically relevant conditions. Interestingly the closed form of some of the complexes reported were mildly cytotoxic, potentially indicating an alternative cytotoxic mechanism of action to metallodrug-biomacromolecular coordination. In a previous study we evaluated the utility of a pendant sulfonamide functionality in [Ru(η^6^-C_6_H_5_CH_2_CH_2_NHR)(C_2_O_4_)(H_2_O)] (R = Ms, Tf) complexes (Figure 1E) to regulate the availability of a coordination site at ruthenium, and subsequently the ligation of 5′-GMP to the metal as a function of pH [15]. We found that modulation of the sulfonamide R group enabled the basicity of the sulfonamide nitrogen to be tuned, and when R=CH_3_ the apparent p*K*_a_ was found to be 6.65. Consequently, coordination of 5′-GMP to ruthenium was found to be able to be controlled across the physiologically relevant pH region. Interestingly, L-histidine coordination was not regulated under these conditions and was found to displace the ligated sulfonamide. This family of complexes therefore offers potential selectivity with respect to ruthenium coordination between different classes of biomacromolecular targets but, due to a lack of pH dependence with respect to L-histidine binding, are unable to differentiate between healthy and cancerous tissue.

In this work we expand the range of half-sandwich ruthenium structures into which pendant sulphonamide functionality has been incorporated. We hypothesized that the introduction of pendant sulfonamide functionality into organometallic structures known to be cytotoxic towards cancer cell lines would regulate their reactivity, and therefore cytotoxicity, in a pH-dependent manner that is not achievable with the original complex. We chose to focus on the [Ru(η^6^-arene)(en)(Cl)]PF_6_ (en = ethylenediamine) family of compounds [16] that are known to be cytotoxic towards a range of cancer cell lines with IC_50_ values typically in the low µM range [17]. Here we report the synthesis of a series of ruthenium(II)-arene complexes of the type [Ru(η^6^-C_6_H_5_(CH_2_)_n_NHR)(en)(Cl)]PF_6_, with pendant sulfonamide or N-acetyl functionality, and evaluate their pH-dependent reactivity in aqueous solution and their cytotoxicity in cell culture experiments.

## 2. Results and Discussion

### 2.1. Synthesis and Characterisation

**Figure 2:**
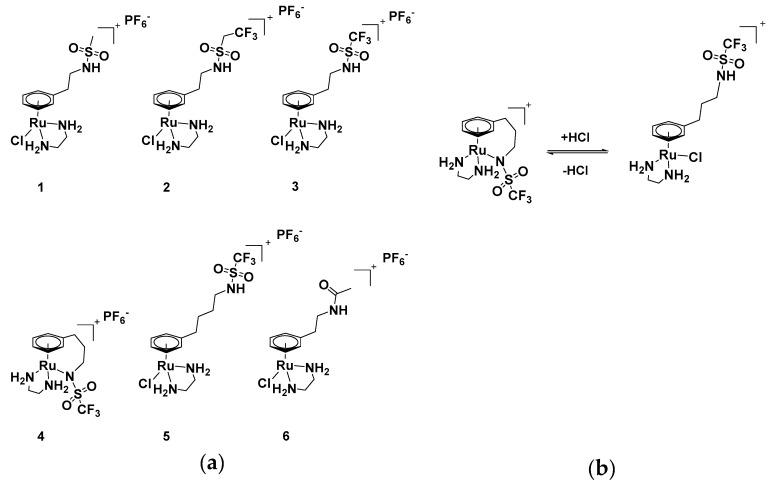
(**a**) Compounds **1**–**6**; (**b**) Reversible pH-dependent intramolecular sulfonamide ligation with **4**.

A total of six half-sandwich ruthenium(II) arene complexes were synthesized for evaluation in this study (Figure 2). [Ru(η^6^-C_6_H_5_CH_2_CH_2_NHR)(en)(Cl)]PF_6_ (R = Ms (**1**), tresyl (Tr) (**2**), Tf (**3**) or acetyl (**6**)) and [Ru(η^6^-C_6_H_5_(CH_2_)_3_CH_2_NHTf)(en)(Cl)]PF_6_ (**5**) were prepared via reaction of the respective [RuCl_2_(η^6^-C_6_H_5_(CH_2_)_n_CH_2_NHR)]_2_ (R = acetyl, Ms, Tf or Tr) complex with ethylenediamine followed by the addition of NH_4_PF_6_ then crystallization of the product by concentration of the methanol solution or via vapor diffusion with diethyl ether. [Ru(η^6^:κ^1^-C_6_H_5_(CH_2_)_2_CH_2_NTf)(en)]PF_6_ (**4**) was prepared via the reaction of [RuCl_2_(η^6^-C_6_H_5_(CH_2_)_2_CH_2_NHTf)]_2_ with ethylenediamine then treatment with silver carbonate and triethylamine, followed by addition of NH_4_PF_6_ and precipitation of the product by concentration and cooling. All complexes are soluble in water (≥5 mM) and stable in the presence of NaCl (100 mM). All complexes were characterized by ^1^H, ^13^C and ^19^F NMR spectroscopy, high resolution mass spectrometry and elemental analysis.

### 2.2. Crystallography

Single crystals of **1**–**6** suitable for single crystal X-ray crystallography were obtained during the crystallization step of the bulk complexes.

**Figure 3:**
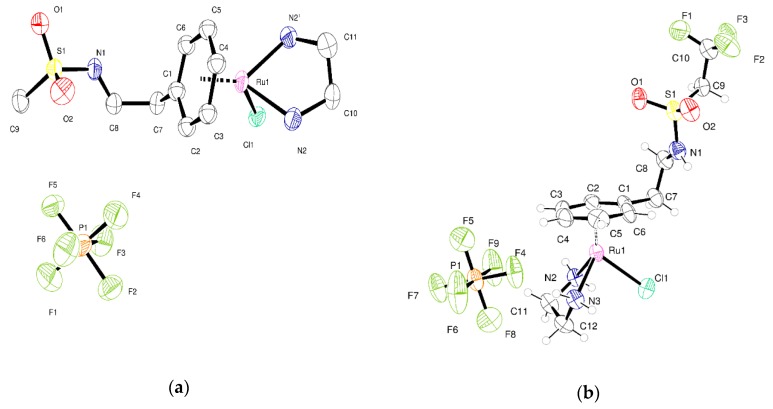
(**a**) ORTEP plot of complete asymmetric unit of **1** with atoms shown as 50% displacement parameters; (**b**) Asymmetric unit of **2** at 150 K with atoms drawn as 50% displacement ellipsoids.

The molecular structure of **1** is rather simple as shown in the ORTEP plot in Figure 3a. However, each of the ions present (PF_6_^−^ and [RuCl(en)L]^+^) lies on one of the mirror planes present in the space group P2_1_/m. For the metal-containing fragment, this mirror plane passes mid-way through the ethylenediamine and mid-way through the arene ligand. The action of the mirror plane generates a second orientation of ligands related to the first by rotation of 23.3 degrees. This provides a beautiful but most unwelcome complication to the modelling of the crystal structure (illustrated in Figure S1). Two orientations of all the atoms present except Ru1, Cl1 and C1 are generated by symmetry. The unit cell contains two molecules of the compound related by inversion symmetry. Attempts made to refine the structure in the space group P2_1_ were unsatisfactory and the disorder persisted. The intensity statistics and refinements suggest that the centrosymmetric space group P2_1_/m is correct. N–H···Cl interactions assemble the molecules into tapes that extend along the crystallographic b-axis. These interactions are augmented by N–H···F interactions with PF_6_ anions.

In **2** the Ru is surrounded by the arene, one chloride, and bidentate ethylenediamine. The structure is centrosymmetric and crystallizes in space group P$\bar{1}$ with a single complex and one anion in the asymmetric unit as shown in Figure 3b. Adjacent complexes are held together by two N–H···Cl hydrogen bonds from ethylenediamine in a centric R^2^_2_(8) embrace [18]. There are further N–H···O=S and N–H···Cl interactions between the amide and sulfonamide and chloride. These interactions form tapes of ruthenium complexes that run parallel to the crystallographic *a* direction. Between the tapes lie PF_6_^−^ anions. There are C–H···F interactions between the alkyl chain of the complex and these anions and also between alkyl chain and the CF_3_ groups. The crystal examined was a non-merohedral twin with the two components related by the twin law (1.01 −0.035 0.012; 0.0534 0.985 0.003; 0.002 −0.01 1.003). The integration process produced intensity data from two domains and overlapping reflections. The structure was refined using all observed data using the HKLF5 formalism within SHELXL. The two domains were present in the ratio 0.622:0.378(2).

**Figure 4:**
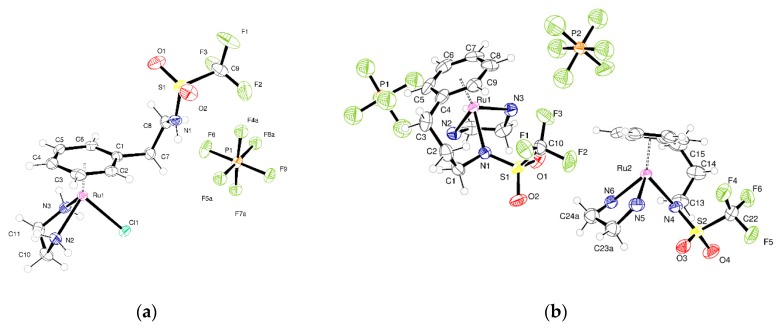
(**a**) Asymmetric unit of **3** with atoms shown as 50% displacement parameters (small-scale disorder in the anion is not shown); (**b**) Asymmetric unit of **4** with atoms shown as 50% displacement parameters (small-scale disorder in the anion is not shown).

For **3** (Figure 4a) the molecular structure differs from **2** only in the sulfonate group. Here, the CF_3_ is directly joined to the sulfur rather than a CH_2_CF_3_ group being joined to the sulfur. This has a pronounced influence on the interactions between molecules and hence the crystal packing. Coordination about the metal is similar to other examples. The N–H···Cl interactions from the bound ethylenediamine do not occur in this structure; one N–H···O=S hydrogen bond is present. There are N–H (amide)···Cl interactions that form dimers of molecules. These dimers are held into a puckered 2-D network by the N–H···O=S hydrogen bonds. The space between networks is filled by the anions.

Compound **4** crystallizes in the monoclinic space group P2_1_/n with two Ru complex ions and two PF_6_^−^ anions in the asymmetric unit (Z′ = 2) (Figure 4b). The Z′ = 2 structure persists at room temperature. There is small-scale disorder in the position of the carbons of the ethylenediamine ligand attached to Ru2. This corresponds to two different orientations of the carbon backbone. Each PF_6_^−^ anion is disordered over two positions. It was possible to identify these from the difference Fourier maps and model these groups using standard techniques. The crystal structure has Laue symmetry 2/m but it is twinned to mimic the Laue symmetry mmm. In terms of the unit cell, the primitive monoclinic structure is twinned to mimic a *C*-centered orthorhombic cell by the twin law that converts *a*, *b*, *c* into *c*, −*b*, *a.* This pseudo-merohedral twinning was allowed for in the refinement of the structure. The ratio of the major and minor components is 0.9680:0.0320(7). This led to a significant improvement in the quality of the fit on F^2^ and an improvement in the C–C bond precision. Allowing for twinning, wR(F^2^) (all data) decreased from 0.1562 to 0.1445 and the C−C precision improved from 0.0081 to 0.0073 Å. There are no classical hydrogen bonds present in the structure, but C–H···O and C–H···F interactions exists between the species present to generate a dense network of intermolecular interactions that extends in the 3-D.

**Figure 5:**
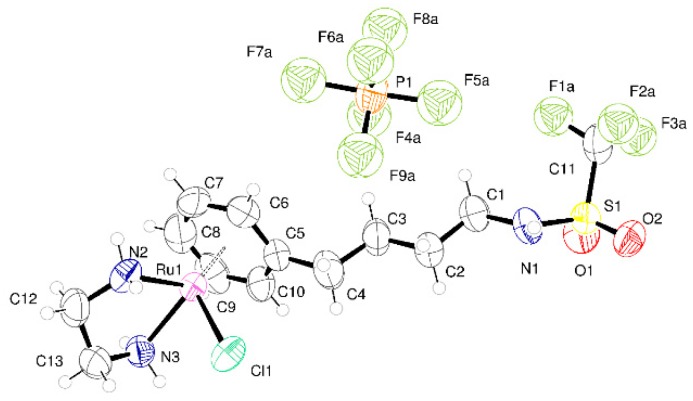
Asymmetric unit of **5** with atoms shown as 50% displacement parameters (small-scale disorder in the anion and CF_3_ group are not shown).

**5** crystallizes in the centric space group P2_1_/n with a single ion pair in the asymmetric unit as shown in Figure 5. As in previous examples there is a centric R^2^_2_(8) embrace formed from two N–H···Cl hydrogen bonds from ethylenediamine on adjacent molecules. Additionally, each molecule forms two more identical R^2^_2_(8) embraces to another molecule reflated by inversion, through the N–H(amide)···Cl and N–H(diamine)···O=S. These two sets of interactions produce tapes of molecules that run parallel to the crystallographic *a* direction. Between these tapes lie the PF_6_^−^ anions and there are C–H···F interactions present.

**Figure 6:**
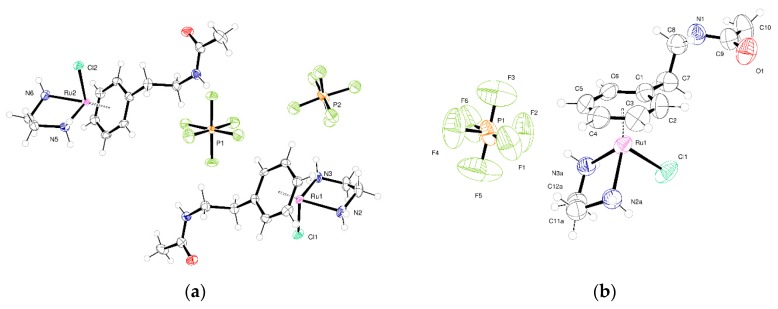
(**a**) Asymmetric unit of **6** at 100 K with atoms drawn as 50% displacement ellipsoids (small-scale disorder in one of the PF_6_^−^ groups is not represented). (**b**) Asymmetric unit of **6** at 298 K with atoms drawn as 50% displacement ellipsoids (small-scale disorder in the ethylene diamine is not shown).

At low temperature compound **6** crystallizes with two independent ruthenium complexes in the asymmetric unit and two hexafluorophosphate anions. At 100 K and 150 K, the structure crystallizes in space group Cc and has Z′ of 2 as shown in the ORTEP in Figure 6. The coordination about the two ruthenium ions is very similar and the structure displays pseudo-symmetry, mimicking the higher symmetry space group C2/c. Adjacent pairs of independent complexes are held together by two N–H···Cl hydrogen bonds in a pseudo-centric R^2^_2_(8) embrace. These dimers are further linked by the presence of N–H···O=C hydrogen bonds. This gives a dense network of complexes in 3-D within which are located the anions. At higher temperature the pseudo symmetry operation is replaced by a strict one and the structure crystallizes with a single ruthenium complex in the asymmetric unit in space group C2/c. The structure at 298 K is shown in Figure 6. The same crystal was examined at 298 K and 100 K without being remounted. It is clear from refinements and comparisons of the bond precision at different temperatures that the true cell at low temperature has Z′ = 2 while that 298 K is Z′ = 1. This increase in symmetry with increase in temperature is common; one recent example shows two phase changes associated with increasing symmetry upon heating [19]. At 298 K the same packing of the complexes as the low temperature form is present, based on truly centrosymmetric hydrogen-bonded dimers.

### 2.3. Solution Studies

**Figure 7:**
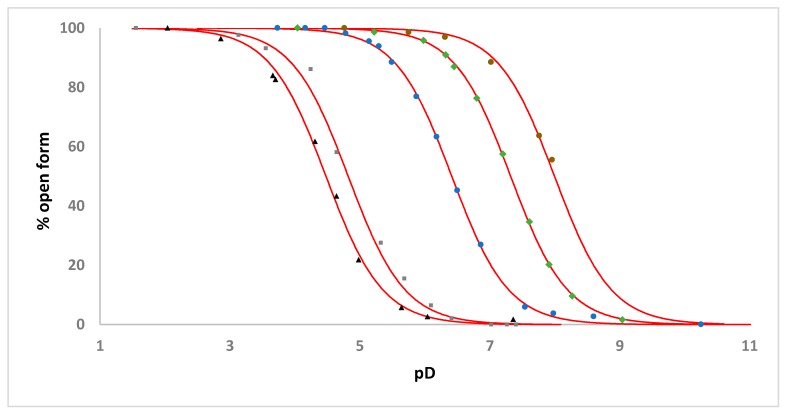
pH dependence of intramolecular sulfonamide ligation for **1** (green diamonds), **2** (blue circles), **3** (black triangles), **4** (grey squares) and **5** (brown circles) (2.46 mM complex, D_2_O, 295 K, 0.1 M NaCl) (Appendix A). pD values are determined using pD = pH meter reading +0.4 [20]. The solid red lines represent the fit of the Henderson-Hasselbalch equation to each set of experimental data.

**Figure 8:**
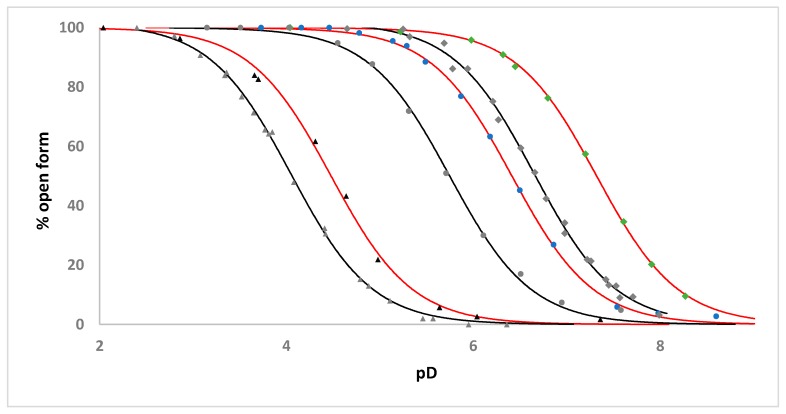
pH dependence of intramolecular sulfonamide ligation for **1** (green diamonds), **2** (blue circles), **3** (black triangles) and the analogous [Ru(η^6^-C_6_H_5_CH_2_CH_2_NHR)(C_2_O_4_)(H_2_O)] complexes [Ru(η^6^-C_6_H_5_CH_2_CH_2_NHTf)(C_2_O_4_)(H_2_O)] (grey triangles), [Ru(η^6^-C_6_H_5_CH_2_CH_2_NHTr)(C_2_O_4_)(H_2_O)] (grey circles) and [Ru(η^6^-C_6_H_5_CH_2_CH_2_NHMs)(C_2_O_4_)(H_2_O)] (grey diamonds) (2.46 mM complex, D_2_O, 295 K, 0.1 M NaCl). pD values are determined using pD = pH meter reading +0.4 [20]. The solid lines represent the fit of the Henderson-Hasselbalch equation to each set of experimental data.

The ability of the tethered sulfonamide functionality to reversibly ligate to the ruthenium atom in a pH-dependent manner, forming an intramolecular chelate, was examined by recording ^1^H NMR spectra of **1**–**5** (and ^19^F NMR spectra for **2**–**5**) (2.46 mM complex, D_2_O, 298 K, 0.1 M NaCl) as a function of pH. Within these spectra two unique sets of signals were observed, the relative proportion of these being pH-dependent, that were assigned to the open and chelate forms of the complexes (Figures S12–S15). Plotting the percentage of open form of the complex, calculated from the ratio of selected ligand signals corresponding to the open and chelate forms of the complexes, versus pD yielded sigmoidal curves in each case and apparent p*K*_a_ values for **1**–**5** (Table 1) were determined by fitting of the Henderson-Hasselbalch equation to the data (Figure 7 and Figure 8). 

The increasingly electron-withdrawing nature of the sulfonamide R substituent on going from **1**–**3** is reflected by the decrease in the apparent p*K*_a_ values of 7.32, 6.41 and 4.50 respectively-highlighting the ability to modulate the basicity of the sulfonamide nitrogen through choice of R group. It is worthwhile comparing these apparent p*K*_a_ values to those for the analogous [Ru(η^6^-C_6_H_5_CH_2_CH_2_NHR)(C_2_O_4_)(H_2_O)] complexes we have previously reported that bear oxalate ligands in place of the ethylenediamine ligand (Table 1, Figure 8) [15]. The presence of the dianionic oxalate ligand results in an apparent p*K*_a_ value 0.45–0.67 pH units lower than in the analogous en complex and reflects the influence of increased electron density at the metal center on sulfonamide nitrogen coordination.

A comparison of the apparent p*K*_a_ values obtained for **3**–**5** shows that as the length of the tether between the arene ligand and the sulfonamide group increases the apparent p*K*_a_ also increases (Appendix A, Table 1, Figure 7), likely reflecting the reduction in intramolecular chelate ring strain as tether length increases as well as the decreasing proximity of the sulfonamide nitrogen to the metal center.

During these studies it was observed that for **1**–**3** arene-ruthenium cleavage began to occur on standing of the NMR solution for even relatively short periods of time (>1 h) (Figures S12–S14). In contrast, arene loss was not observed with **4** and **5**, even on standing for extended periods of time (>24 h), and most likely reflects the reduced ring strain experienced by the latter complexes when in their chelate form compared to **1**–**3** possessing a shorter tether. ^1^H NMR spectra of **6** recorded as a function of pH did not reveal any pH-dependent behavior across the physiologically relevant pH region and the amide nitrogen was unable to coordinate to ruthenium under any of the conditions evaluated (Figure S11).

The coordination of a metallodrug, via the metal ion, to biomacromolecular targets is a major mechanism by which biological activity is exerted in many of these complexes. To evaluate the ability of intramolecular sulfonamide ligation to regulate the availability of a coordination site at the ruthenium ion in this family of complexes, and therefore control ligation of endogenous ligands to the complex, binding studies were performed between **4** (and the control compound **6**) and the model ligands L-histidine and 5′-GMP. **4** and **6** were incubated with 1 eq. of 5′-GMP or L-histidine (2.46 mM complex, phosphate buffer (0.2 M), 310 K, 0.1 M NaCl) for 144 h at pD values of 7.5 and 6.5 in each case. Incubations were monitored by ^1^H and ^19^F NMR. Intramolecular sulfonamide ligation in **4** was found to regulate the coordination of 5′-GMP or L-histidine significantly compared to **6**. For example, at a pD value of 7.5 no coordination of 5′-GMP or L-histidine to **4** was observed after 144 h incubation (Figures S16 and S19) and the sulfonamide group remained coordinated to ruthenium. In contrast, at a pD value of 6.5 5′-GMP was observed to be able to coordinate to **4**—16 ± 1.6% (n = 4) of 5′-GMP was found to be coordinated after 144 h incubation (Figure S17), highlighting the increased proportion of the open form of **4** present in these pD conditions. These spectra also show very weak signals attributed to the release of minor quantities of free arene ligand, indicating that on coordination of 5′-GMP to **4** under these conditions a further decomposition pathway may be established—similar decomposition was not observed in the incubations between **4** and 5′-GMP at pD 7.5 or **6** and 5′-GMP at pD values of 6.5 or 7.5. No coordination of L-histidine to **4** was observed at pD 6.5 (Figure S20), even after 144 h incubation. **6**, which possesses an arene-tethered amide group that does not ligate to ruthenium to form an intramolecular chelate, was found to be significantly more reactive toward 5′-GMP and L-histidine. 41% and 64% of 5′-GMP was found to coordinate to **6** at pD values of 7.5 and 6.5 respectively after 144 h incubation (Figure S18). L-histidine was also found to coordinate to **6** under both pD conditions, as assessed by ^1^H NMR (Figure S21) and visualized through the darkening of the color of the solution - this was not observed with **4**. The degree of L-histidine coordination was not able to be quantified using ^1^H NMR due to hydrogen-deuterium exchange which occurs at the histidine C2 proton [21] and low intensity and overlapping peaks, but was more pronounced at pD 7.5 than pD 6.5. This is presumably due to appreciable L-histidine imidazole protonation under the latter conditions (side chain p*K*_a_ = 6.07 [22]) which contrasts with 5′-GMP where the N7 position (p*K*_a_ = 2.57 [23]) is not protonated across the pH range examined. We further incubated **4** with bovine serum albumin (2.46 mM complex, 0.5 mM bovine serum albumin, phosphate buffer (0.2 M), 310 K, 0.1 M NaCl, pD 7.4) to assess the stability of the intramolecular chelate form of the complex in the presence of protein. After 72 h ^1^H (and ^19^F) NMR (Figure S22) analysis revealed no significant changes (no loss of the intramolecular chelate, no new arene signals) indicating no covalent interaction between **4** and the protein. However, ^1^H NMR signals for **4** were broader and slightly shifted relative to a control sample suggesting non-covalent interactions between **4** and the protein may be occurring.

**Figure 9:**
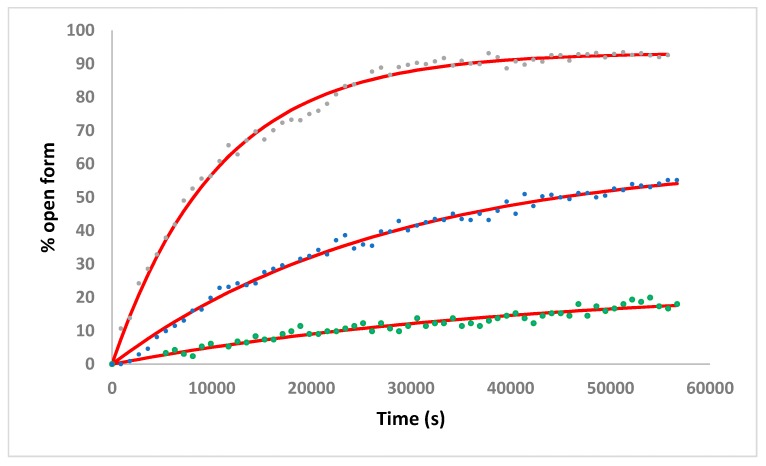
Time dependence of the conversion of the chelate form of **4** to the open form. pD 4.12 (grey circles), pD 4.91 (blue circles) and pD 5.96 (green circles) (2.46 mM complex, citrate/phosphate buffer, 310 K, 0.1 M NaCl). The solid red lines represent the fit of the equation y = C + A(1 − e^−kx^) (C-offset from zero; A-amplitude of the curve) to each set of experimental data.

To gain further insight into the reactivity of **4** the conversion of the chelate form to open form of the complex was probed in three different citrate/phosphate buffer solutions at 310 K (Figure 9). Aqueous solutions of **4** at pD 7.61 (where the chelate form of the complex exists exclusively) were diluted into citrate/phosphate buffer to yield solutions at pD 4.12, 4.91 and 5.96 (2.46 mM complex, 0.1 M NaCl). The conversion of the chelate form to the open form was monitored by ^19^F NMR spectroscopy over 16 h. Under the most acidic conditions (pD 4.12) equilibrium was reached within the 16 h experiment duration, with 93% of **4** converting to the open form. In the more basic buffer conditions equilibrium was not reached within 16 h—data fitting indicated that 61% and 24% of the open-form of **4** would be present at equilibrium in pD conditions of 4.91 and 5.96 respectively. The half-life for the fraction of the chelate-form of **4** which is converted to the open-form within these equilibration reactions was found to be 177, 457 and 695 min at pD values of 4.12, 4.91 and 5.96 respectively. These results highlight that the activation of **4** through ring-opening of the intramolecular chelate is much faster in more acidic pH-conditions and indicates why the reaction of the complex with 5′-GMP at pD 6.1 was only observed after extended incubation times.

### 2.4. Evaluation of In Vitro Anticancer Activity

Having established the ability of **4** to coordinate to biologically relevant ligands in a pH-dependent manner we evaluated the cytotoxicity of **4** (and **6** as a control unable to form an intramolecular chelate) against the HT-29 cell line using the MTT assay. In light of the potential non-covalent interaction of **4** with protein as described earlier these assays were performed for both **4** and **6** using media containing 10% and 1% fetal calf serum to assess the effect of serum concentration on cytotoxicity. In each case no significant inhibition of cell growth was observed following cell exposure to the complexes at concentrations up to 100 µM for 72 h. It is clear that while **6** is readily able to coordinate to 5′-GMP and L-histidine, and presumably forms biomacromolecular adducts in cell culture experiments, the nature and/or level of adducts formed is not detrimental to HT-29 cell viability. The ability of **4** to form biomacromolecular adducts in cell culture conditions will be less than that of **6** due to substantial intramolecular sulfonamide ligation under these pH conditions. It is therefore not unexpected that **4** exhibits no cytotoxicity towards the HT-29 cell line. The lack of toxicity of **6** is surprising given that low IC_50_ values (typically <20 µM) are well documented for the [Ru(η^6^-arene)(en)(Cl)]PF_6_ family of compounds [17], consequently further experiments are required to probe the origins of this behavior (such as uptake and intracellular localization studies) but the MTT assays indicate that this may not be due to deactivation through protein binding.

## 3. Conclusions

In summary, reversible intramolecular sulfonamide ligation has been extended to the [Ru(η^6^-arene)(en)(Cl)]PF_6_ family of complexes and has been found to be an effective method of regulating the coordination environment at the central metal atom in a pH-dependent manner. Variation of the sulfonamide R group and the length of the tether linking the arene ligand and sulfonamide functionality was shown to modulate the apparent p*K*_a_ of the sulfonamide proton, and hence the pH region over which regulation of metal coordination occurs. This was exemplified in **4** by the regulation of ruthenium coordination to model ligands and bovine serum albumin over the physiologically relevant pH region. However, the slow ring-opening of the chelate form of **4** to activate the complex is a limitation of these systems and would not enable complete activation/deactivation of the complex within a suitable timescale on transfer between the different pH environments within biological systems. The lack of cytotoxicity exhibited by **6**, a complex expected to form appreciable levels of biomacromolecular adducts in cell culture experiments, was surprising and further experiments are required to uncover the origins of this behavior and to modify structural elements of these complexes so that in the open form they are as active as previously reported [(η^6^-arene)Ru(en)X]^n+^ compounds. For example, analogous complexes could be devised bearing extended planar arene ligands that would promote Ru-N7-guanine coordination in DNA, as reported for the [(η^6^-arene)Ru(en)X]^n+^ series of complexes [23], and potentially lead to more potent complexes. The complexes reported within therefore provide a basis for the further development of potent anticancer compounds with pH-dependent reactivity profiles dictated by reversible intramolecular sulfonamide ligation.

## 4. Materials and Methods

### 4.1. Materials

All commercially purchased materials were used as received. Ethylenediamine, guanosine 5′-monophosphate disodium salt and ruthenium(III) chloride hydrate were purchased from Fluorochem Ltd. (Hadfield, UK). L-histidine was purchased from Acros Organics (Geel, Belgium). Silver carbonate was purchased from Alfa Aesar (Heysham, UK). Ammonium hexafluorophosphate, citric acid, deuterium chloride (20 wt. % solution in D_2_O), sodium deuteroxide (40 wt. % solution in D_2_O), sodium phosphate dibasic heptahydrate, sodium sulfate, trimethylamine and 2,2,2-trifluoroethanesulfonyl chloride were purchased from Fisher Scientific (Loughborough, UK). Dichloromethane, ethanol and methanol were purchased from Honeywell (Morristown, NJ, USA). Diethyl ether was purchased from VWR (Lutterworth, UK). Chloroform-*d*, dimethylsulfoxide-*d*_6_ and deuterium oxide were purchased from Eurisotop (Saint-Aubin, France).

### 4.2. Instrumentation and Methods

#### 4.2.1. NMR

^1^H, ^13^C and ^19^F NMR spectra were recorded on a Jeol JEOL ECZ 400S spectrometer (^1^H at 400.2 MHz, ^13^C at 100.6 MHz and ^19^F at 376.5 MHz) (Welwyn Garden City, UK). Spectra are referenced internally to residual solvent peaks (D_2_O: ^1^H *δ* 4.79 ppm, ^13^C *δ* unreferenced; DMSO-*d*_6_: ^1^H *δ* 2.50 ppm, ^13^C *δ* 39.52 ppm); ^19^F NMR spectra are reported relative to a CFCl_3_ reference (*δ* 0 ppm). Spectra were acquired at 295 K unless stated otherwise.

#### 4.2.2. Mass Spectrometry

Accurate mass measurements were performed at the University of Hull using a Bruker Maxis Impact QqTOF MSMS (Coventry, UK). Before mass measurement the instrument was calibrated against sodium formate over the range 90 to 1550 Da. Resolution used was typically 45000. Samples (as solutions in methanol, 10–5 M) were injected into a solvent stream from a syringe pump at 3 µL min^−1^ via a 5 µL loop injector. The data was then internally mass measured against an internal calibrant peak from hexakis(1*H*,1*H*,4*H*-hexafluorobutyloxy)phosphazine (CAS No. 186406-47-2) C_24_H_18_O_6_N_3_P_3_F_36_ *m/z* 1220.99064. An average result from 3–5 separate injections is quoted. The mass was measured and calculated using Bruker DataAnalysis 4.2 software (Coventry, UK).

GCMS. A Perkin-Elmer Autosystem XL GC coupled with a Perkin-Elmer Turbomass (Seer Green, UK) quadrupole MS was used, equipped with a Restek Rxi-1MS column 30 m (l) × 0.25 mm (i.d.) 0.25 µm film thickness. He was used as the carrier gas (mobile phase) at 1 mL·min^−1^. A 1.0 µL sample injection volume with 20:1 split was employed. The scan range spanned *m/z* 600 to *m/z* 30 in 1 sec in conjunction with positive mode electron ionization at 70 eV.

#### 4.2.3. X-ray Crystallography

Routine X-ray diffraction intensity data were collected in series of ω-scans using a Stoe IPDS2 image plate (Darmstadt, Germany) diffractometer operating with Mo Kα radiation at 150(2) K (290(2) K for **5** and 100(2) K for **6**). The data were corrected for the effects of absorption using a multi-scan method [24]. The structures were solved using dual-space methods within SHELXT and full-matrix least squares refinement against F^2^ was carried out within SHELXL-2014 or SHELXL-2018 via the WinGX program interface [25,26]. All non-hydrogen positions were located in the direct and difference Fourier maps and refined using anisotropic displacement parameters. Hydrogen atoms were placed using a riding model. Crystal structure data for the compounds reported here are summarized in the ESI. Crystal structure data (CCDC 1963883, 1963884, 1963885, 1963886, 1963887, 1963888 and 1963889) are available free of charge from the CCDC via https://www.ccdc.cam.ac.uk/structures/.

#### 4.2.4. Elemental Analysis

Elemental analysis was performed at the University of Hull. Analysis for all compounds was performed with the addition of V_2_O_5_.

### 4.3. Synthesis

[Ru(η^6^-1,1,1-trifluoro-*N*-phenethylmethanesulfonamide)Cl_2_]_2_ [27], [Ru(η^6^-1,1,1-trifluoro-*N*-(3-phenylpropyl)methanesulfonamide)Cl_2_]_2_ [27], [Ru(η^6^-1,1,1-trifluoro-*N*-(4-phenylbutyl)methanesulfonamide)Cl_2_]_2_ [27], [Ru(η^6^-*N*-phenethylacetamide)Cl_2_]_2_ [28], [Ru(η^6^-*N*-phenethylmethanesulfonamide)Cl_2_]_2_ [15] and 2-(cyclohexa-1,4-dien-1-yl)ethan-1-amine [29] were synthesized as previously reported.

**Complex 1:** [Ru(η^6^-*N*-phenethylmethanesulfonamide)Cl_2_]_2_ (200 mg, 0.269 mmol) was suspended in MeOH (40 mL) followed by the addition of ethylenediamine (36 µL, 0.537 mmol)-the mixture became slightly more soluble on stirring. After stirring for 2 h the reaction mixture was filtered followed by the addition of NH_4_PF_6_ (262 mg, 1.61 mmol)-the mixture was stirred for 15 min then filtered and dried under reduced pressure. The residue was resuspended in MeOH (15 mL), filtered then the volume of the solution was concentrated under reduced pressure (~4 mL) and left to cool at 4 °C. After 24 h an oily dark solid had precipitated, this was isolated by decanting the liquid and dried under reduced pressure. The isolated solvent was left to stand at 4 °C for a further 24 h where further solid precipitated—this was isolated by decanting the liquid then was dissolved in MeOH (10 mL), combined with the first batch of solid isolated and stirred until the mixture had solubilized, filtered then the solvent volume was reduced to 5 mL under reduced pressure and left to stand at 4 °C. Dark red crystals formed over 48 h-these were isolated, washed with MeOH (0.5 mL) then dried under reduced pressure. The solid was then dissolved in H_2_O (2 mL) and lyophilized to remove residual MeOH and leave the product as a yellow powder (27 mg, 0.050 mmol, 9%). ^1^H NMR (DMSO-*d*_6_, 400 MHz): *δ* = 7.17 (br, 1H, N*H*), 6.47 (br, 2H, 2 × en N*H*), 5.73 (br, 2H, 2 × Ar C*H*), 5.56 (m, 3H, 3 x Ar C*H*), 4.15 (br, 2H, 2 × en N*H*), 3.23 (m, 2H, -C*H*_2_NH-), 2.89 (br, 3H, -S-C*H*_3_), 2.61 (br, 2H, -C*H*_2_-Ar), 2.15–2.37 (m, 4H, NH_2_C*H*_2_C*H*_2_NH_2_); ^19^F (DMSO-*d*_6_, 376.5 MHz): *δ* = −70.00 (d, 6F, *J* = 712 Hz, PF_6_^−^); ^13^C{^1^H} NMR (DMSO-*d*_6_, 100 MHz): *δ* = 99.2, 82.9, 82.3, 79.1, 44.3, 42.4, 33.1; HRMS (ES^+^) *m*/*z* found 396.0087 [M]^+^ C_11_H_21_ClN_3_O_2_RuS requires 396.0080; C_11_H_21_ClF_6_N_3_O_2_PRuS (%): calcd C 24.43 H 3.91 N 7.77 S 5.93 found C 24.68 H 3.89 N 7.54 S 5.72.

***N*-(2-(cyclohexa-1,4-dien-1-yl)ethyl)-2,2,2-trifluoroethane-1-sulfonamide:** A solution of 2-(cyclohexa-1,4-dien-1-yl)ethan-1-amine (1.769 g, 14.4 mmol) and triethylamine (2.12 mL, 15.2 mmol) in DCM (150 mL) was cooled to −78 °C under a nitrogen atmosphere, followed by the dropwise addition of 2,2,2-trifluoroethanesulfonyl chloride (2.500 g, 13.7 mmol) as a solution in DCM (25 mL) over 30 min. The reaction was left for 20 h and allowed to return to room temperature. The reaction mixture was extracted with HCl_(aq)_ (0.5 M, 175 mL), the aqueous phase then backwashed with DCM (160 mL) before the combined organics being dried with Na_2_SO_4_ and filtered. The solution was then dried under reduced pressure affording an orange oil containing the crude product, which was used in the following steps without further purification (3.240 g). ^1^H NMR (CDCl_3_, 400 MHz): *δ* = 5.71 (m, 2H, ring CH), 5.56 (m, 1H, ring CH), 4.66 (br, 1H, -N*H-*), 3.80 (q, 4H, *J* = 9.0 Hz, -C*H*_2_CF_3_), 3.29 (m, 2H, -C*H*_2_NH-), 2.57-2.74 (m, 4H, ring CH_2_), 2.27 (t, 2H, *J* = 6.5 Hz, -C*H*_2_CH_2_NH-); ^19^F (CDCl_3_, 376.5 MHz): *δ* = -62.5 (t, 3F, *J* = 8.5 Hz, C*F*_3_CH_2_-); ^13^C{^1^H} NMR (CDCl_3_, 100 MHz): *δ* = 130.6, 124.3, 123.7, 122.8, 121.7 (q, *J* = 275 Hz), 54.4 (q, *J* = 31 Hz), 40.9, 37.7, 28.4, 26.8; MS (EI^+^) *m*/*z* found 269.1 [M]^+^.

**[Ru(η^6^-2,2,2-trifluoro-*N*-phenethylethane-1-sulfonamide)Cl_2_]_2_:** A solution of crude *N*-(2-(cyclohexa-1,4-dien-1-yl)ethyl)-2,2,2-trifluoroethane-1-sulfonamide (2.5 g, 9.28 mmol) and RuCl_3_.xH_2_O (0.696 g, 3.36 mmol) in EtOH (35 mL) was heated at reflux for 18 h. During the heating a solid precipitated—this was collected by filtration after the reaction mixture was allowed to cool, washed with EtOH (10 mL) then dried under reduced pressure to leave the desired product as a crude brown powder that was used without further purification (1.08 g); ^1^H NMR (DMSO-*d*_6_, 400 MHz): *δ* = 7.95 (t, 2H, *J* = 5.5 Hz, 2 × N*H*), 6.03 (dd, 4H, *J* = 6.5, 6.0 Hz, Ar C*H*), 5.78–5.82 (m, 6H, Ar C*H*), 5.50 (d, 2H, *J* = 6.0 Hz, 2 × Ar C*H*), 4.44 (q, 4H, *J* = 10.0 Hz, 2 × -C*H*_2_CF_3_), 3.33 (-C*H*_2_NH-resonance obscured by residual solvent peak), 2.62 (t, 4H, *J* = 7.0 Hz, 2 × -C*H*_2_CH_2_NH-); ^19^F (DMSO-*d*_6_, 376.5 MHz): *δ* = −60.9 (t, 6F, *J* = 9.0 Hz, 2 × C*F*_3_CH_2_-); ^13^C{^1^H} NMR (DMSO-*d*_6_, 100 MHz): *δ* = An expected quartet -CF_3_) around 122.0 is hard to discern due to the presence of other peaks in this region, 103.3, 88.2, 86.3, 84.1, 52.4 (q, *J* = 30 Hz), 41.7, 33.0.

**Complex 2:** [Ru(η^6^-2,2,2-trifluoro-*N*-phenethylethane-1-sulfonamide)Cl_2_]_2_ (120 mg, 0.137 mmol) was suspended in methanol (30 mL) followed by the addition of ethylenediamine (18 µL, 0.269 mmol)—a green solution formed on stirring. After stirring for 2 h the reaction mixture was filtered followed by the addition of NH_4_PF_6_ (133 mg, 0.816 mmol)—the mixture was stirred for 2 h then filtered and dried under reduced pressure. The residue was resuspended in MeOH (5 mL), filtered then the volume of the solution was concentrated under reduced pressure (~3 mL) and left to cool at −18 °C. After 24 h a small amount of solid had formed, this was removed from the solution by filtration then the liquid concentrated until precipitation observed then cooled at −18 °C. Crystalline material formed over 2 h—the solvent was decanted and the solid washed with diethyl ether (2 mL) then dried under reduced pressure to leave the desired product as an yellow orange solid (91 mg, 0.149 mmol, 55%). A sample (14.6 mg) of product was dissolved in H_2_O with heating then lyophilized to remove residual MeOH and leave the product as a yellow powder. ^1^H NMR (DMSO-*d*_6_, 400 MHz): *δ* = 7.93 (br, 1H, N*H*), 6.47 (br, 2H, 2 × en N*H*), 5.72 (dd, 2H, *J* = 6.0, 6.0 Hz, 2 × Ar C*H*), 5.56 (m, 3H, 3 × Ar C*H*), 4.41 (q, 2H, *J* = 9.5 Hz, 2 × -C*H*_2_CF_3_), 4.14 (br, 2H, 2 × en N*H*), 3.30 (m, 2H, -C*H*_2_NH-), 2.61 (t, 2H, *J* = 7.0 Hz, -C*H*_2_Ar), 2.15–2.37 (m, 4H, NH_2_C*H*_2_C*H*_2_NH_2_); ^19^F (DMSO-*d*_6_, 376.5 MHz): *δ* = 0–60.93 (t, 3F, J = 10.0 Hz, C*F*_3_CH_2_-), −70.02 (d, 6F, *J* = 711 Hz, PF_6_^−^); ^13^C{^1^H} NMR (DMSO-*d*_6_, 100 MHz): *δ* = 122.5 (q, J = 275 Hz), 98.8, 82.8, 82.4, 79.1, 52.4 (q, *J* = 30 Hz), 44.3, 42.3, 33.2; HRMS (ES^+^) *m*/*z* found 463.9974 [M]^+^ C_12_H_20_ClF_3_N_3_O_2_RuS requires 463.9960; C_12_H_20_ClF_9_N_3_O_2_PRuS (%): calcd C 23.67 H 3.31 N 6.90 S 5.27 found C 23.59 H 3.57 N 7.00 S 5.20.

**Complex 3:** [Ru(η^6^-1,1,1-trifluoro-*N*-phenethylmethanesulfonamide)Cl_2_]_2_ (250 mg, 0.294 mmol) was suspended in MeOH (30 mL) followed by the addition of ethylenediamine (39 µL, 0.583 mmol)—the green mixture partially solubilized on stirring. After stirring for 2 h NH_4_PF_6_ (286 mg, 1.75 mmol) was added–the mixture was stirred for 15 min then filtered to leave an orange liquid that was dried under reduced pressure. The residue was suspended in MeOH (5 mL), filtered then left to vapor diffuse with diethyl ether. After 24 h a small quantity of amorphous solid had formed that was removed by filtration–the liquid was left to vapor diffuse with diethyl ether further. This process was repeated twice with 24 h intervals at which point orange crystals formed that were collected and washed with cold MeOH (1 mL) and dried under reduced pressure. The orange solid was then dissolved in MeOH (3 mL) and left to vapor diffuse with diethyl ether to yield crystals over 96 h—these were collected, washed with diethyl ether (2 mL) and dried under reduced pressure to leave an orange solid (114 mg, 0.192 mmol, 33%). A sample of the solid (30 mg) was dissolved in H_2_O (2 mL) then lyophilized to remove residual methanol and leave the desired product as a yellow powder. ^1^H NMR (DMSO-*d*_6_, 400 MHz): *δ* = 9.56 (br, 1H, N*H*), 6.49 (br, 2H, 2 × en N*H*), 5.73 (br, 2H, 2 × Ar C*H*), 5.56 (m, 3H, 3 × Ar C*H*), 4.18 (br, 2H, 2 × en N*H*), 3.48 (m, 2H, -C*H*_2_NH-), 2.62 (t, 2H, *J* = 7.0 Hz, -C*H*_2_Ar), 2.15–2.36 (m, 4H, NH_2_C*H*_2_C*H*_2_NH_2_); ^19^F (DMSO-*d*_6_, 376.5 MHz): *δ* = −70.01 (d, 6F, *J* = 712 Hz, PF_6_^−^), −77.21 (s, 3F, C*F*_3_-); ^13^C{^1^H} NMR (DMSO-*d*_6_, 100 MHz): *δ* = 119.5 (q weak, J = 320 Hz), 98.0, 82.8, 82.6, 79.3, 44.3, 43.5, 33.3; HRMS (ES^+^) *m/z* found 449.9815 [M]^+^ C_11_H_18_ClF_3_N_3_O_2_RuS requires 449.9797; C_11_H_18_ClF_9_N_3_O_2_PRuS (%): calcd C 22.21 H 3.05 N 7.06 S 5.39 found C 22.47 H 3.10 N 7.11 S 5.15.

**Complex 4:** [Ru(η^6^-1,1,1-trifluoro-*N*-(3-phenylpropyl)methanesulfonamide)Cl_2_]_2_ (177 mg, 0.202 mmol) was suspended in MeOH (40 mL) to yield a yellow suspension that turned green on the addition of ethylenediamine (40 µL, 0.598 mmol). The green suspension was stirred for 18 h then filtered to yield a yellow solution that was dried under reduced pressure. The residue was dissolved in MeOH (10 mL) followed by addition of Ag_2_CO_3_ (222 mg, 0.805 mmol) to yield a dark suspension that was stirred for 18 h. The suspension was then filtered and NEt_3_ (56 µL, 0.402 mmol) added to the dark orange solution followed by stirring for 20 min—the solution was filtered followed by the addition of NH_4_PF_6_ (197 mg, 1.21 mmol), stirred for 5 min then filtered. The dark solution was concentrated under reduced pressure (~4 mL) then left to stand at 4 °C to yield an orange solid (69 mg, 0.121 mmol, 60%). ^1^H NMR (DMSO-*d*_6_, 400 MHz): *δ* = 6.72 (br, 2H, 2 × en N*H*), 5.560–5.87 (m, 5H, Ar C*H*), 4.32 (br, 2H, 2 × en N*H*), 3.34 (s, residual MeOH), 3.15 (br, 2H, -C*H*_2_N-), 2.47 (br (overlapping with solvent resonance, 2H, -C*H*_2_Ar), 2.21–2.38 (br, 4H, NH_2_C*H*_2_C*H*_2_NH_2_), 1.96 (br, 2H, -CH_2_-C*H*_2_-CH_2_-); ^19^F (DMSO-*d*_6_, 376.5 MHz): *δ* = −70.01 (d, 6F, *J* = 711 Hz, PF_6_^−^), −74.77 (s, 3F, C*F*_3_-); ^13^C{^1^H} NMR (DMSO-*d*_6_, 100 MHz): *δ* = 120.6 (q, J = 327 Hz), 94.1, 85.8, 82.6, 81.9, 48.3, 44.7, 29.0, 27.7; HRMS (ES^+^) *m*/*z* found 428.0195 [M]^+^ C_12_H_19_F_3_N_3_O_2_RuS requires 428.0188; C_12_H_19_F_9_N_3_O_2_PRuS (%): calcd C 25.18 H 3.35 N 7.34 S 5.60 found C 25.00 H 3.35 N 7.45 S 5.32.

**Complex 5:** [Ru(η^6^-1,1,1-trifluoro-*N*-(4-phenylbutyl)methanesulfonamide)Cl_2_]_2_ (127 mg, 0.140 mmol) was dissolved in MeOH (20 mL) to yield a red solution followed by the addition of ethylenediamine (28 µL, 0.419 mmol) to immediately yield a yellow solution. After stirring for 1 h NH_4_PF_6_ (137 mg, 0.840 mmol) was added to the solution to yield an immediate precipitate—the suspension was stirred for 10 min then filtered, concentrated then left to vapor diffuse with diethyl ether. Over 48 h a dark residue precipitated—the solvent was decanted and left to vapor diffuse with diethyl ether over 72 h to yield orange crystals that were isolated and dried under reduced pressure. (53 mg, 0.085 mmol, 30%). ^1^H NMR (DMSO-*d*_6_, 400 MHz): *δ* = 9.40 (br, 1H, N*H*), 6.41 (br, 2H, 2 × en N*H*), 5.71 (dd, 2H, *J* = 5.5, 6.0 Hz, Ar C*H*), 5.53 (t, 1H, *J* = 5.0 Hz, Ar C*H*), 5.50 (d, 2H, *J* = 6.0 Hz, Ar C*H*), 4.14 (br, 2H, 2 × en N*H*), 3.18 (t, 2H, *J* = 7.0 Hz, -C*H*_2_NH-), 2.42 (t, 2H, *J* = 7.0 Hz, -C*H*_2_Ar), 2.14–2.36 (m, 4H, NH_2_C*H*_2_C*H*_2_NH_2_), 1.57 (m, 4H, -CH_2_-C*H*_2_-C*H*_2_-CH_2_-); ^19^F (DMSO-*d*_6_, 376.5 MHz): *δ* = −70.00 (d, 6F, *J* = 715 Hz, PF_6_^−^), −77.26 (s, 3F, C*F*_3_-); ^13^C{^1^H} NMR (DMSO-*d*_6_, 100 MHz): *δ* = 119.7 (q, J = 321 Hz), 102.2, 83.4, 81.2, 78.7, 44.3, 43.1, 31.7, 29.3, 26.3; HRMS (ES^+^) *m*/*z* found 478.0184 [M]^+^ C_13_H_22_ClF_3_N_3_O_2_RuS requires 478.0111; C_13_H_22_ClF_9_N_3_O_2_PRuS (%): calcd C 25.07 H 3.56 N 6.75 S 5.15 found C 25.26 H 3.43 N 6.87 S 5.00.

**Complex 6:** [Ru(η^6^-*N*-phenethylacetamide)Cl_2_]_2_ (96 mg, 0.143 mmol) was suspended in methanol (30 mL) followed by the addition of ethylenediamine (19 µL, 0.284 mmol). This resulted in the solubilization of the bulk of the reaction mixture to form a straw-colored solution. After stirring for 1 h the reaction mixture was filtered followed by the addition of NH_4_PF_6_ (139 mg, 0.853 mmol)–the mixture was stirred for 20 min then filtered and dried under reduced pressure. The yellow residue was resuspended in MeOH (8 mL), filtered then the volume of the solution was concentrated under reduced pressure (~2 mL) and left to cool at −18 °C. Crystals that formed over 5 days were collected, washed with EtOH (0.5 mL) then recrystallized by dissolution in hot EtOH (4 mL), filtering then cooling at −18 °C. After 24 h the solution was decanted from the solid which had formed, the solid washed with EtOH (1 mL) then dried under reduced pressure. The solid was then dissolved in water and lyophilized to remove residual solvent to leave the product as a yellow orange powder (40 mg, 0.079 mmol, 28%). ^1^H NMR (DMSO-*d*_6_, 400 MHz): *δ* = 7.98 (t, 1H, *J* = 5.5 Hz, N*H*), 6.42 (br, 2H, 2 × en N*H*), 5.70 (dd, 2H, *J* = 6.0, 6.0 Hz, 2 × Ar C*H*), 5.53 (t, 1H, *J* = 5.0 Hz, 1 × Ar C*H*), 5.50 (d, 2H, *J* = 5.5 Hz, 2 × Ar C*H*), 4.13 (br, 2H, 2 × en N*H*), 3.30 (m, 2H, -C*H*_2_NH-), 2.54 (t, 2H, *J* = 7.0 Hz, -C*H*_2_Ar), 2.15-2.36 (m, 4H, NH_2_C*H*_2_C*H*_2_NH_2_), 1.78 (s, 3H, -C*H*_3_); ^19^F (DMSO-*d*_6_, 376.5 MHz): *δ* = −70.02 (d, 6F, *J* = 711 Hz, PF_6_^−^); ^13^C{^1^H} NMR (DMSO-*d*_6_, 100 MHz): *δ* = 169.2, 99.7, 83.0, 81.9, 79.0, 44.3, 38.7, 32.7, 22.6; HRMS (ES^+^) *m*/*z* found 360.0417 [M]^+^ C_12_H_21_ClN_3_ORu requires 360.0411; C_12_H_21_ClF_6_N_3_OPRu (%): calcd C 28.55 H 4.19 N 8.32 found C 28.42 H 4.06 N 8.15.

**[Ru(η^6^-C_6_H_5_CH_2_CH_2_NHTr)(C_2_O_4_)(H_2_O)]:** [Ru(η^6^-2,2,2-trifluoro-*N*-phenethylethane-1-sulfonamide)Cl_2_]_2_ (211 mg, 0.240 mmol) was suspended in H_2_O (20 mL) followed by the addition of silver oxalate (218 mg, 0.718 mmol). The suspension was stirred in the dark for 2 h, allowed to settle then filtered through a Millex 0.20 µm PTFE syringe filter tip. The liquid was then concentrated under reduced pressure at 45 °C to 5 mL then the resulting aqueous solution was lyophilized to leave the desired product as a yellow powder (170 mg, 0.358 mmol, 75%). ^1^H NMR (D_2_O, 400 MHz): *δ* = 5.97 (dd, 2H, *J* = 6.5, 5.5 Hz, Ar C*H*), 5.83 (t, 1H, *J* = 3.0 Hz, Ar C*H*), 5.71 (d, 2H, *J* = 6.0 Hz, Ar C*H*), 4.25 (q, 2H, *J* = 8.5 Hz, -C*H*_2_CF_3_), 3.53 (t, 2H, *J* = 6.5 Hz, -C*H*_2_NH-), 2.78 (t, 2H, *J* = 5.0 Hz, -C*H*_2_CH_2_NH-); ^19^F (D_2_O, 376.5 MHz): *δ* = −62.71 (t, 3F, *J* = 9.0 Hz, C*F*_3_CH_2_-); ^13^C{^1^H} NMR (D_2_O, 100 MHz): *δ* = 166.3, 121.7 (q, *J* = 275 Hz), 96.4, 83.2, 77.5, 77.3, 53.0 (q, *J* = 31.5 Hz), 41.8, 33.1; HRMS (ES^+^) *m*/*z* found 457.9474 [M − H_2_O + H]^+^ C_12_H_13_F_3_NO_6_RuS requires 457.9456; C_12_H_14_F_3_NO_7_RuS (%): calcd C 30.38 H 2.97 N 2.95 S 6.76 found C 30.21 H 2.71 N 3.17 S 6.53.

### 4.4. NMR Studies

pH titrations of **1**–**6** and [Ru(η^6^-C_6_H_5_CH_2_CH_2_NHTr)(C_2_O_4_)(H_2_O)] were conducted by adjustment of the pD of solutions of the complexes (2.46 mM, 295 K, 0.1 M NaCl) in D_2_O through addition of 1–5 µL aliquots of NaOD (3.6 wt.%) and DCl (1.8 wt.%) solutions in D2O. Following pD adjustment the solutions were left to equilibrate (for **1**–**6** this was up to 36 h) then the ^1^H and ^19^F spectra of each were recorded. The ratio of selected ligand signals (for example –CH_3_ for **1**, arene resonances for **2**, –CF_3_ for **3**–**5**) corresponding to the open- and chelate-forms of the complex were determined for each pD value and enabled a plot of % open-form versus pD. p*K*_a_ values were calculated through fitting of the Henderson-Hasselbalch equation to the data.

The reactivity of **4** and **6** towards 5′-GMP/L-histidine was assessed by mixing stock solutions of the complex and ligand in phosphate buffer (both in D_2_O, 0.1 M NaCl) to yield 1:1 solutions with each component present at a concentration of 2.46 mM (200 mM buffer concentration, 0.1 M NaCl). These studies were performed using phosphate buffers at pD values of 7.5 and 6.5. Samples were incubated at 310 K and monitored using ^1^H and ^19^F NMR. The reactivity of **4** towards bovine serum albumin was assessed by mixing stock solutions of **4** and protein in phosphate buffer (in D_2_O, 0.1 M NaCl) to yield a mixture of **4** and protein at 2.45 mM and 0.5 mM respectively (200 mM buffer concentration, 0.1 M NaCl). The pD of the final solution was 7.4, it was incubated at 310 K and monitored using ^1^H and ^19^F NMR.

The conversion of the chelate-form of **4** to the activated open-form was investigated by dilution of a stock solution of **4** (D_2_O, 0.1 M NaCl) into citrate/phosphate buffer solutions (D_2_O, 0.1 M NaCl) at pD 4.12, 4.91 and 5.96 to yield solutions at a final complex concentration of 2.46 mM. The samples were maintained at 310 K within the NMR spectrometer and ^19^F NMR spectra were recorded every 15 min for 16 h. The ratio of the –CF_3_ signals corresponding to the open- and chelate-forms of the complex were determined to allow a plot of % open-form versus time. The fitting of the equation y = C + A(1−e^−kx^) (*C*-offset from zero; A-amplitude of the curve) to each set of experimental data yielded the half-life for the fraction of the chelate-form of **4** which is converted to the open-form.

### 4.5. Cell Culture

Protocol for cytotoxicity experiments with **4** and **6** and HT-29 cells (The European Collection of Authenticated Cell Cultures, Salisbury, UK).

Preparation of the stock solutions:

**4** and **6** were weighed into 12 × 75 mm polystyrene tubes. Each ruthenium compound was diluted in 1 mL of H_2_O and the solutions were vortexed then filtered through a 0.22 µm syringe filter to sterilize them.

Cytotoxicity experiments:

HT-29 (human colon adenocarcinoma) cells were counted and the concentration adjusted to 3 × 10^4^ cells/.mL 100 µL of the cell suspension was dispensed into the wells of a 96 well plate. The cells were left to attach for 24 h after which the original medium was removed and 100 µL of the dilutions of **4** or **6** (or new medium for cells only control) was added. The plates were incubated at 37 °C and 5% CO_2_ for 72 h. After this incubation period 10µL of MTT solution (3-[4,5-dimethylthiazol-2-yl]-2,5-diphenyltetrazolium bromide-Thiazolyl blue; Sigma M5655 made up in PBS and filtered through a 0.22 µm syringe filter, Gillingham, UK) was added to each well. The color was allowed to develop for 3 h after which the reaction was halted by adding 150µL of acid-alcohol (0.04 M HCl in isopropanol) to each well. The results were read on a plate reader (Biotek ELX800 Universal Microplate Reader) at 570 nm. The viability of the cells is expressed as % of cell survival of each dilution against the “cells only” control taken as 100% survival. The media used in these assays contained either 1% or 10% fetal calf serum.

## Figures and Tables

**Table 1:** Apparent p*K*_a_ values determined for **1**–**5** and their [Ru(η^6^-C_6_H_5_CH_2_CH_2_NHR)(C_2_O_4_)(H_2_O)] analogues.

Complex	Apparent p*K*_a_	Apparent p*K*_a_ for the Analogous [Ru(η^6^-C_6_H_5_CH_2_CH_2_NHR)(C_2_O_4_)(H_2_O)] Complex
**1**	7.32	6.65 ^‡^
**2**	6.41	5.75
**3**	4.50	4.05 ^‡^
**4**	4.82	-
**5**	8.00	-

^‡^ Values for [Ru(η^6^-C_6_H_5_CH_2_CH_2_NHR)(C_2_O_4_)(H_2_O)] (R = Ms, Tf) have been reported previously [15].

## Supplementary Materials

The following are available online. A figure illustrating disorder in **1** and NMR Spectra (Figures S1–S22), X-ray diffraction parameters (Tables S1–S2). CCDC 1963883, 1963884, 1963885, 1963886, 1963887, 1963888 and 1963889 contain the supplementary crystallographic data presented in this paper. These data can be obtained free of charge from The Cambridge Crystallographic Data Centre via www.ccdc.cam.ac.uk/structures.

## Appendix A

For **5** only a partial data set is presented in Figure 7. This is due to a significant increase in complexity observed in the arene region of ^1^H NMR spectra at pD values greater than those of the data points presented in Figure 7. We believe the increase in complexity in the arene region is due to the formation of [Ru(η^6^-C_6_H_5_(CH_2_)_3_CH_2_NHTf)(en)(OH)]^+^—there is no increase in complexity in other regions of the spectrum. Due to overlapping peaks it was not possible to accurately quantify the different species present at the more basic pD values.

## References

[1] Casini A., Vessières A., Meier-Menches S.M. (2019). Metal-Based Anticancer Agents.

[2] Zhang P., Sadler P.J. (2017). Advances in the Design of Organometallic Anticancer Complexes. J. Organomet. Chem..

[3] Murray B.S., Babak M.V., Hartinger C.G., Dyson P.J. (2016). The development of RAPTA compounds for the treatment of tumors. Coord. Chem. Rev..

[4] Murray B.S., Dyson P.J. (2020). Recent Progress in the Development of Organometallics for the Treatment of Cancer. Curr. Opin. Chem. Biol..

[5] Estrella V., Chen T., Lloyd M., Wojtkowiak J., Cornnell H.H., Ibrahim-Hashim A., Bailey K., Balagurunathan Y., Rothberg J.M., Sloane B.F. (2013). Acidity Generated by the Tumor Microenvironment Drives Local Invasion. Cancer Res..

[6] Habtemariam A., Watchman B., Potter B.S., Palmer R., Parsons S., Parkin A., Sadler P.J. (2001). Control of Aminophosphine Chelate Ring-Opening in Pt(II) and Pd(II) Complexes: Potential Dual-Mode Anticancer Agents. Dalton Trans..

[7] Habtemariam A., Sadler P.J. (1996). Design of Chelate Ring-Opening Platinum Anticancer Complexes: Reversible Binding to Guanine. Chem. Commun..

[8] Friebolin W., Schilling G., Zöller M., Amtmann E. (2004). Synthesis and Structure−Activity Relationship of Novel Antitumoral Platinum Xanthate Complexes. J. Med. Chem..

[9] Galanski M., Baumgartner C., Meelich K., Arion V.B., Fremuth M., Jakupec M.A., Schluga P., Hartinger C.G., Graf. v. Keyserlingk N., Keppler B.K. (2004). Synthesis, Crystal Structure and pH Dependent Cytotoxicity of (*SP*-4-2)-bis(2-aminoethanolato-κ^2^
*N*,*O*)platinum(II)—A Representative of Novel pH Sensitive Anticancer Platinum Complexes. Inorg. Chim. Acta.

[10] Zorbas-Seifried S., Hartinger C.G., Meelich K., Galanski M., Keppler B.K., Zorbas H. (2006). DNA Interactions of pH-Sensitive, Antitumor Bis(aminoalcohol)dichloroplatinum(II) Complexes. Biochemistry.

[11] Scaffidi-Domianello Y.Y., Legin A.A., Jakupec M.A., Arion V.B., Kukushkin V.Y., Galanski M., Keppler B.K. (2011). Synthesis, Characterization, and Cytotoxic Activity of Novel Potentially pH-Sensitive Nonclassical Platinum(II) Complexes Featuring 1,3-Dihydroxyacetone Oxime Ligands. Inorg. Chem..

[12] Scrase T.G., O’Neill M.J., Peel A.J., Senior P.W., Matthews P.D., Shi H., Boss S.R., Barker P.D. (2015). Selective Lability of Ruthenium(II) Arene Amino Acid Complexes. Inorg. Chem..

[13] Martínez-Peña F., Pizarro A.M. (2017). Control of Reversible Activation Dynamics of [Ru{η^6^:κ^1^--C_6_H_5_(C_6_H_4_)NH_2_}(XY)]^n+^ and the Effect of Chelating-Ligand Variation. Chem. Eur. J..

[14] Martínez-Peña F., Infante-Tadeo S., Habtemariam A., Pizarro A.M. (2018). Reversible pH-Responsive Behavior of Ruthenium(II) Arene Complexes with Tethered Carboxylate. Inorg. Chem..

[15] Prior T.J., Savoie H., Boyle R.W., Murray B.S. (2018). pH-Dependent Modulation of Reactivity in Ruthenium(II) Organometallics. Organometallics.

[16] Morris R.E., Aird R.E., del Socorro Murdoch P., Chen H., Cummings J., Hughes N.D., Parsons S., Parkin A., Boyd G., Jodrell D.I. (2001). Inhibition of Cancer Cell Growth by Ruthenium(II) Arene Complexes. J. Med. Chem..

[17] Habtemariam A., Melchart M., Fernández R., Parsons S., Oswald I.D.H., Parkin A., Fabbiani F.P.A., Davidson J.E., Dawson A., Aird R.E. (2006). Structure-Activity Relationships for Cytotoxic Ruthenium(II) Arene Complexes Containing N,N-, N,O-, and O,O-Chelating Ligands. J. Med. Chem..

[18] Bernstein J., Davis R.E., Shimoni L., Chang N.-L. (1995). Patterns in Hydrogen Bonding: Functionality and Graph Set Analysis in Crystals. Angew. Chem. Int. Ed..

[19] Skjærvø S.L., Høydalsvik K., Blichfeld A.B., Einarsrudand M.-A., Grande T. (2018). Thermal Evolution of the Crystal Structure and Phase Transitions of KNbO_3_. R. Soc. Open Sci..

[20] Glasoe P.K., Long F.A.J. (1960). Use of Glass Electrodes to Measure Acidities in Deuteium Oxide. Phys. Chem..

[21] Bradbury J.H., Chapman B.E., Pellegrino F.A. (1973). Hydrogen-Deuterium Exchange Kinetics of the C-2 Protons of Imidazole and Histidine Compounds. J. Am. Chem. Soc..

[22] Canel E., Gültepe A., Doğan A., Kiliç E. (2006). The Determination of Protonation Constants of Some Amino Acids and Their Esters by Potentiometry in Different Media. J. Soln. Chem..

[23] Chen H., Parkinson J.A., Morris R.E., Sadler P.J. (2003). Highly Selective Binding of Organometallic Ruthenium Ethylenediamine Complexes to Nucleic Acids:  Novel Recognition Mechanisms. J. Am. Chem. Soc..

[24] Blessing R. (1995). An Empirical Correction for Absorption Anisotropy. Acta Cryst..

[25] Sheldrick G.M. (2015). SHELXT - Integrated Space-Group and Crystal-Structure Determination. Acta Cryst..

[26] Sheldrick G.M. (2015). Crystal Structure Refinement With SHELXL. Acta Cryst..

[27] Ito M., Endo Y., Ikariya T. (2008). Well-Defined Triflylamide-Tethered Arene−Ru(Tsdpen) Complexes for Catalytic Asymmetric Hydrogenation of Ketones. Organometallics.

[28] Reiner T., Jantke D., Miao X.-H., Marziale A.N., Kiefer F.J., Eppinger J. (2013). Phenylalanine—A Biogenic Ligand with Flexible η^6^- and η^6^:κ^1^-Coordination at Ruthenium(II) Centres. Dalton Trans..

[29] Crabb T.A., Wilkinson J.R. (1975). Synthesis of Hexahydroisoquinolines. J. Chem. Soc. Perkin Trans..

