# Supplementary material for: The Application of Reversible Intramolecular Sulfonamide Ligation to Modulate Reactivity in Organometallic Ruthenium(II) Diamine Complexes
Source: Molecules. 2020 Jan 7;25(2):244. doi: 10.3390/molecules25020244 (PMC7024221; doi:10.3390/molecules25020244)
Supplement: Supplementary file 1 [file molecules-25-00244-s001.pdf]

The Application of Reversible Intramolecular Sulfonamide Ligation to Modulate Reactivity in Organometallic Ruthenium(II) Diamine Complexes.

Samuel Kemp, Timothy J. Prior, Huguette Savoie, Ross W. Boyle, Benjamin S. Murray*
Department of Chemistry & Biochemistry, University of Hull, Hull HU6 7RX, UK. E-mail b.s.murray@hull.ac.uk.

Crystallography

Figure S1: Disorder present in **1**. The second component generated by the action of the mirror plane is shown in blue.

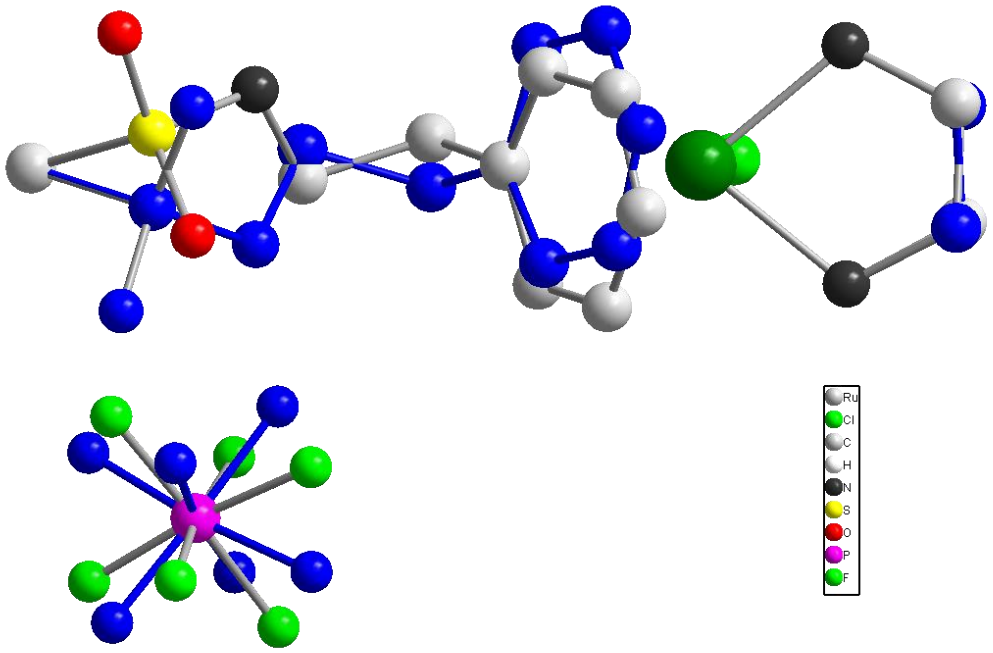

Table S1: X-ray diffraction parameters for the measurement of single crystals of **1** – **6**.

**Identification code**	**1**	**2**	**3**	**4**	**5**	**6**
Empirical formula	C11 H21 Cl F6 N3 O2 P Ru S	C12 H20 Cl F9 N3 O2 P Ru S	C11 H18 Cl F9 N3 O2 P Ru S	C12 H15 F9 N3 O2 P Ru S	C13 H22 Cl F9 N3 O2 P Ru S	C12 H21 Cl F6 N3 O P Ru
Formula weight	540.86	608.86	594.98	568.37	622.88	504.81
Temperature / K	150(2)	150(2)	150(2)	150(2)	290(2)	100(2)
Wavelength / Å	0.71073	0.71073	0.71073	0.71073	0.71073	0.71073
Crystal system	Monoclinic	Triclinic	Monoclinic	Monoclinic	Monoclinic	Monoclinic
Space group	P 2_1/m	P-1	P2_1/c	P2_1/n	P2_1/c	Cc
Unit cell dimensions	a = 9.797(3) Å, α= 90°.<br>b = 8.0578(14) Å, β= 106.65(2)°.<br>c = 12.566(3) Å, γ = 90°.	a = 8.653(2) Å, α= 62.25(2)°.<br>b = 11.646(4) Å, β= 74.794(17)°.<br>c = 12.582(2) Å, γ = 70.94(2)°.	a = 10.2766(6) Å, α= 90°.<br>b = 15.9431(11) Å, β= 103.692(5)°.<br>c = 12.2666(7) Å, γ = 90°.	a = 17.3745(6) Å, α= 90°.<br>b = 12.9936(6) Å, β= 106.252(3)°.<br>c = 17.5162(6) Å, γ = 90°.	a = 10.6575(10) Å, α= 90°.<br>b = 18.2064(11) Å, β= 102.291(7)°.<br>c = 11.8695(11) Å, γ = 90°.	a = 21.3588(13) Å, α= 90°.<br>b = 11.3132(5) Å, β= 98.312(5)°.<br>c = 15.2874(9) Å, γ = 90°.
Volume / $Å^3$	950.4(4)	1051.3(5)	1952.7(2)	3796.4(3)	2250.3(3)	3655.2(4)
Z	2	2	4	8	4	8
Density (calculated) / Mgm^{-3}	1.890	1.923	2.024	1.989	1.839	1.835
Absorption coefficient / mm^{-1}	1.227	1.141	1.226	1.120	1.068	1.155
F(000)	540	604	1176	2240	1240	2016
Crystal size / mm^3	0.310 × 0.280 × 0.050	0.350 × 0.180 × 0.030	0.140 × 0.140 × 0.130	0.5 × 0.4 × 0.28	0.480 x 0.450 x 0.440	0.250 x 0.220 x 0.180
2θ range for data collection / °	2.170 to 26.367	1.845 to 26.372	2.040 to 29.253	1.945 to 29.201	2.082 to 27.999	3.267 to 33.432
Index ranges	-12 ≤ h ≤ 12, -9 ≤ k ≤ 10,<br>-13 ≤ l ≤ 15	-10 ≤ h ≤ 10, -14 ≤ k ≤ 14,<br>-12 ≤ l ≤ 15	-11 ≤ h ≤ 14, -21 ≤ k ≤ 19,<br>-16 ≤ l ≤ 16	-23 ≤ h ≤ 22, -16 ≤ k ≤ 17,<br>-23 ≤ l ≤ 23	-10 ≤ h ≤ 14, -23 ≤ k ≤ 24,<br>-15 ≤ l ≤ 15	-33 ≤ h ≤ 29, -14 ≤ k ≤ 17,<br>-22 ≤ l ≤ 23
Reflections collected	6550	14406	14660	29653	14540	15658
Independent reflections	2071 [R(int) = 0.0337]	14406 [R(int) = 0.120]	5232 [R(int) = 0.0331]	10212 [R(int) = 0.0296]	5424 [R(int) = 0.0545]	11230 [R(int) = 0.0335]
Completeness to theta = 25.242°	99.3 %	97.3 %	99.6 %	100.0 %	99.7 %	99.2 %
Absorption correction	Semi-empirical from equivalents	Analytical	Semi-empirical from equivalents	Semi-empirical from equivalents	Semi-empirical from equivalents	Semi-empirical from equivalents
Max. and min. transmission	0.776 and 0.733	0.9678 and 0.7151	0.968 and 0.963	0.933 and 0.915	0.773 and 0.721	0.839 and 0.787
Refinement method	Full-matrix least-squares on F^2	Full-matrix least-squares on F^2	Full-matrix least-squares on F^2	Full-matrix least-squares on F^2	Full-matrix least-squares on F^2	Full-matrix least-squares on F^2
Data / restraints / parameters	2071 / 248 / 195	14406 / 0 / 272	5232 / 15 / 267	10212 / 29 / 495	5424 / 49 / 262	11230 / 52 / 457
Goodness-of-fit on F^2	1.065	0.929	0.856	1.063	0.950	0.972
Final R indices [I>2sigma(I)]	R1 = 0.0610, wR2 = 0.1713	R1 = 0.1013, wR2 = 0.2558	R1 = 0.0290, wR2 = 0.0579	R1 = 0.0471, wR2 = 0.1380	R1 = 0.0601, wR2 = 0.1752	R1 = 0.0356, wR2 = 0.0857
R indices (all data)	R1 = 0.0768, wR2 = 0.1820	R1 = 0.1380, wR2 = 0.2807	R1 = 0.0504, wR2 = 0.0610	R1 = 0.0643, wR2 = 0.1445	R1 = 0.0915, wR2 = 0.1903	R1 = 0.0429, wR2 = 0.0882
Absolute structure parameter	none	none	none	none	none	0.36(3)
Largest diff. peak and hole / $eÅ^{-3}$	1.156 and -1.078	2.218 and -1.758	0.929 and -0.505	1.301 and -1.375	1.223 and -1.253	0.891 and -0.772

Table S2: Extra data table to compare **6** at different temperatures.

**Identification code**	**6 (low T)**	**6 (high T)**
Empirical formula	C12 H21 Cl F6 N3 O P Ru	C12 H21 Cl F6 N3 O P Ru
Formula weight	504.81	504.81
Temperature / K	100(2)	298(2)
Wavelength / Å	0.71073	0.71073
Crystal system	Monoclinic	Monoclinic
Space group	Cc	C2/c
Unit cell dimensions	a = 21.3588(13) Å, α= 90°.<br>b = 11.3132(5) Å, β= 98.312(5)°.<br>c = 15.2874(9) Å, γ = 90°.	a = 21.808(8) Å, α= 90°.<br>b = 11.359(3) Å, β= 98.91(3)°.<br>c = 15.552(7) Å, γ = 90°.
Volume / Å^3	3655.2(4)	3806(2)
Z	8	8
Density (calculated) / Mgm^{-3}	1.835	1.762
Absorption coefficient / mm^{-1}	1.155	1.110
F(000)	2016	2016
Crystal size / mm^3	0.250 x 0.220 x 0.180	0.300 x 0.250 x 0.180
2θ range for data collection / °	3.267 to 33.432	3.219 to 29.574
Index ranges	-33 ≤ h ≤ 29, -14 ≤ k ≤ 17,<br>-22 ≤ l ≤ 23	-30<=h<=30, -15<=k<=13, -<br>21<=l<=16
Reflections collected	15658	23219
Independent reflections	11230 [R(int) = 0.0335]	5313 [R(int) = 0.0442]
Completeness to theta = 25.242°	99.2 %	99.3 %
Absorption correction	Semi-empirical from equivalents	Semi-empirical from equivalents
Max. and min. transmission	0.839 and 0.787	0.842 and 0.711
Refinement method	Full-matrix least-squares on F^2	Full-matrix least-squares on F^2
Data / restraints / parameters	11230 / 52 / 457	5313 / 0 / 216
Goodness-of-fit on F^2	0.972	1.054
Final R indices [I>2sigma(I)]	R1 = 0.0356, wR2 = 0.0857	R1 = 0.0435, wR2 = 0.1198
R indices (all data)	R1 = 0.0429, wR2 = 0.0882	R1 = 0.0649, wR2 = 0.1293
Absolute structure parameter	0.36(3)	none
Largest diff. peak and hole / eÅ^{-3}	0.891 and -0.772	0.926 and -0.795

^{1}H, ^{13}C and ^{19}F NMR spectra

Figure S2: ^{1}H (top) and $^{13}C\{^{1}H\}$ and ^{19}F (following page) NMR spectra (DMSO-d_6) of **1**.

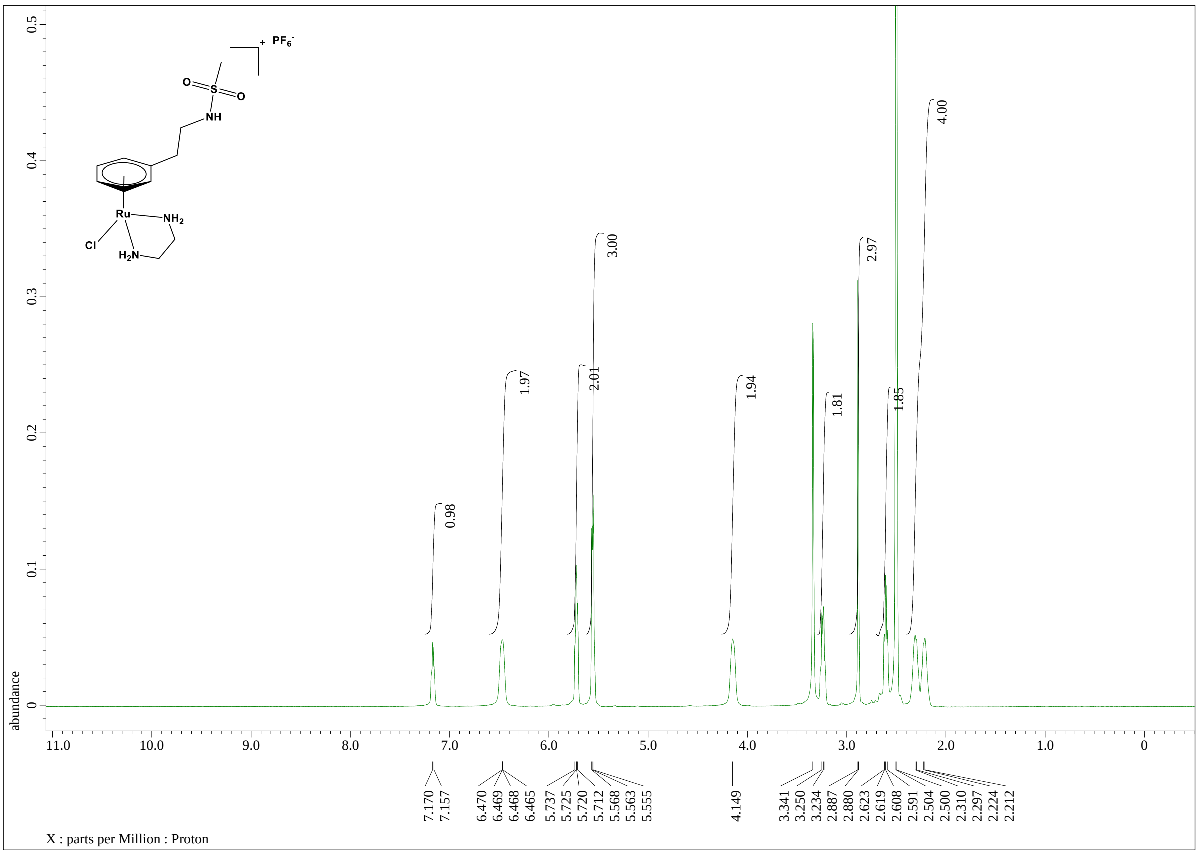

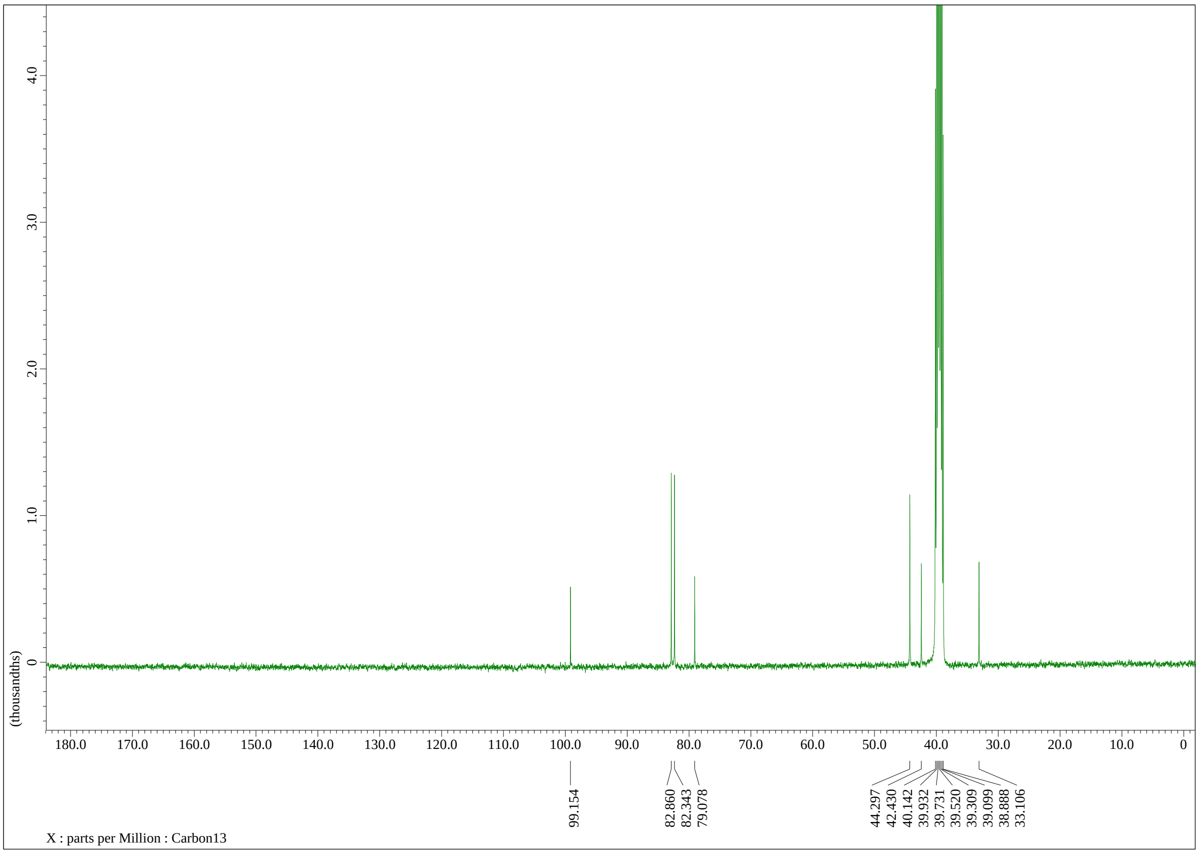

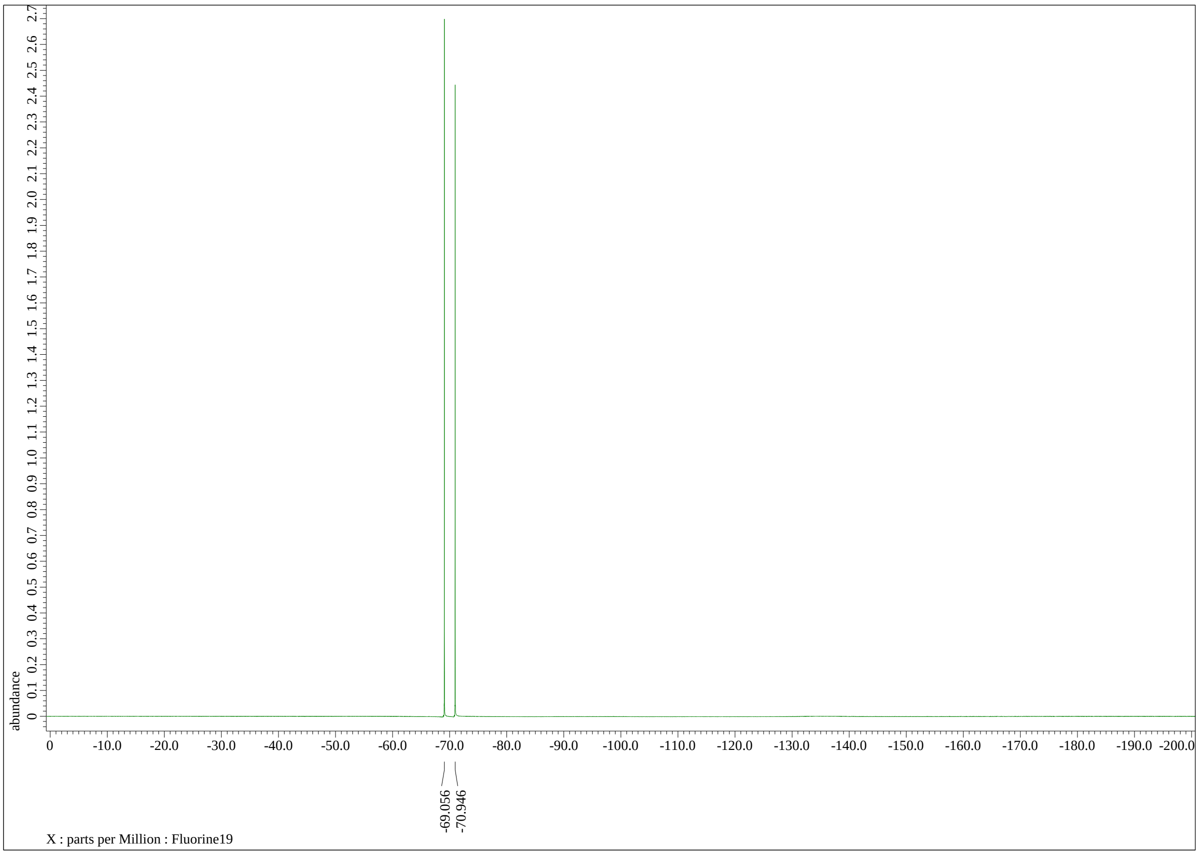
abundance
0
-10.0
-20.0
-30.0
-40.0
-50.0
-60.0
-70.0
-80.0
-90.0
-100.0
-110.0
-120.0
-130.0
-140.0
-150.0
-160.0
-170.0
-180.0
-190.0
-200.0
-69.056
-70.946
X : parts per Million : Fluorine19

Figure S3: ^{1}H (top), $^{13}C\{^{1}H\}$ and ^{19}F (following page) NMR spectra (DMSO-d_6) of ***N*-(2-(cyclohexa-1,4-dien-1-yl)ethyl)-2,2,2-trifluoroethane-1-sulfonamide**.

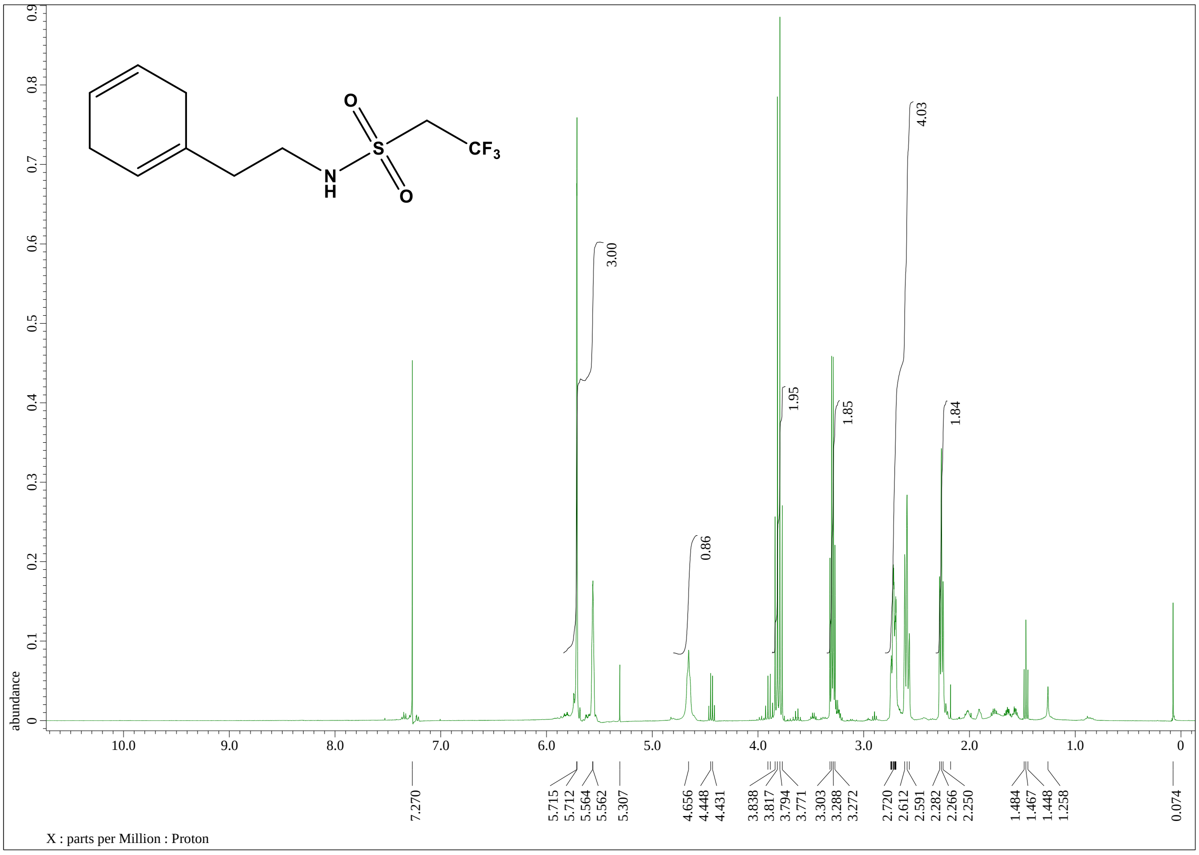

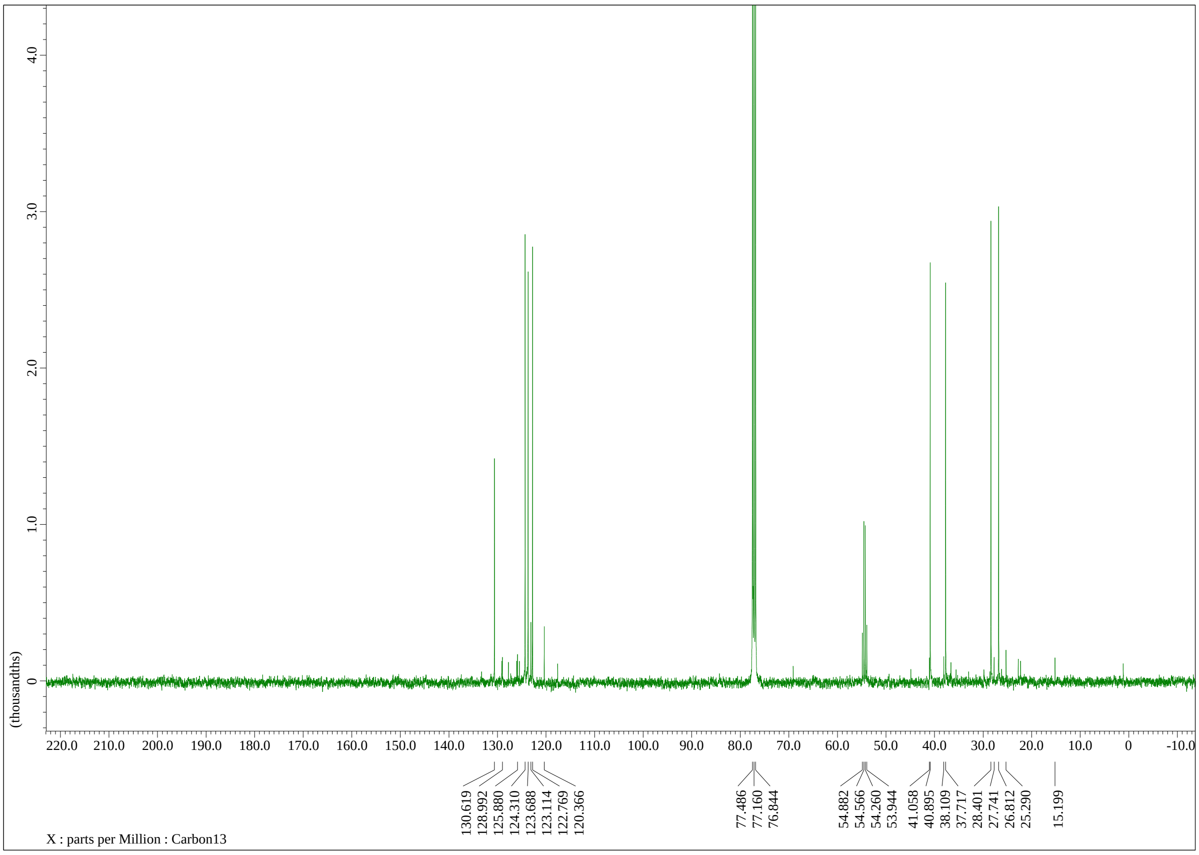

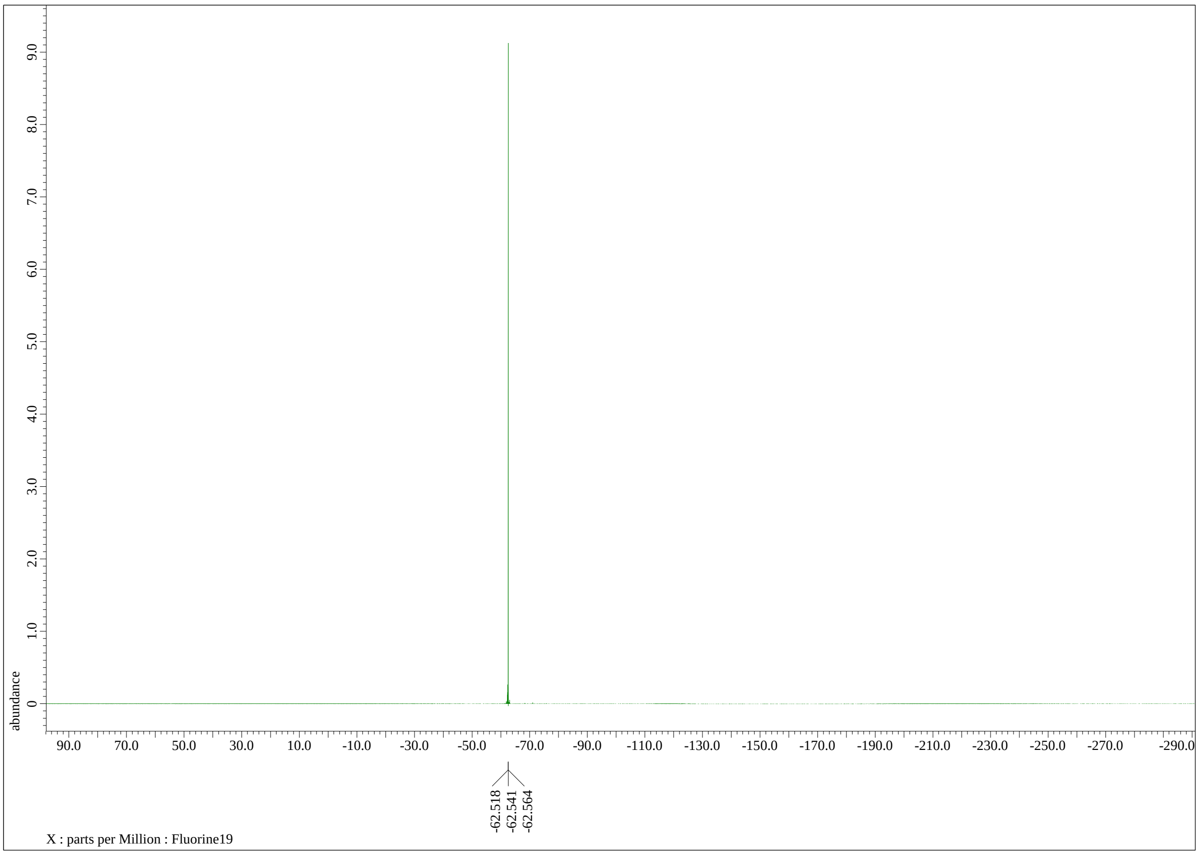

9.0
8.0
7.0
6.0
5.0
4.0
3.0
2.0
1.0
0
abundance
90.0
70.0
50.0
30.0
10.0
-10.0
-30.0
-50.0
-70.0
-90.0
-110.0
-130.0
-150.0
-170.0
-190.0
-210.0
-230.0
-250.0
-270.0
-290.0
-62.518
-62.541
-62.564
X : parts per Million : Fluorine19

Figure S4: ^{1}H (top), ^{13}C{^{1}H} and ^{19}F (following page) NMR spectra (DMSO-d_6) of **[Ru(η^{6}-2,2,2-trifluoro-*N*-phenethylethane-1-sulfonamide)Cl$_2$]$_2$**.

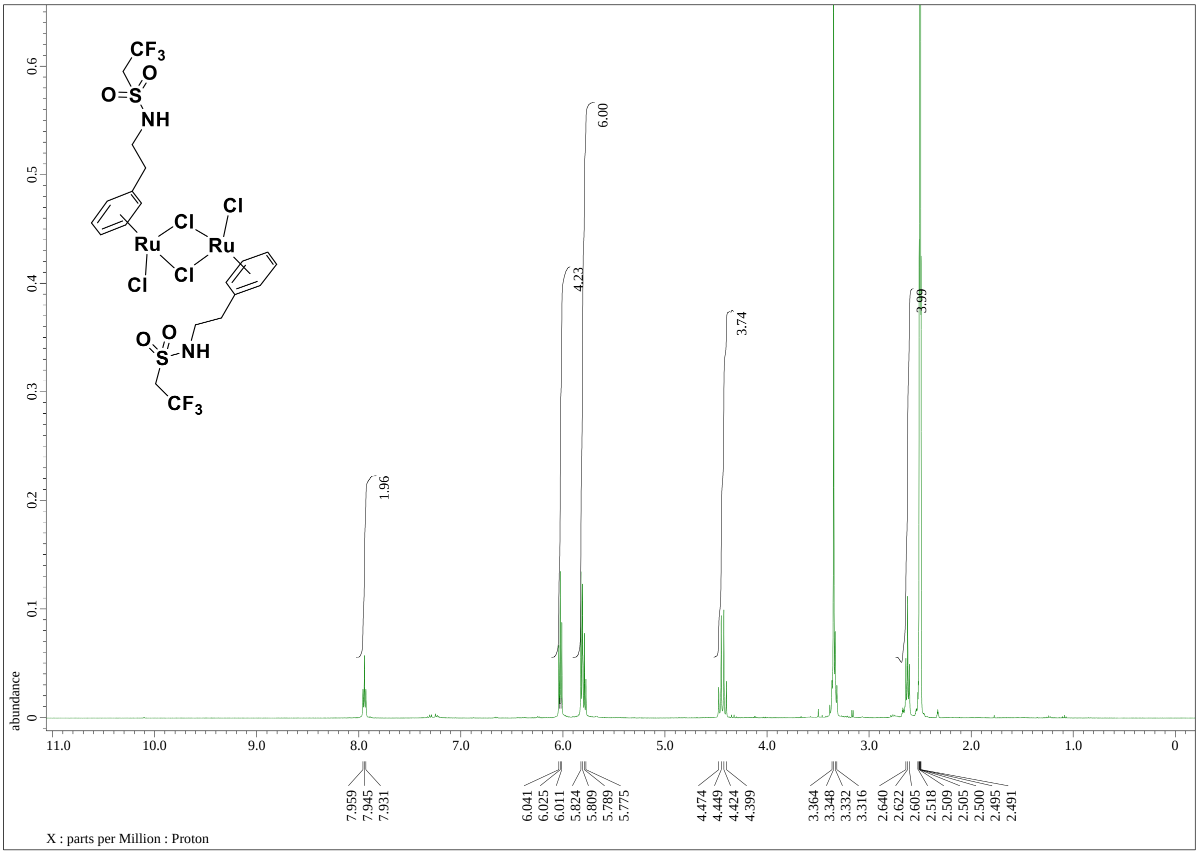

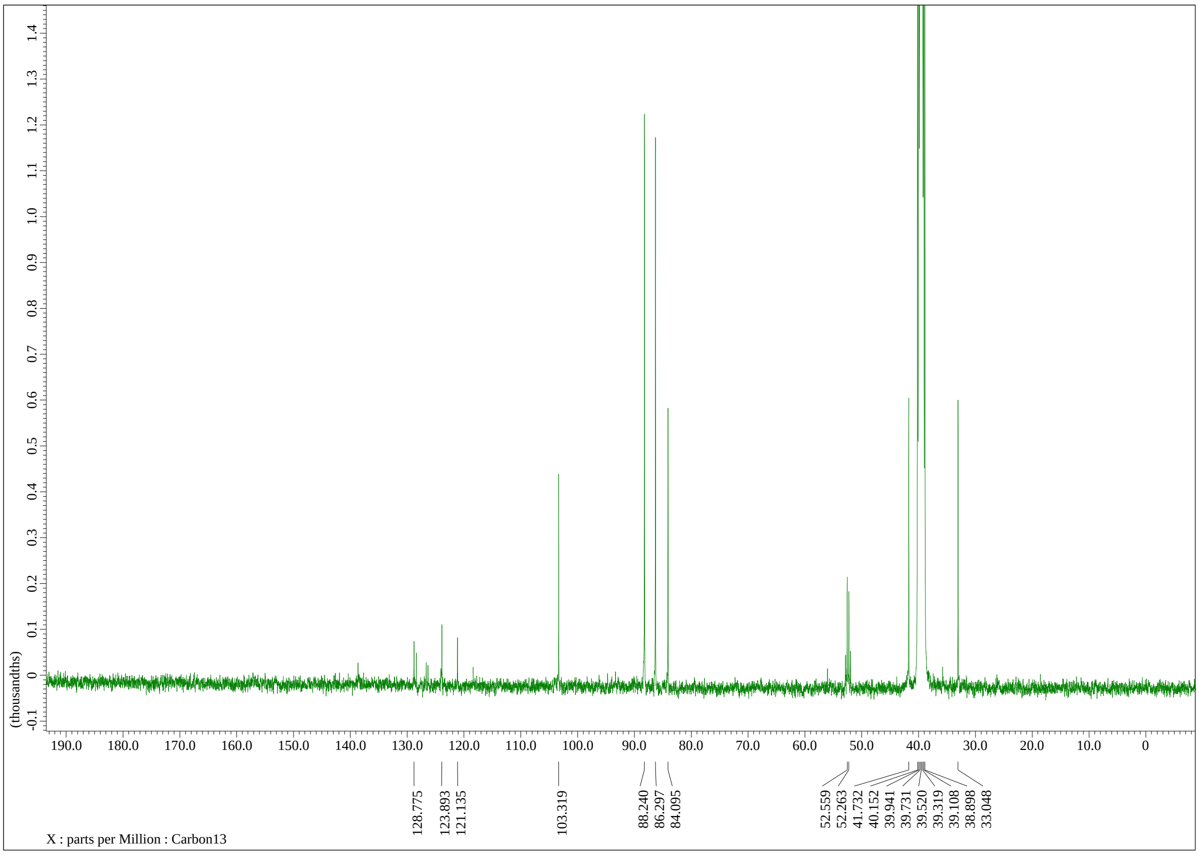

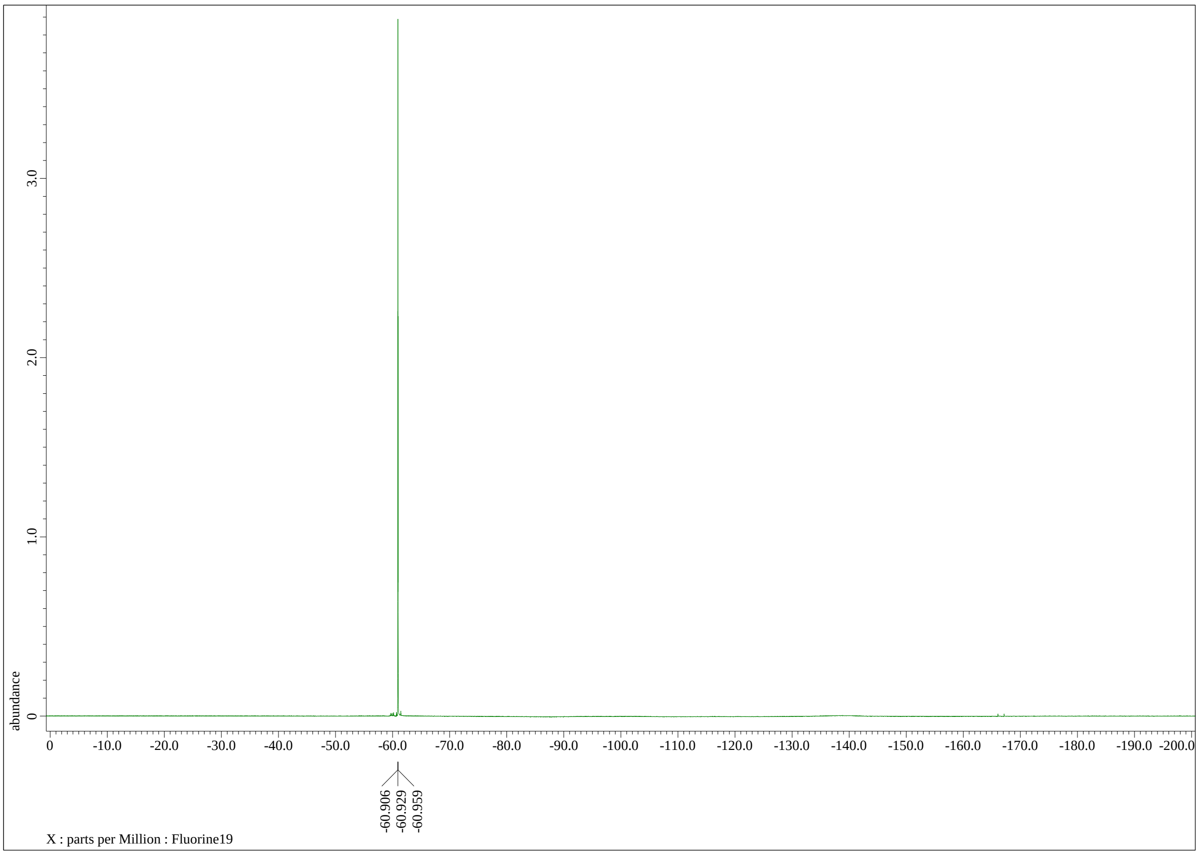

abundance
3.0
2.0
1.0
0
0
-10.0
-20.0
-30.0
-40.0
-50.0
-60.0
-70.0
-80.0
-90.0
-100.0
-110.0
-120.0
-130.0
-140.0
-150.0
-160.0
-170.0
-180.0
-190.0
-200.0
-60.906
-60.929
-60.959
X : parts per Million : Fluorine19

Figure S5: ^{1}H (top), $^{13}C\{^{1}H\}$ and ^{19}F (following page) NMR spectra (DMSO-d_6) of **2**.

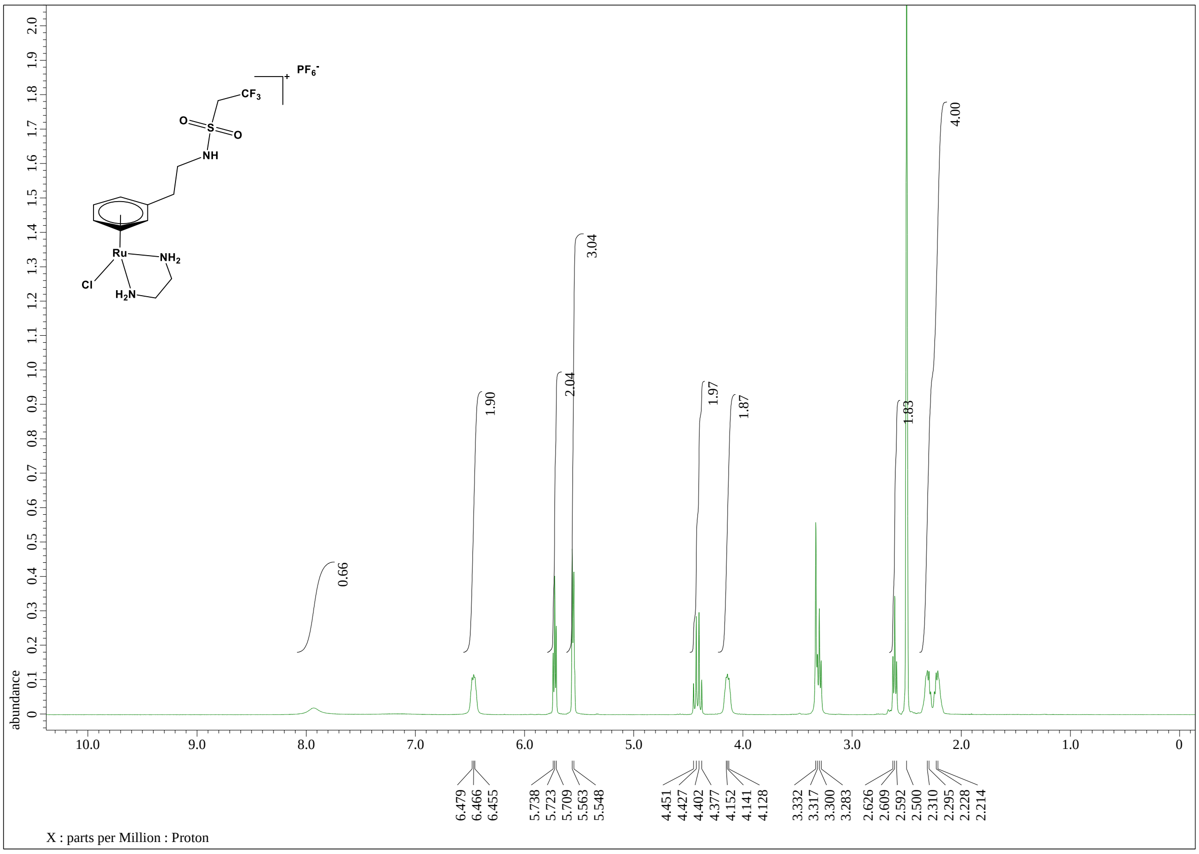

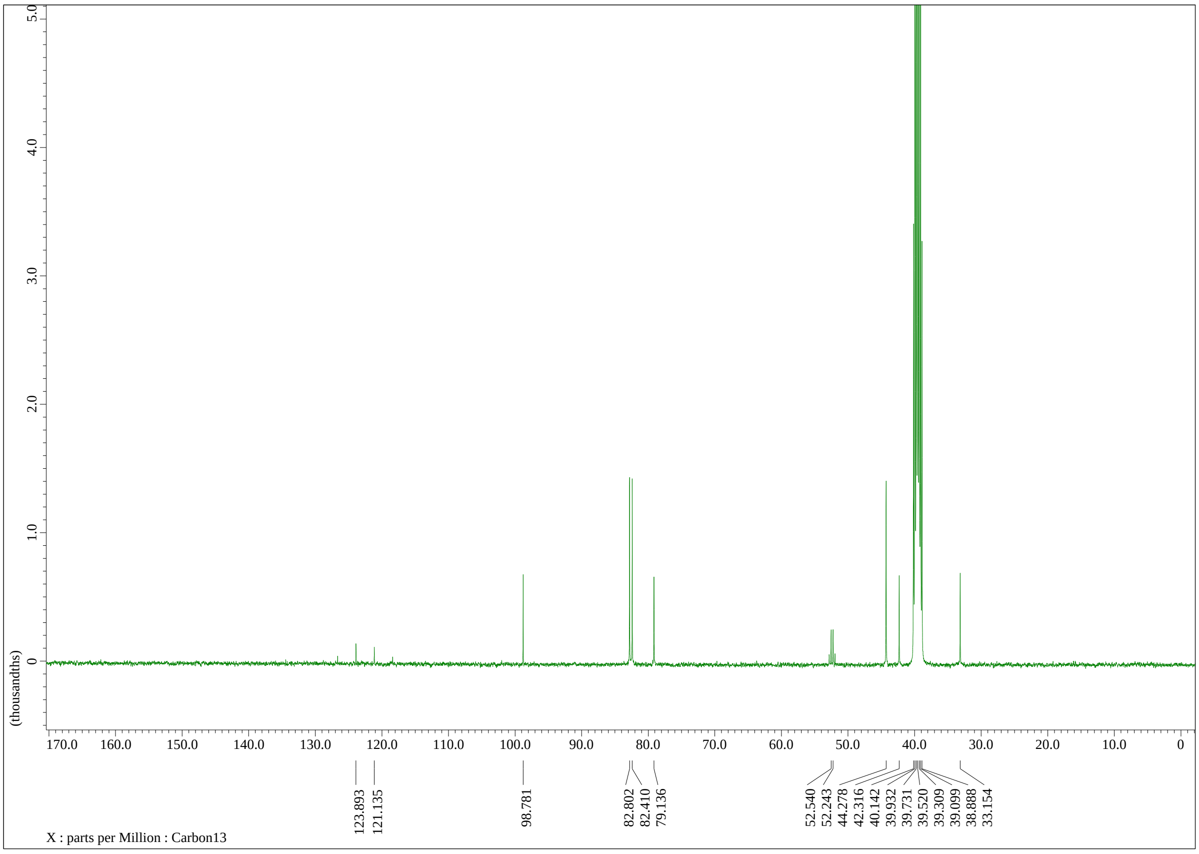

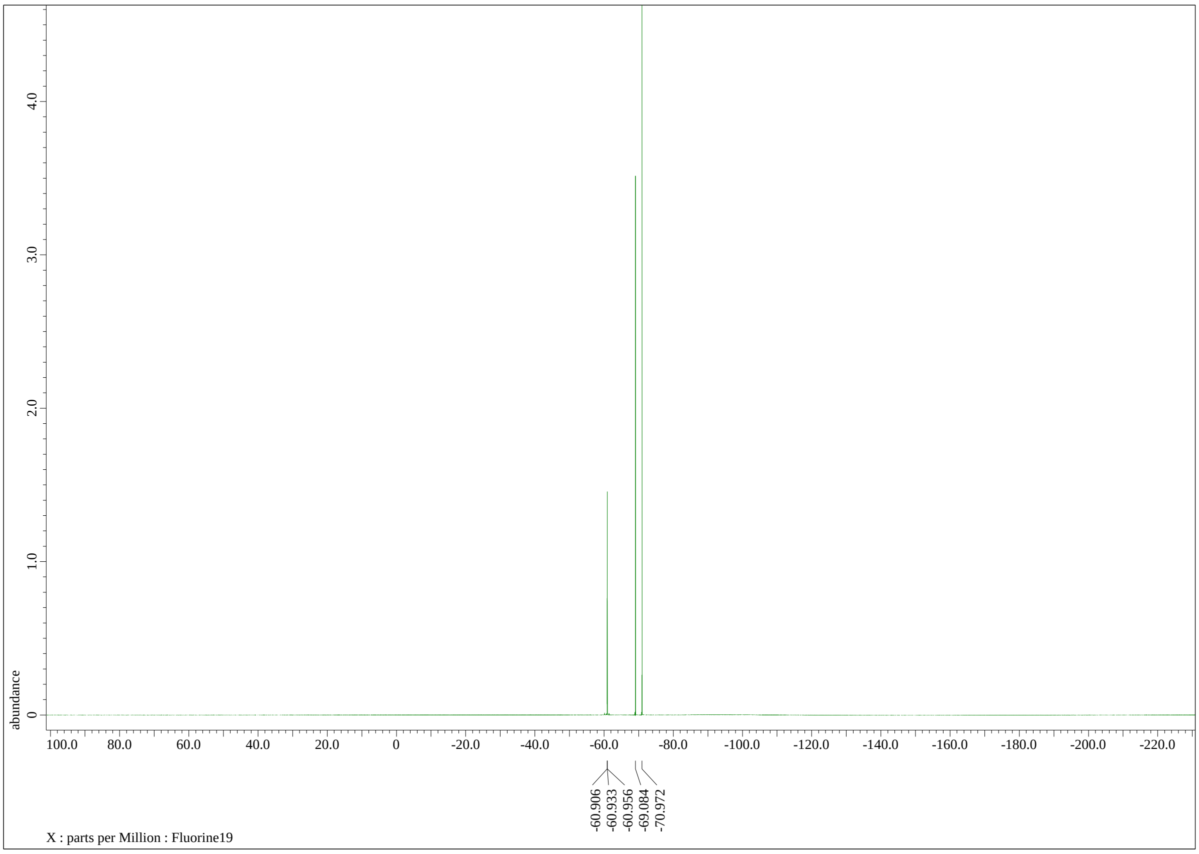

abundance
0
1.0
2.0
3.0
4.0
100.0
80.0
60.0
40.0
20.0
0
-20.0
-40.0
-60.0
-80.0
-100.0
-120.0
-140.0
-160.0
-180.0
-200.0
-220.0
-60.906
-60.933
-60.956
-69.084
-70.972
X : parts per Million : Fluorine19

Figure S6: ^{1}H (top), ^{13}C{^{1}H} and ^{19}F (following page) NMR spectra (DMSO-d_6) of **3**.

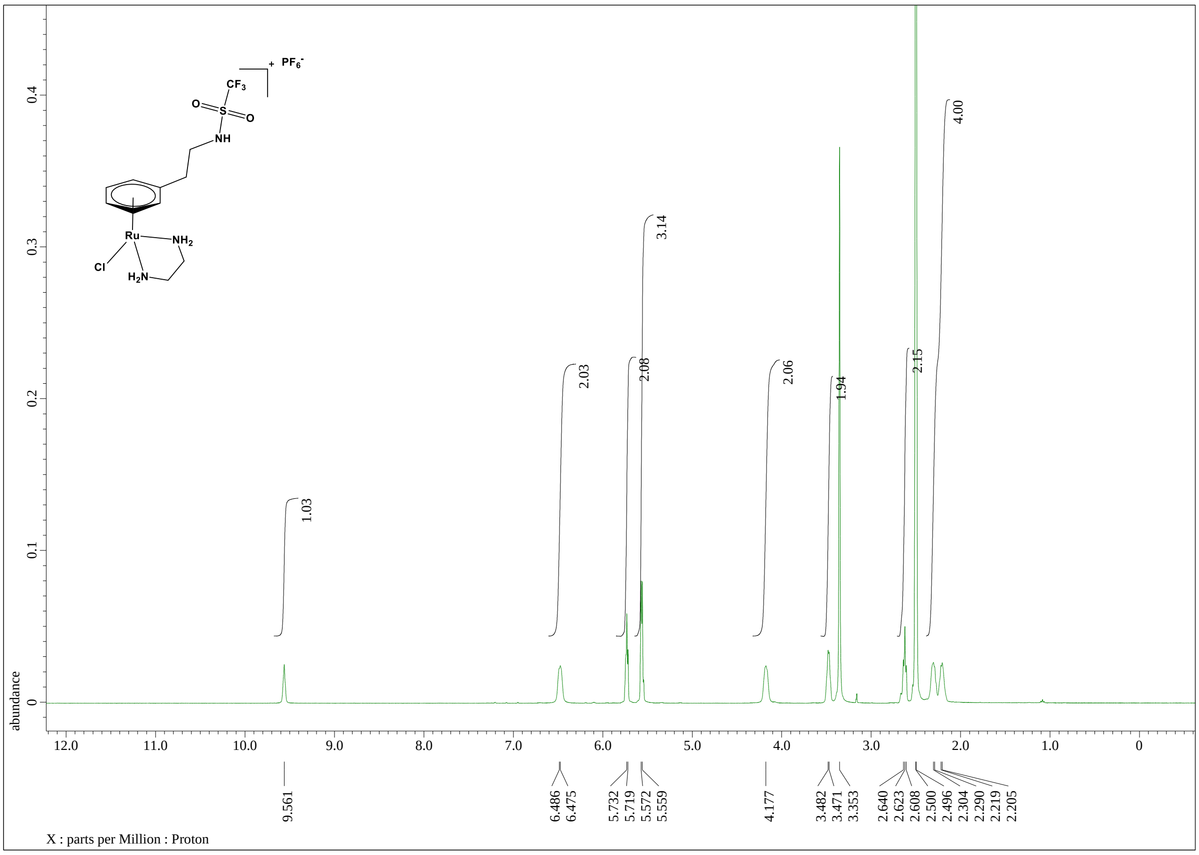

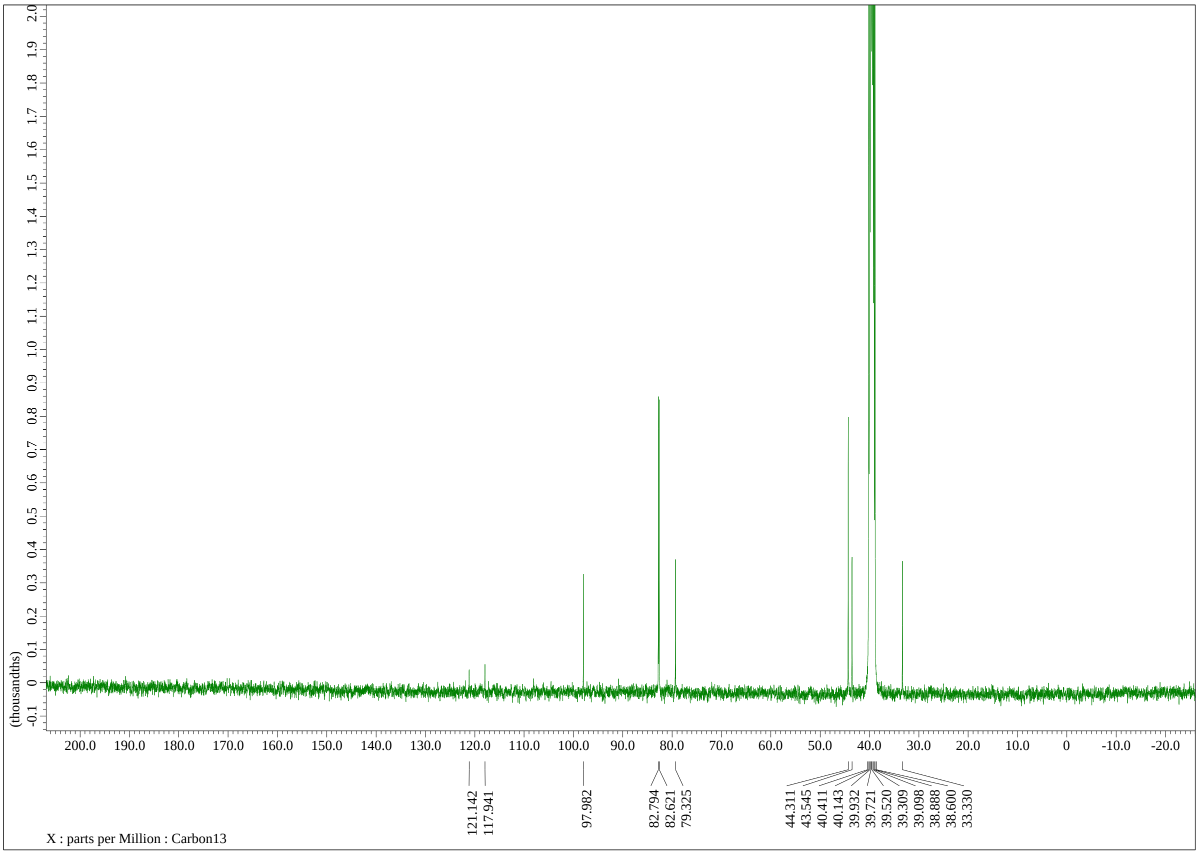

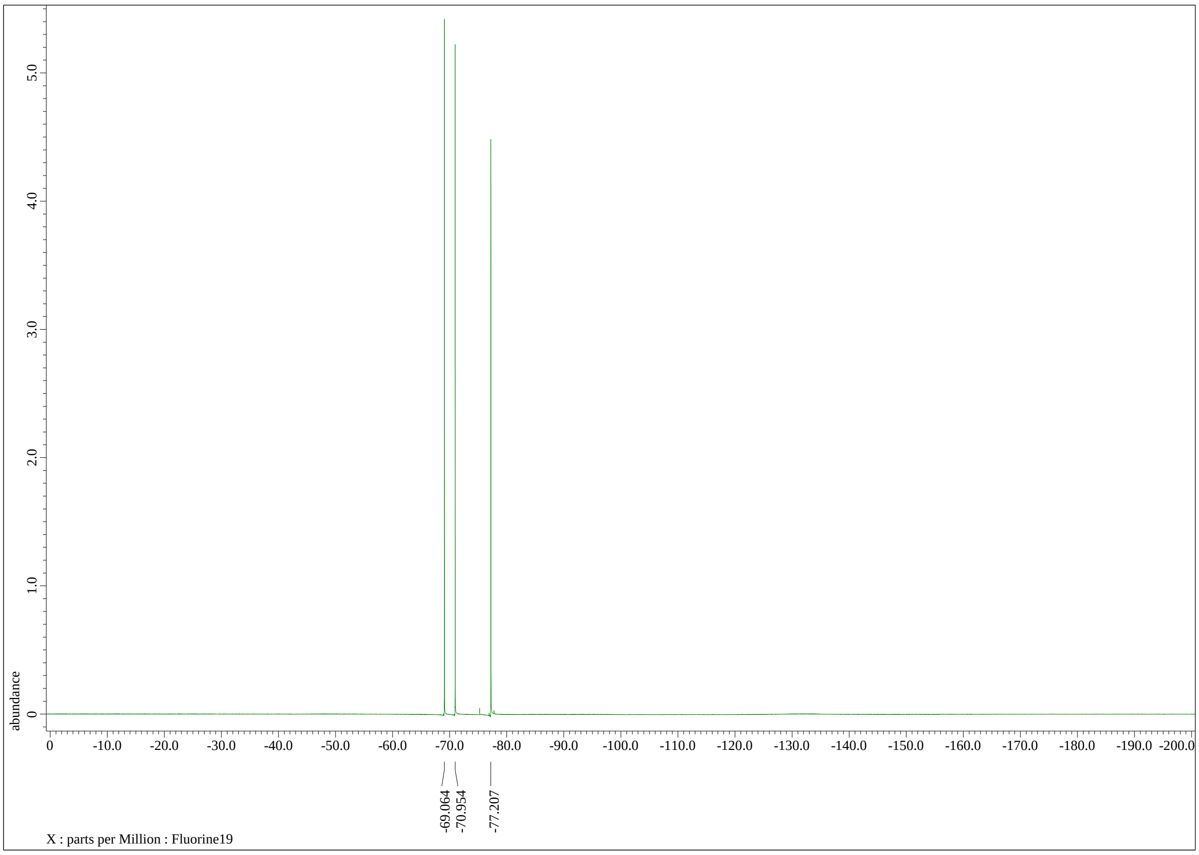
abundance
0
1.0
2.0
3.0
4.0
5.0
0 -10.0 -20.0 -30.0 -40.0 -50.0 -60.0 -70.0 -80.0 -90.0 -100.0 -110.0 -120.0 -130.0 -140.0 -150.0 -160.0 -170.0 -180.0 -190.0 -200.0
-69.064
-70.954
-77.207
X : parts per Million : Fluorine19

Figure S7: ^{1}H (top), ^{13}C{^{1}H} and ^{19}F (following page) NMR spectra (DMSO-d_6) of **4**.

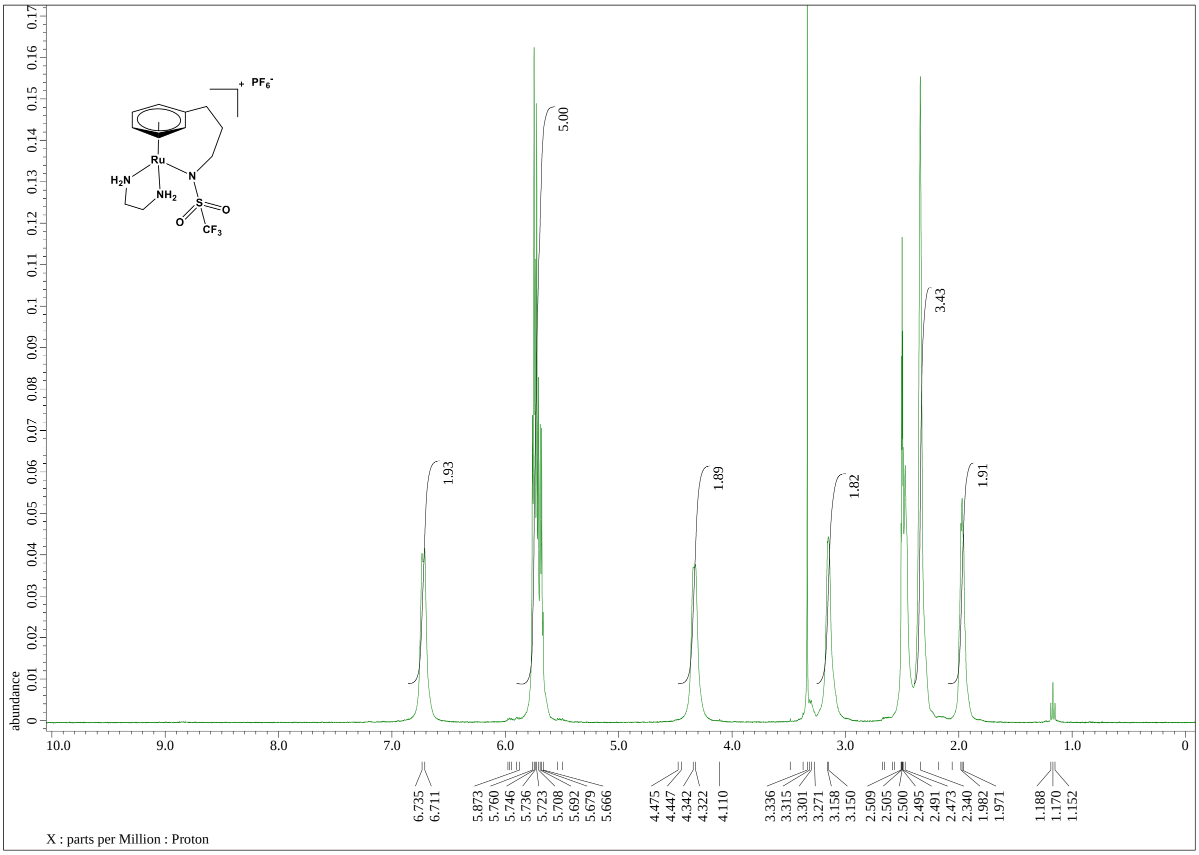

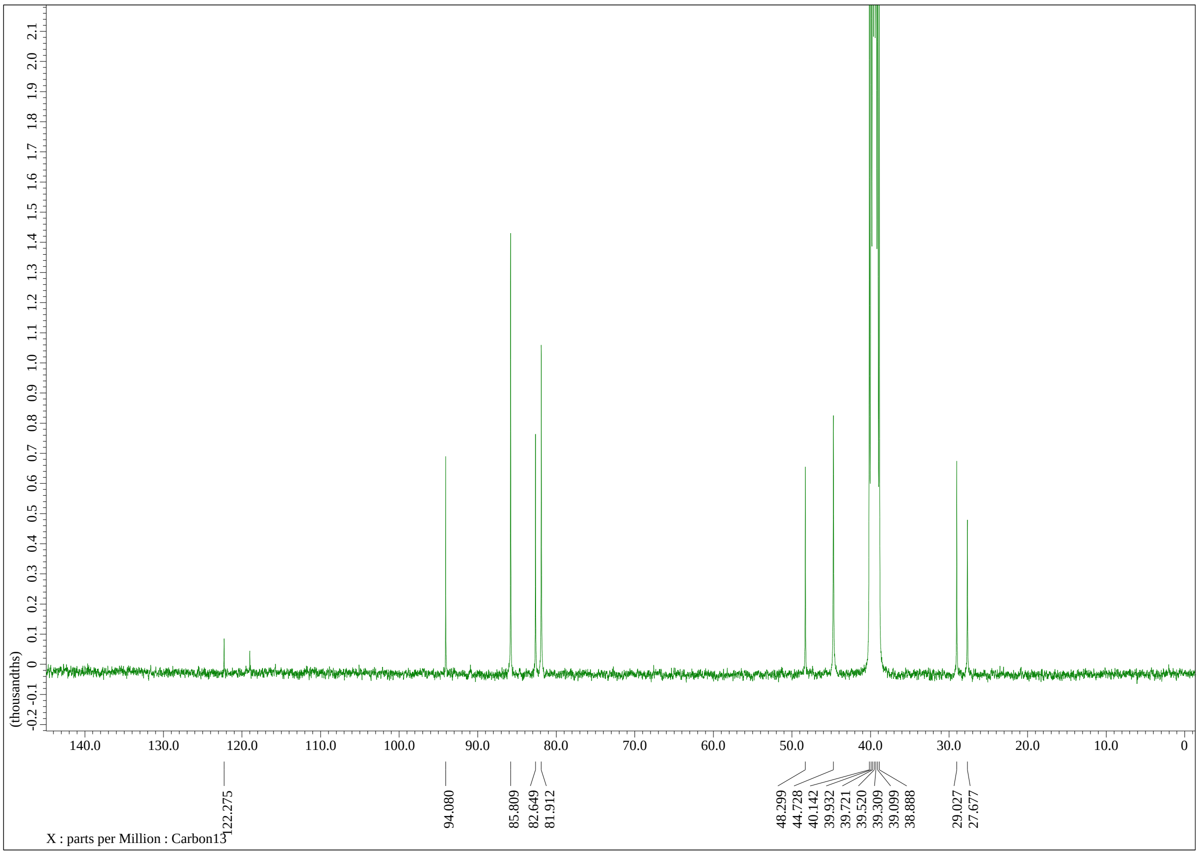

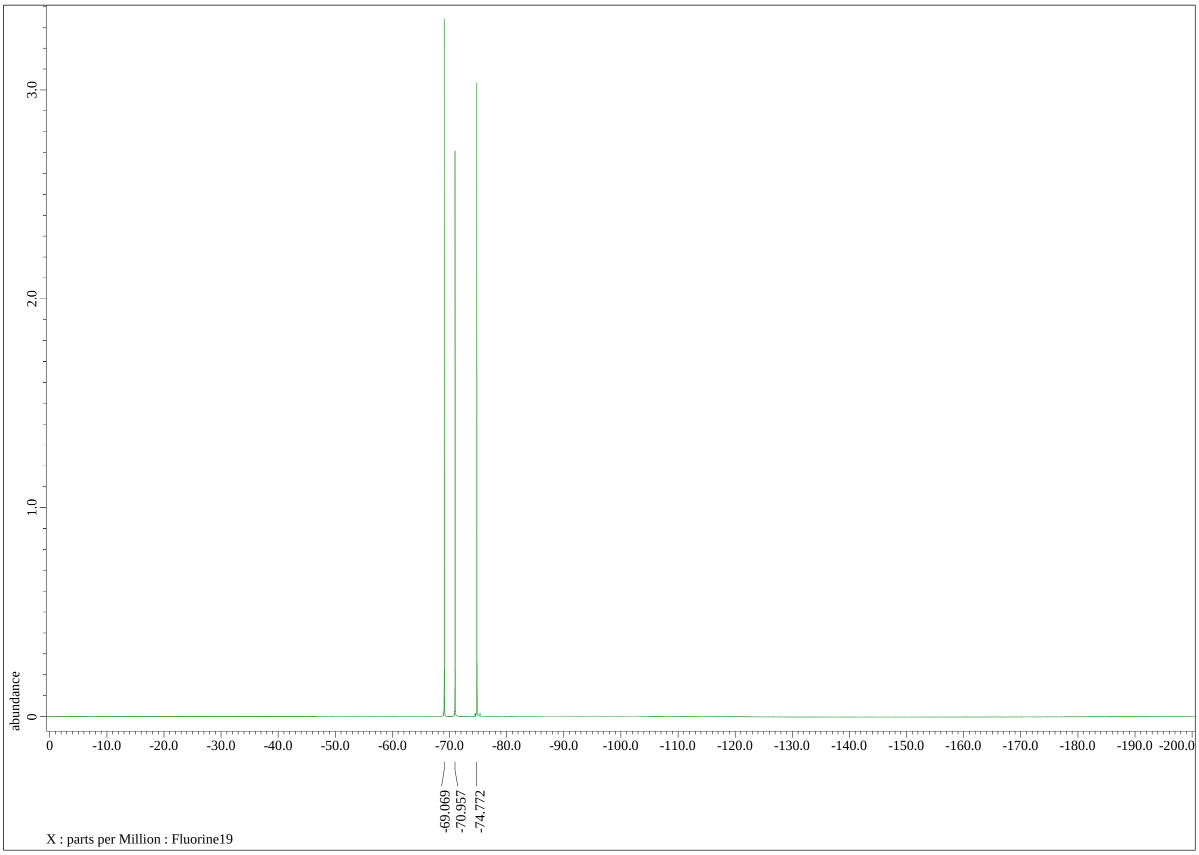

abundance
3.0
2.0
1.0
0
0 -10.0 -20.0 -30.0 -40.0 -50.0 -60.0 -70.0 -80.0 -90.0 -100.0 -110.0 -120.0 -130.0 -140.0 -150.0 -160.0 -170.0 -180.0 -190.0 -200.0
-69.069
-70.957
-74.772
X : parts per Million : Fluorine19

Figure S8: ^{1}H (top), ^{13}C{^{1}H} and ^{19}F (following page) NMR spectra (DMSO-*d*$_6$) of **5**.

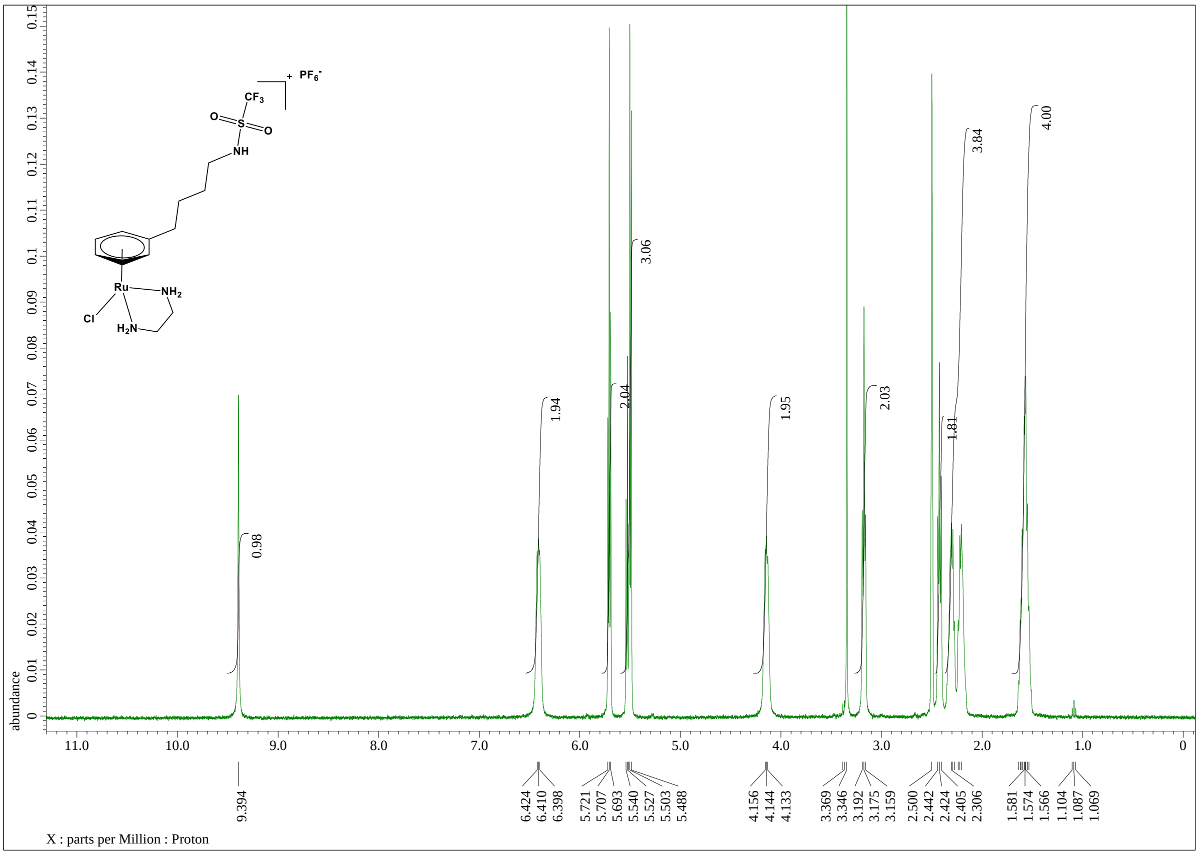

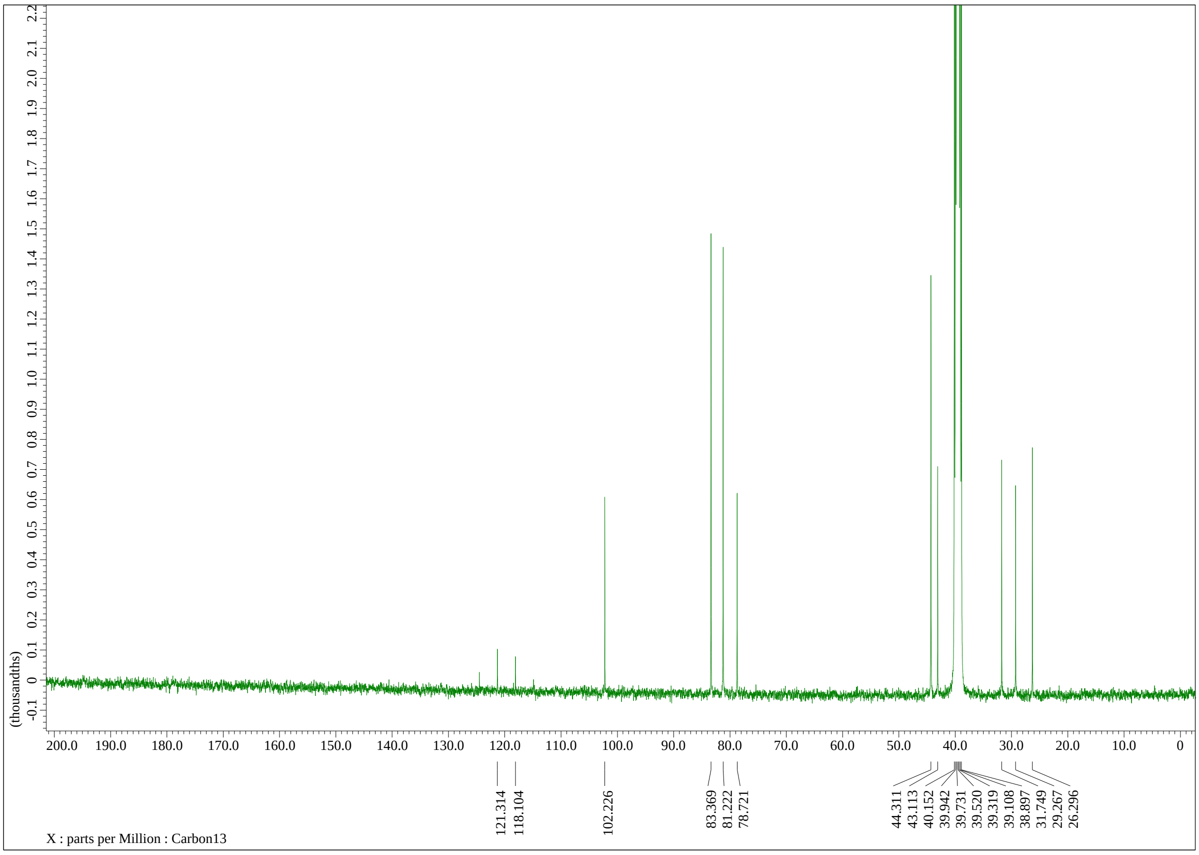

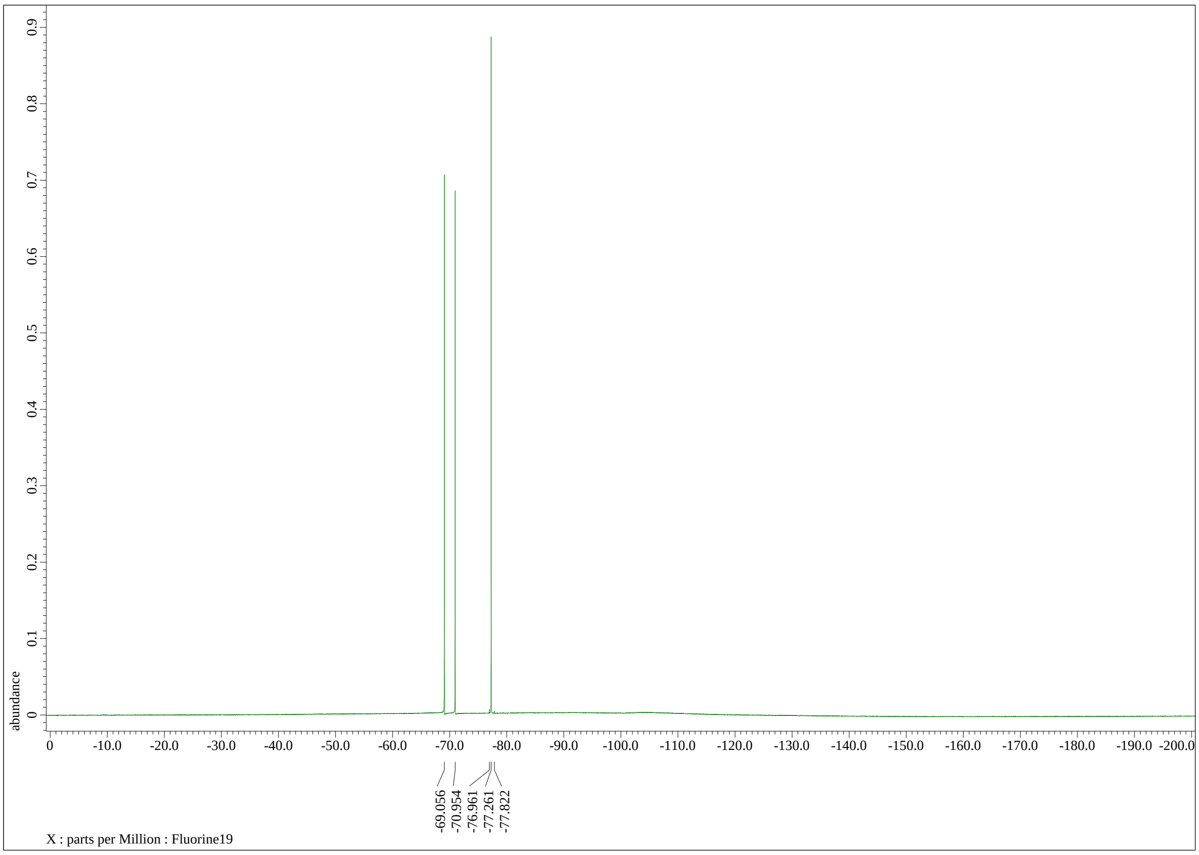
abundance
0.9
0.8
0.7
0.6
0.5
0.4
0.3
0.2
0.1
0
0
-10.0
-20.0
-30.0
-40.0
-50.0
-60.0
-70.0
-80.0
-90.0
-100.0
-110.0
-120.0
-130.0
-140.0
-150.0
-160.0
-170.0
-180.0
-190.0
-200.0
-69.056
-70.954
-76.961
-77.261
-77.822
X : parts per Million : Fluorine19

Figure S9: ^{1}H (top), $^{13}C\{^{1}H\}$ and ^{19}F (following page) NMR spectra (DMSO-d_6) of **6**.

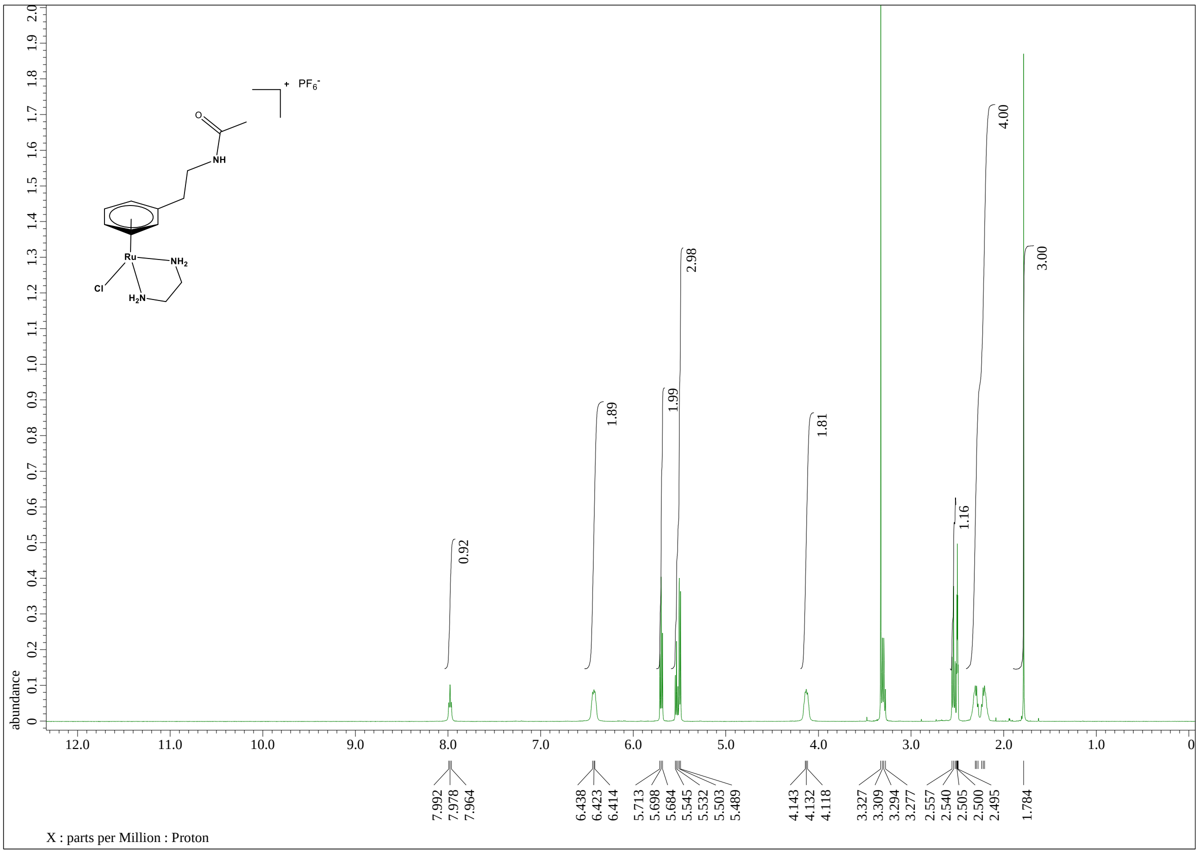

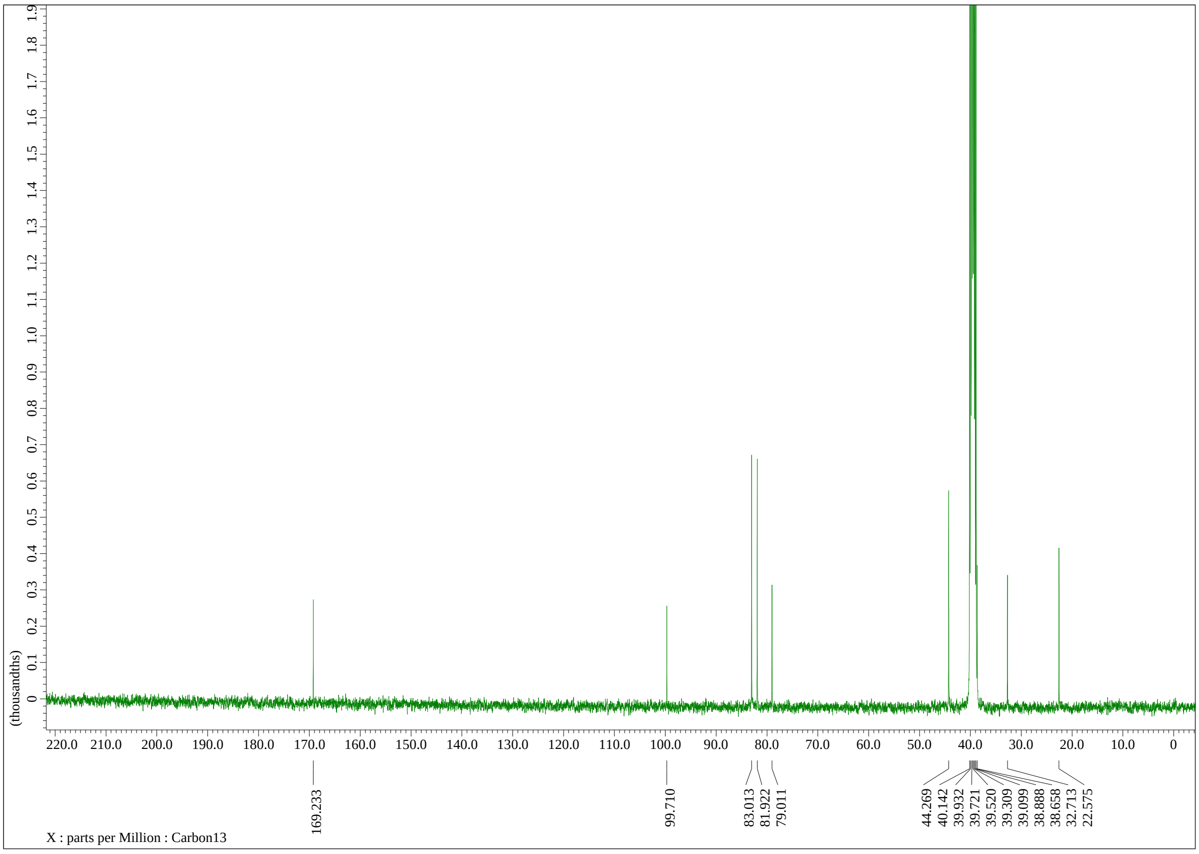

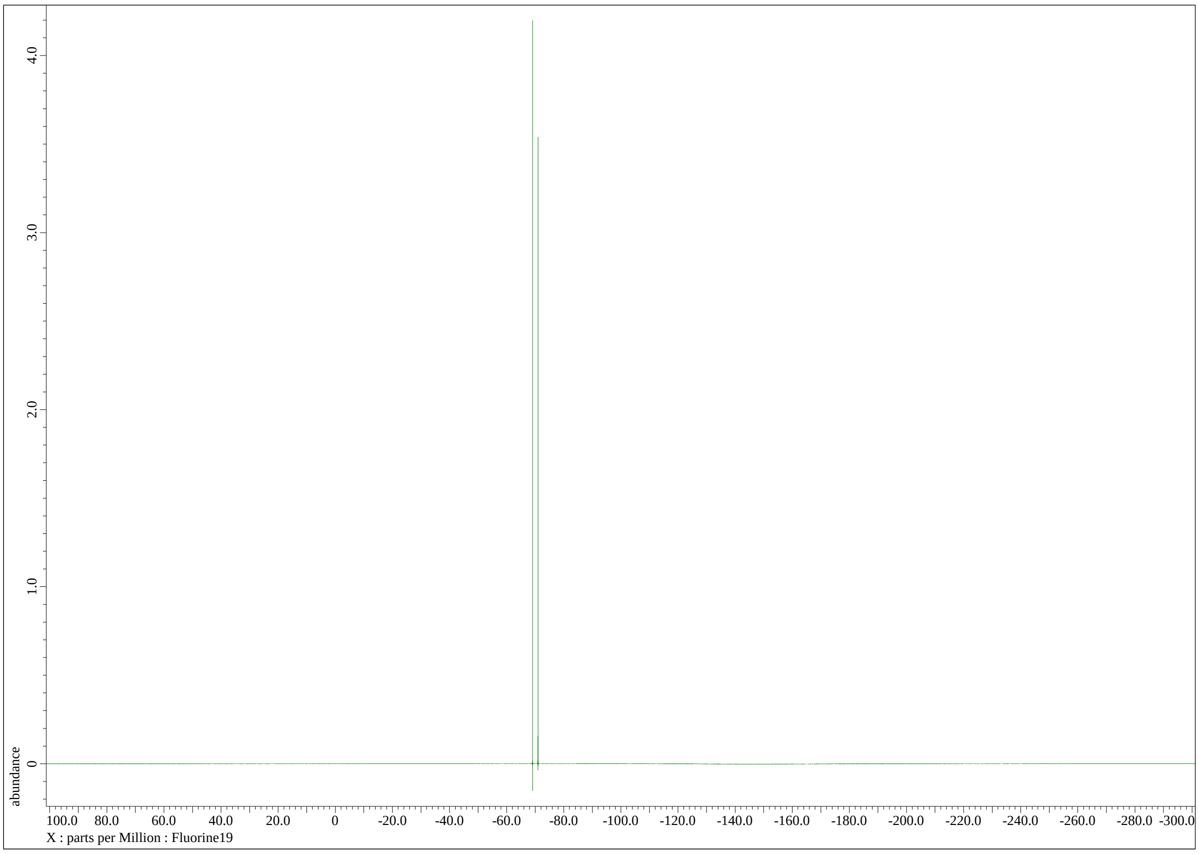

abundance
4.0
3.0
2.0
1.0
0
100.0 80.0 60.0 40.0 20.0 0 -20.0 -40.0 -60.0 -80.0 -100.0 -120.0 -140.0 -160.0 -180.0 -200.0 -220.0 -240.0 -260.0 -280.0 -300.0
X : parts per Million : Fluorine19

Figure S10: ^{1}H (top), $^{13}C\{^{1}H\}$ and ^{19}F (following page) NMR spectra (D_2O) of **[Ru(η⁶-$C_6H_5CH_2CH_2NHTr$)(C_2O_4)(H_2O)].**

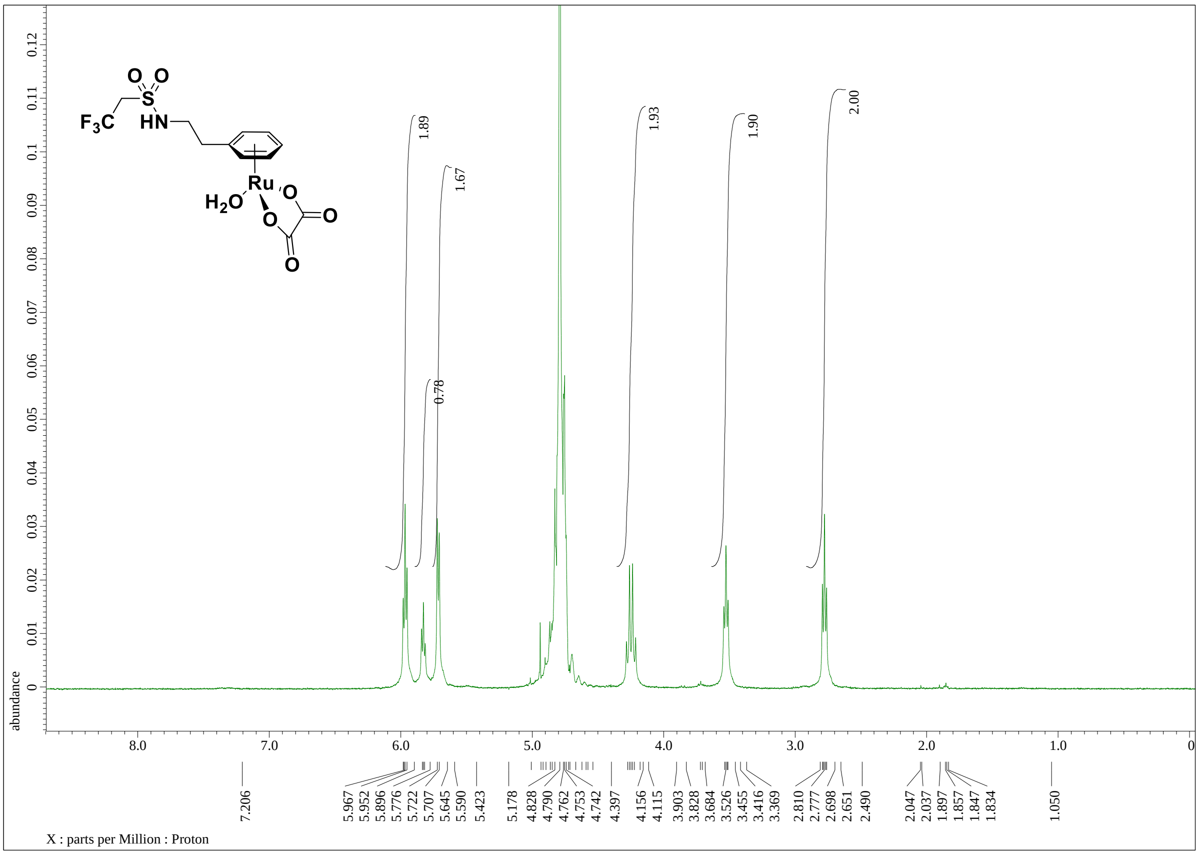

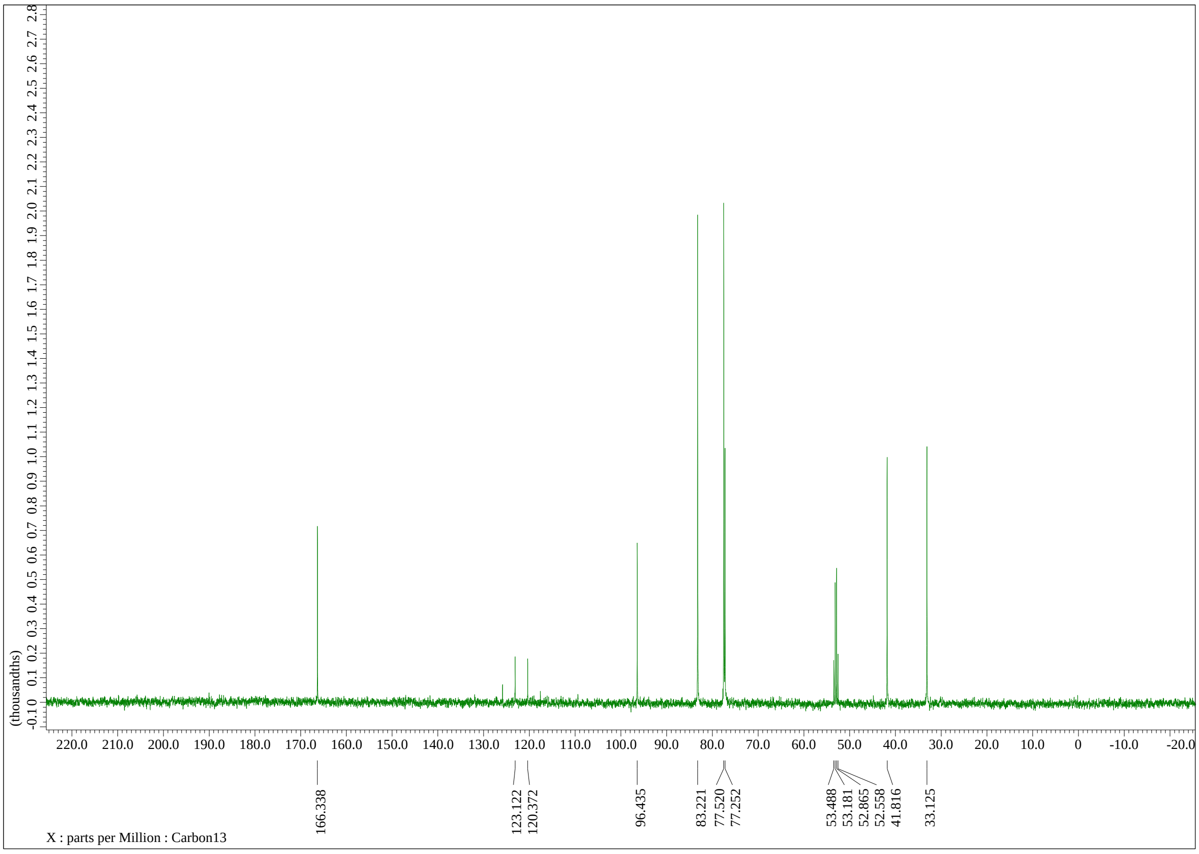

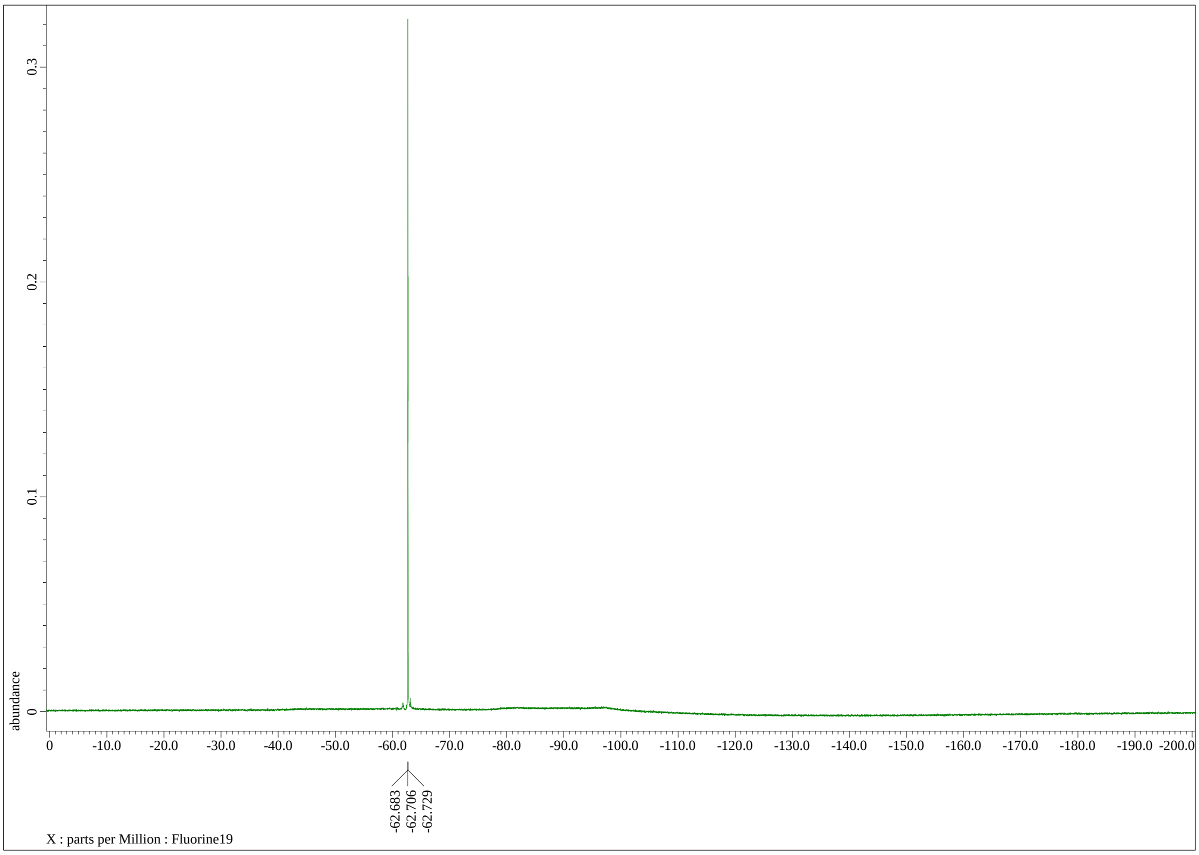
0.3
0.2
0.1
0
abundance
0
-10.0
-20.0
-30.0
-40.0
-50.0
-60.0
-70.0
-80.0
-90.0
-100.0
-110.0
-120.0
-130.0
-140.0
-150.0
-160.0
-170.0
-180.0
-190.0
-200.0
-62.683
-62.706
-62.729
X : parts per Million : Fluorine19

Figure S11: ^{1}H NMR (D_2O, 295 K, 2.46 mM complex, 0.1 M NaCl) spectra of **6** at pD = 4.15 (top), 5.31 (middle) and 7.07 (bottom) – recorded after the solutions were stood for 18 h at 295 K.

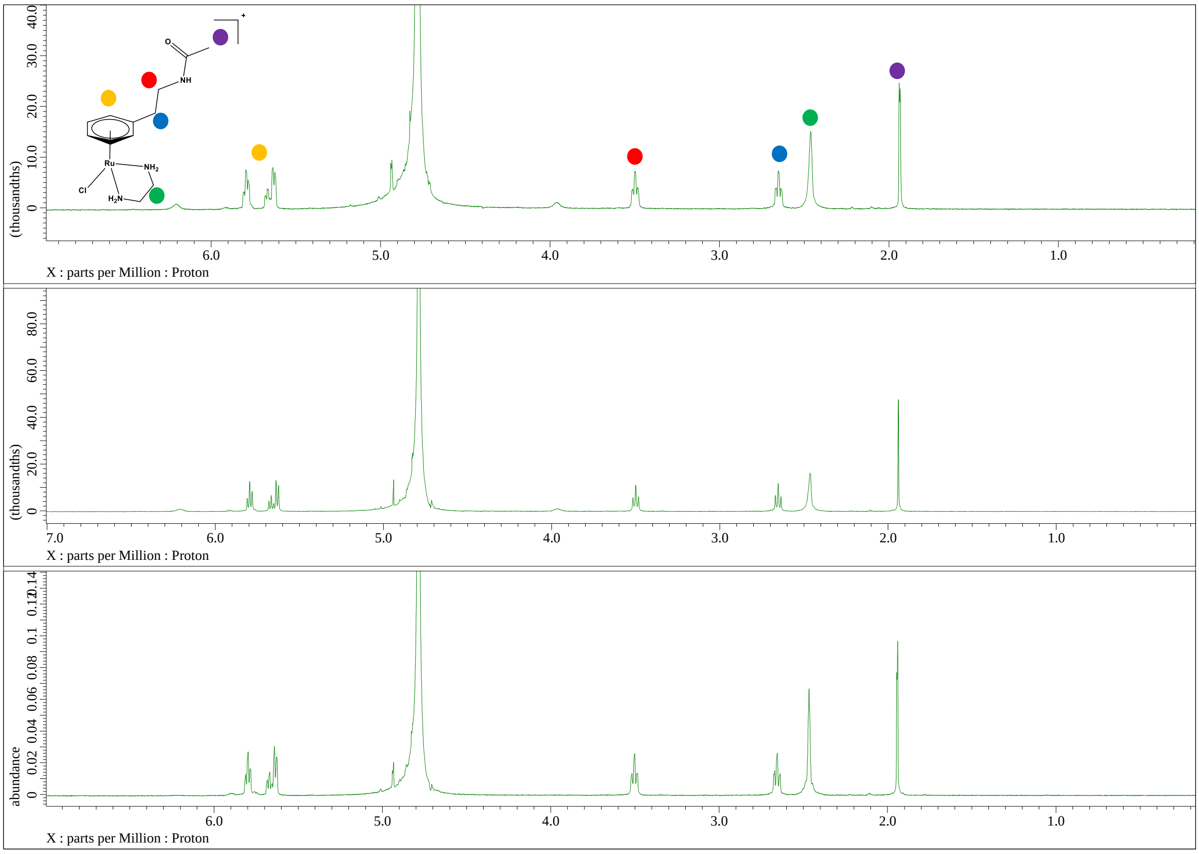

Figure S12: ^{1}H NMR (D_2O, 295 K, 2.46 mM complex, 0.1 M NaCl) spectra of **1** at pD = 5.98 (top) and 9.04 (bottom) – conditions where the open- and chelate-forms of the complexes dominate respectively. Selected peaks are labelled (coloured circles) to illustrate chemical shift differences between the open and chelate-form of the complex. Arene loss can be observed in the bottom spectrum – labelled with a black circle.

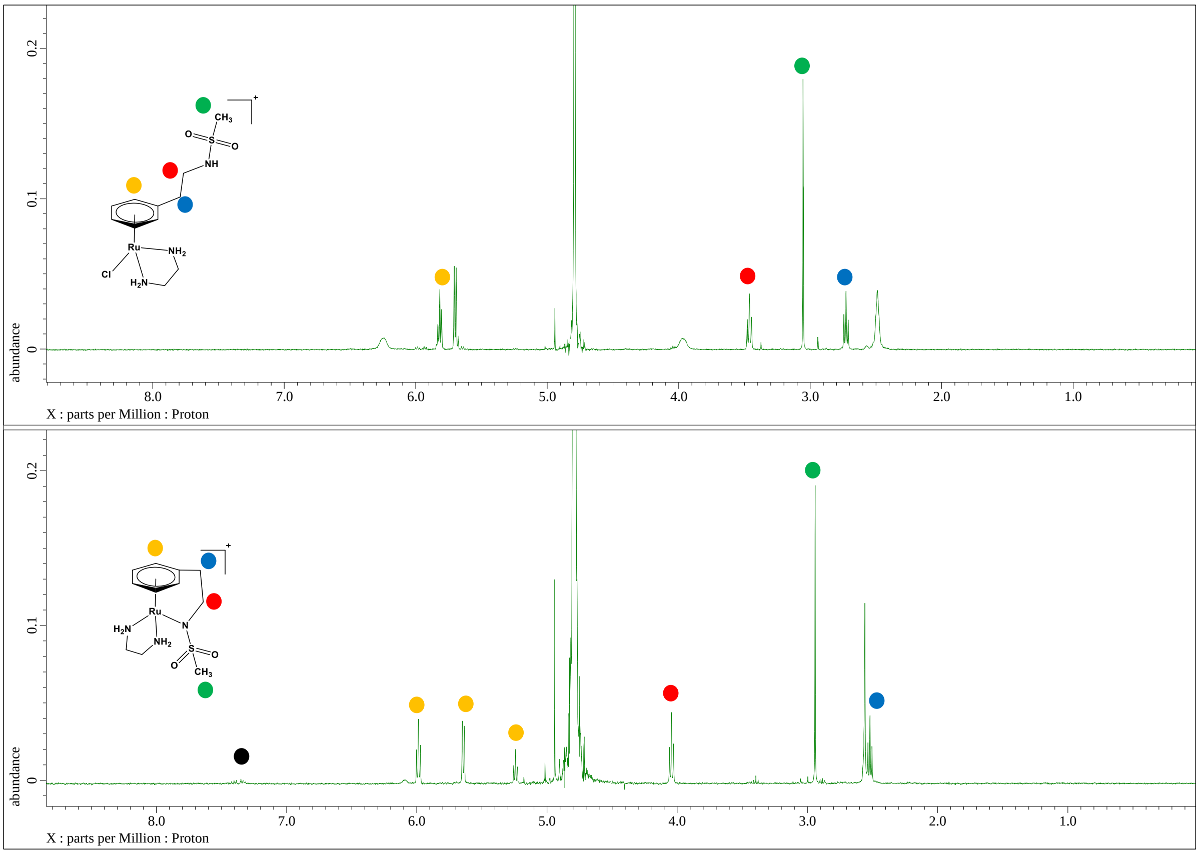

Figure S13: ^{1}H NMR (D_2O, 295 K, 2.46 mM complex, 0.1 M NaCl) spectra of **2** at pD = 4.16 (top) and 7.98 (bottom) – conditions where the open- and chelate-forms of the complexes dominate respectively. Selected peaks are labelled (coloured circles) to illustrate chemical shift differences between the open and chelate-form of the complex. Arene loss can be observed in the bottom spectrum – labelled with a black circle.

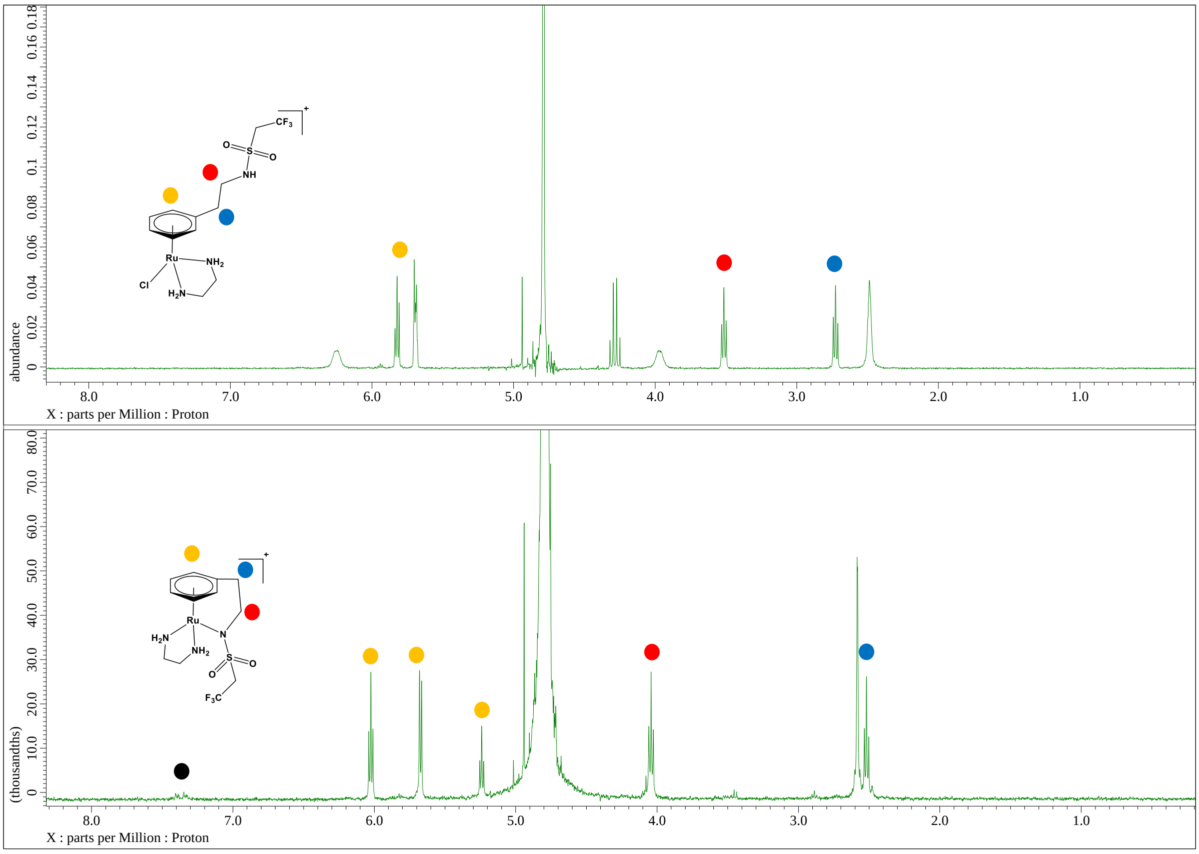

Figure S14: ^1H NMR (D_2O, 295 K, 2.46 mM complex, 0.1 M NaCl) spectra of **3** at pD = 3.24 (top) and 7.46 (bottom) – conditions where the open- and chelate-forms of the complexes dominate respectively. Selected peaks are labelled (coloured circles) to illustrate chemical shift differences between the open and chelate-form of the complex. Arene loss can be observed in the bottom spectrum – labelled with a black circle.

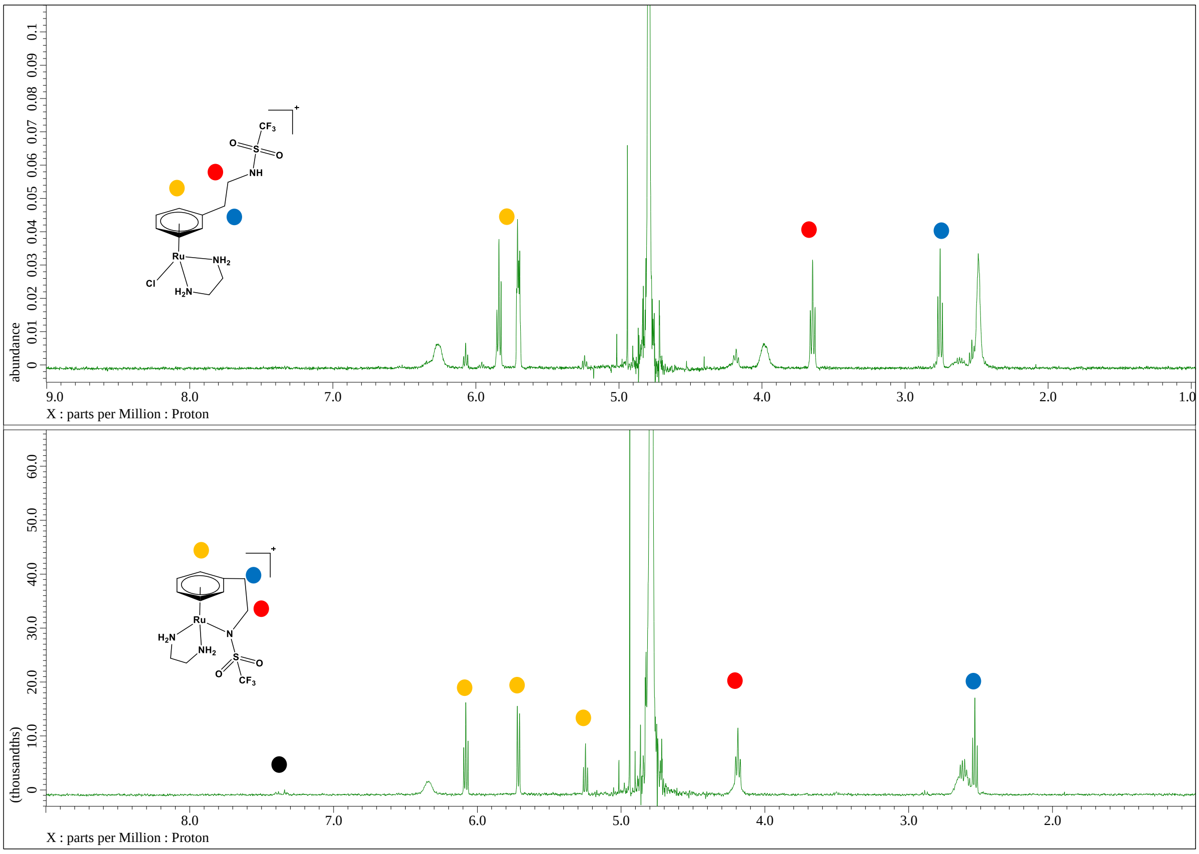

Figure S15: ^{1}H NMR (D_2O, 295 K, 2.46 mM complex, 0.1 M NaCl) spectra of **4** at pD = 1.55 (top) and 7.40 (bottom) – conditions where the open- and chelate-forms of the complexes dominate respectively. Selected peaks are labelled (coloured circles) to illustrate chemical shift differences between the open and chelate-form of the complex.

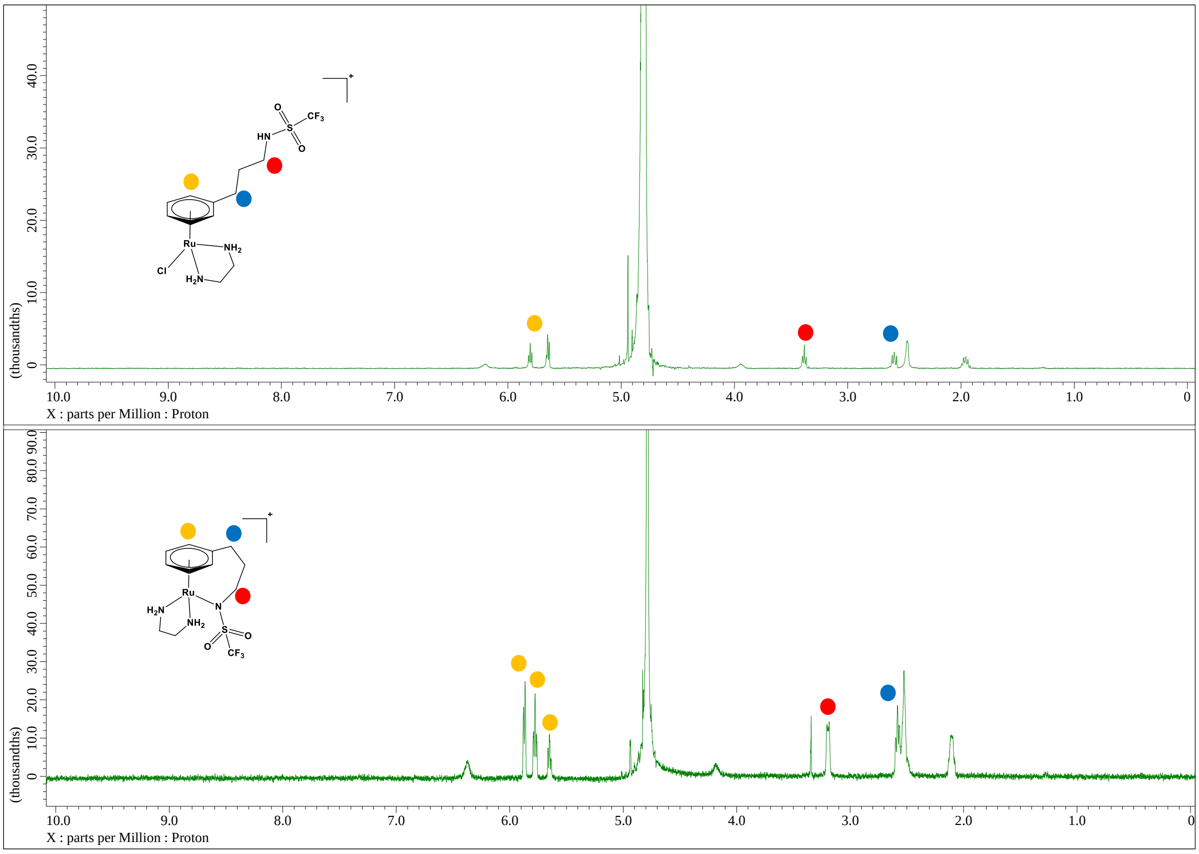

Figure S16: ^{1}H NMR (D_2O, 295 K, 2.46 mM complex, 0.1 M NaCl) spectra of **4** with 1 eq 5′-GMP at pD = 7.5 at t = 0 h (top) and t = 144 h (bottom) following incubation at 310 K. The spectra highlight the lack of reactivity between **4** and 5′-GMP under these pH conditions.

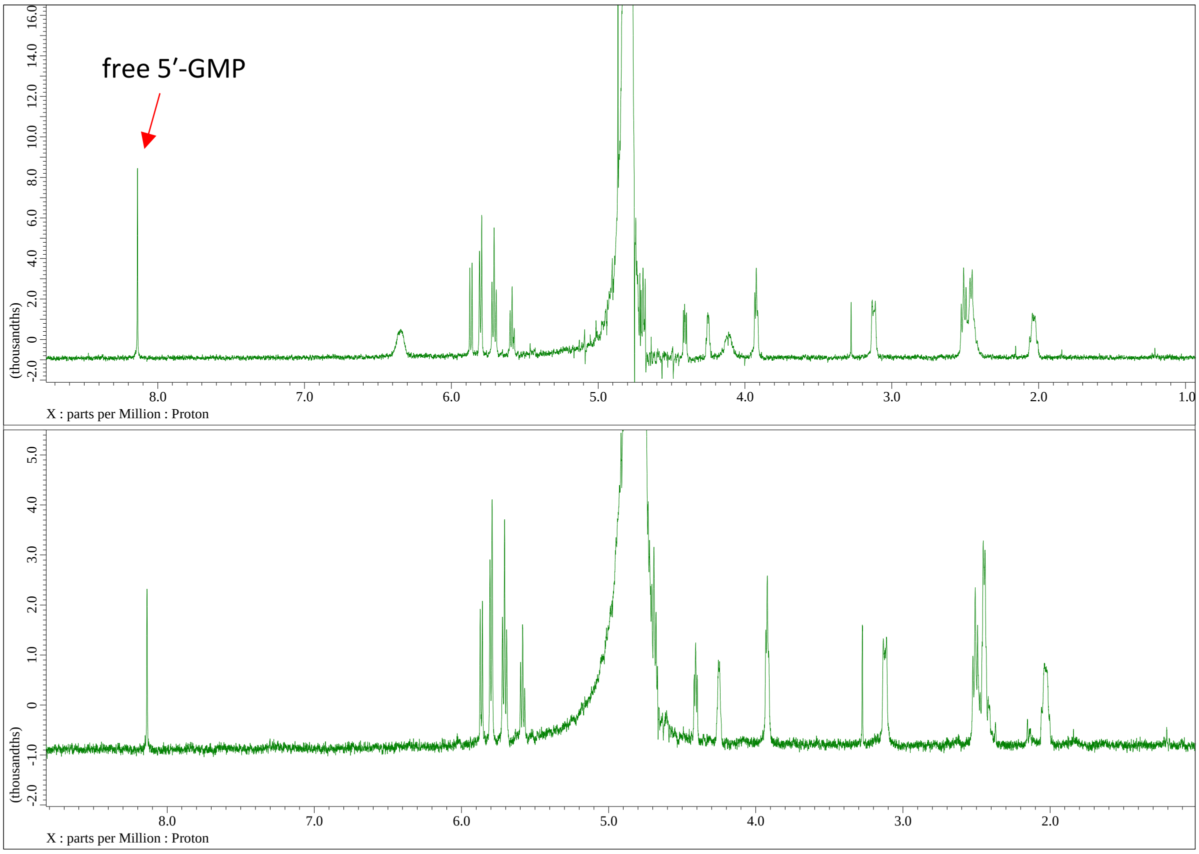

Figure S17: ^{1}H NMR (D_2O, 295 K, 2.46 mM complex, 0.1 M NaCl) spectra of **4** with 1 eq 5′-GMP at pD = 6.5 at t = 0 h (top) and t = 144 h (bottom) following incubation at 310 K. The spectra highlight the slightly increased reactivity between **4** and 5′-GMP under these pH conditions versus pD = 7.5. Due to the low intensity of the signal for the 5′-GMP H8 proton associated with the Ru-5′-GMP adduct this experiment was repeated in quadruplicate. The percentage of 5′-GMP coordinated to ruthenium after 144 h incubation was calculated to be 16 ± 1.6 % - this value being the average of the four experiments.

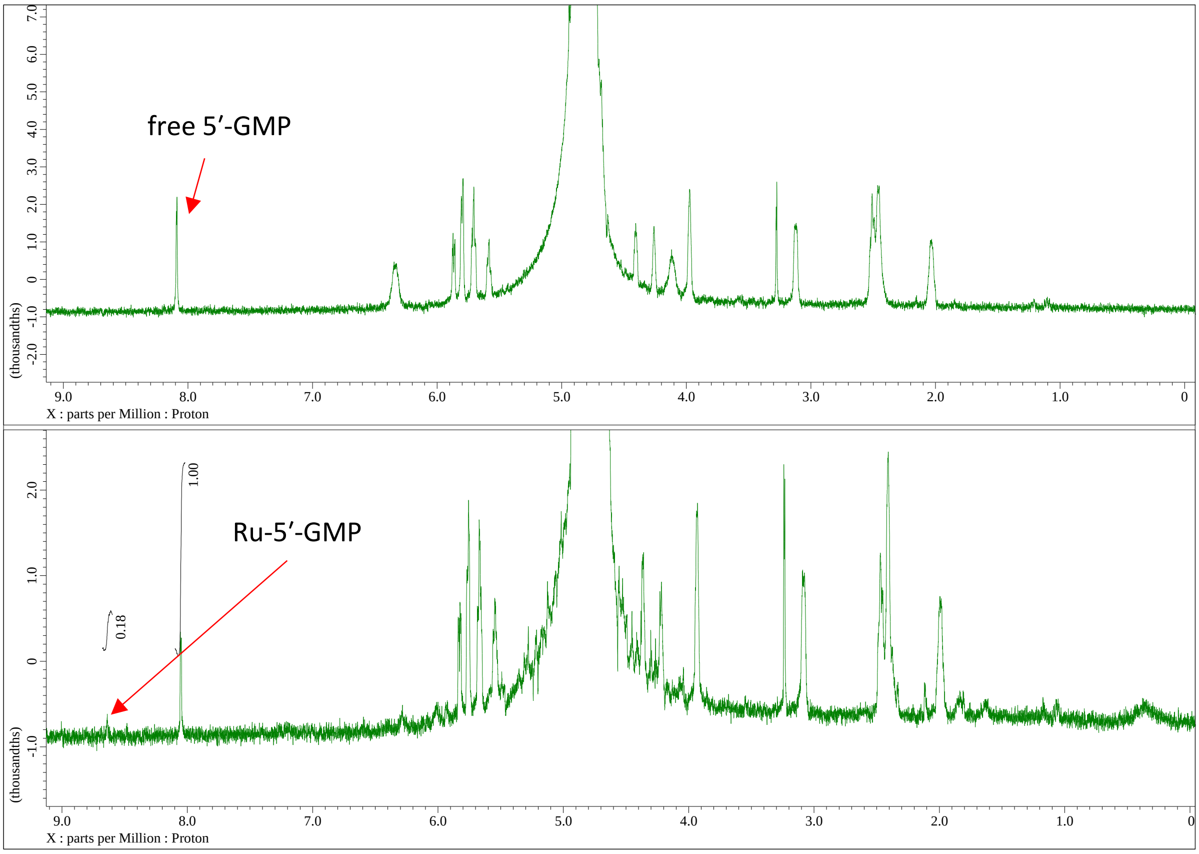

Figure S18: ^{1}H NMR (D_2O, 295 K, 2.46 mM complex, 0.1 M NaCl) spectra of **6** with 1 eq 5′-GMP at pD = 7.5 (top) and pD = 6.5 (bottom) at t = 144 h following incubation at 310 K. The spectra highlight the reactivity of **6** and 5′-GMP under both pH conditions.

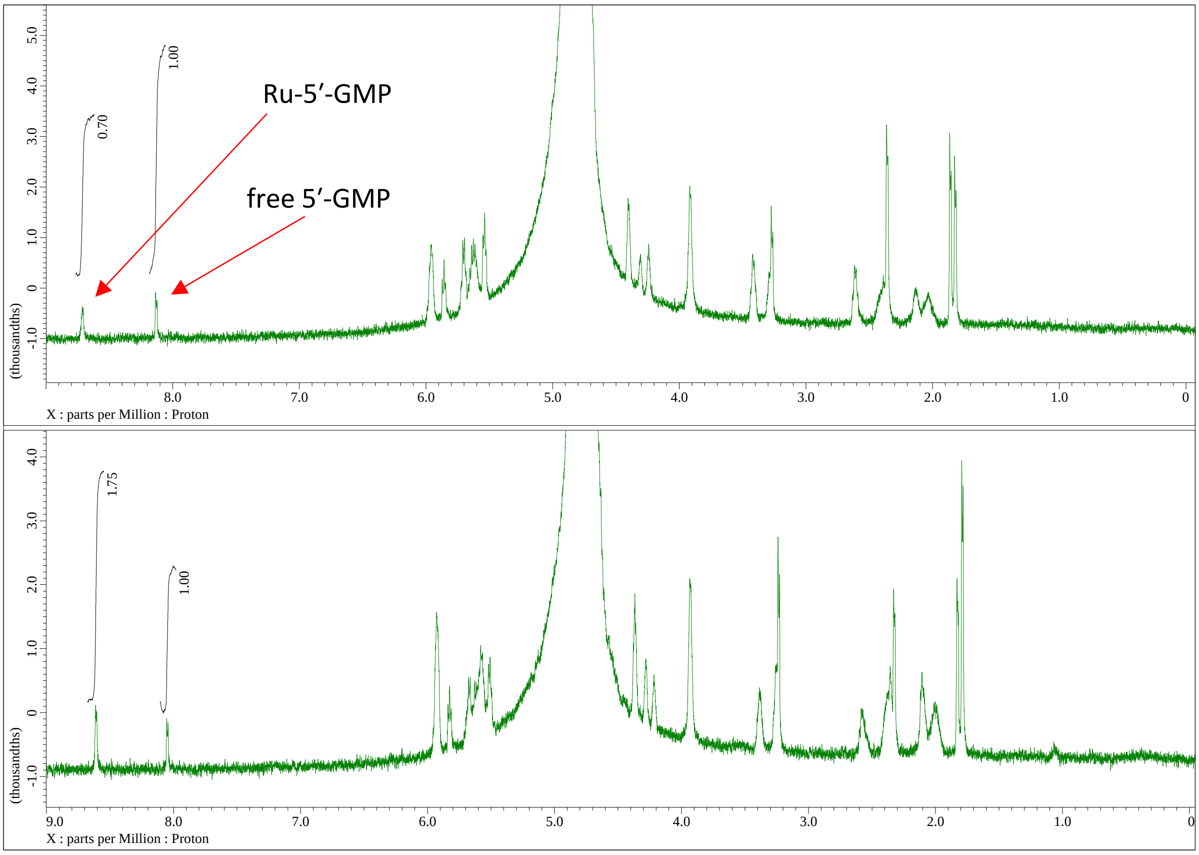

Figure S19: ^{1}H NMR (D_2O, 295 K, 2.46 mM complex, 0.1 M NaCl) spectra of **4** with 1 eq L-histidine at pD = 7.5 at t = 0 h (top) and t = 144 h (bottom) following incubation at 310 K. The spectra highlight the lack of reactivity between **4** and L-histidine under these pH conditions. The changing ratio between L-histidine imidazole proton resonances over the incubation is most likely explained by hydrogen-deuterium exchange at the C2 proton [21].

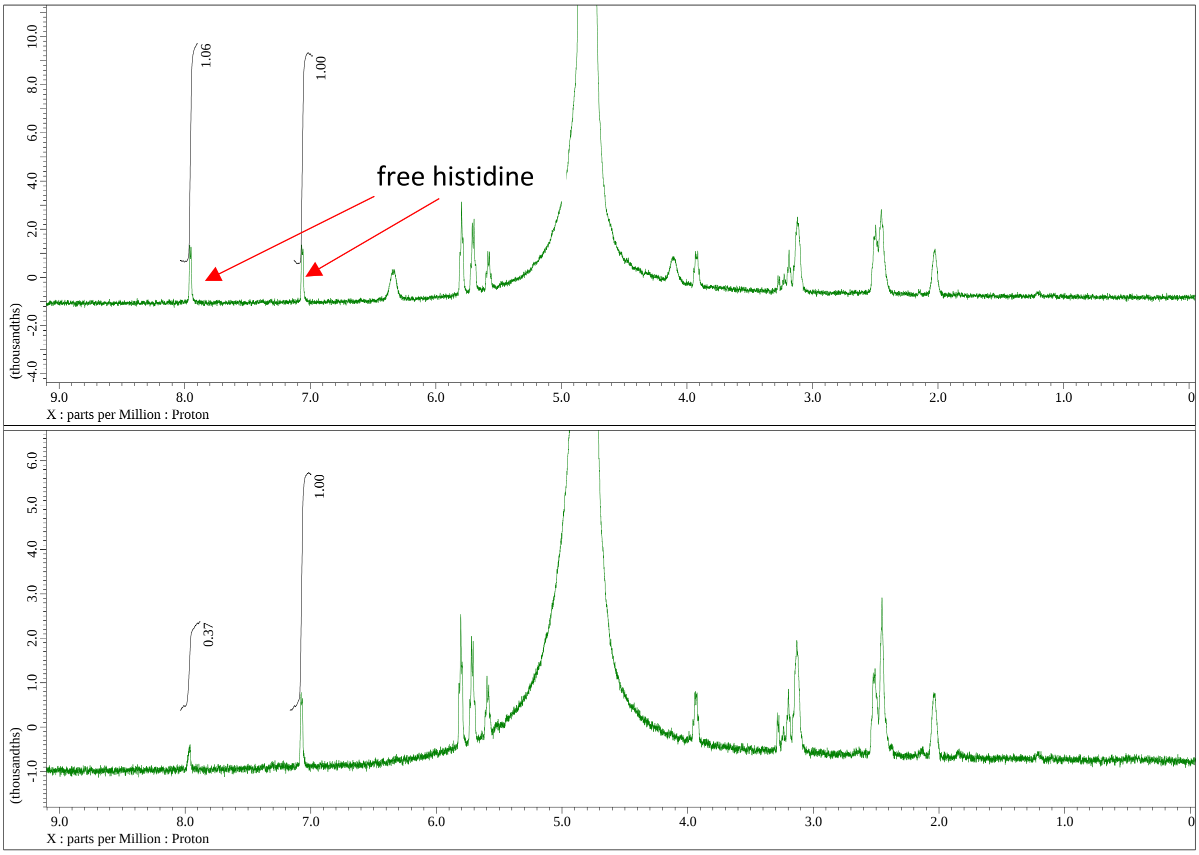

Figure S20: ^{1}H NMR (D_2O, 295 K, 2.46 mM complex, 0.1 M NaCl) spectra of **4** with 1 eq L-histidine at pD = 6.5 at t = 0 h (top) and t = 144 h (bottom) following incubation at 310 K. The spectra highlight the lack of reactivity between **4** and L-histidine under these pH conditions. The changing ratio between L-histidine imidazole proton resonances over the incubation is most likely explained by hydrogen-deuterium exchange at the C2 proton [21].

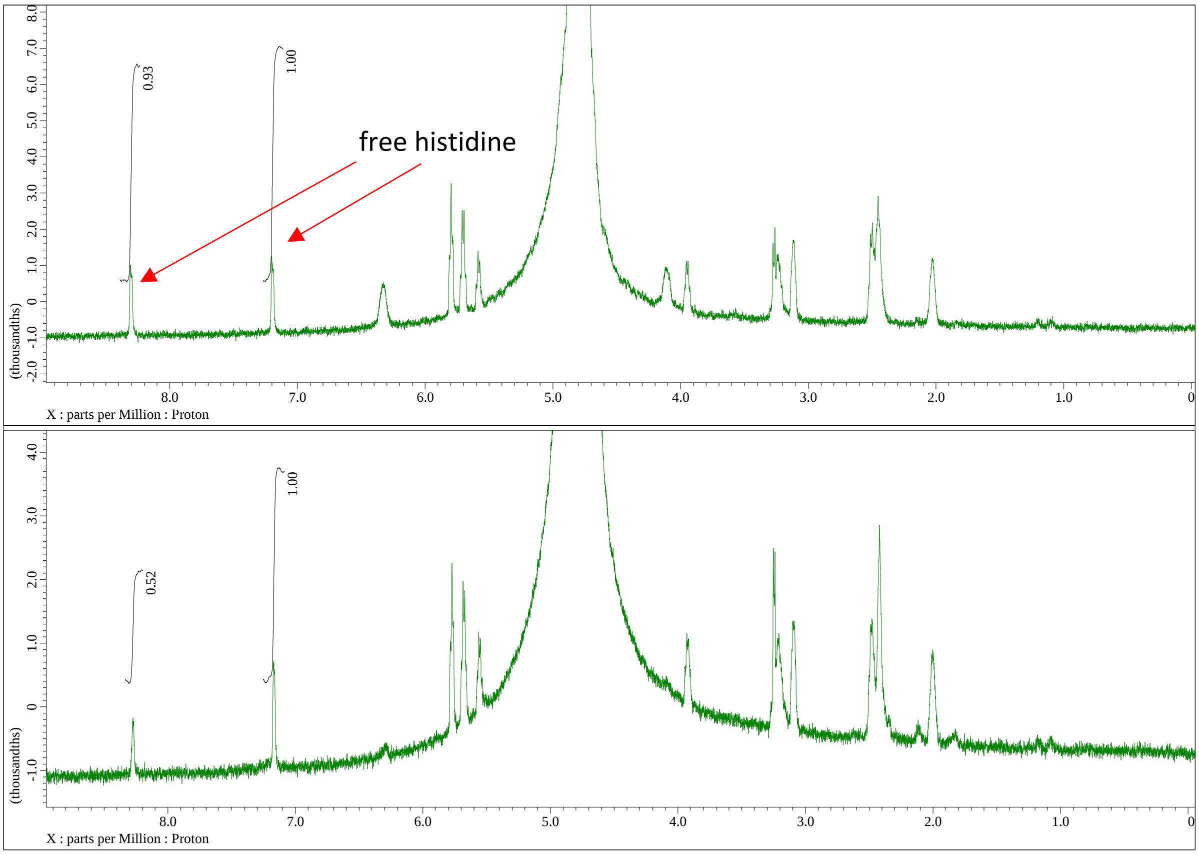

Figure S21: ^{1}H NMR (D_2O, 295 K, 2.46 mM complex, 0.1 M NaCl) spectra of **6** with 1 eq L-histidine at pD = 7.5 (top) and pD = 6.5 (bottom) at t = 144 h following incubation at 310 K. The spectra highlight the reactivity of **6** and L-histidine under both pH conditions, but predominantly at pD 7.5 due to increased imidazole protonation at pD 6.5.

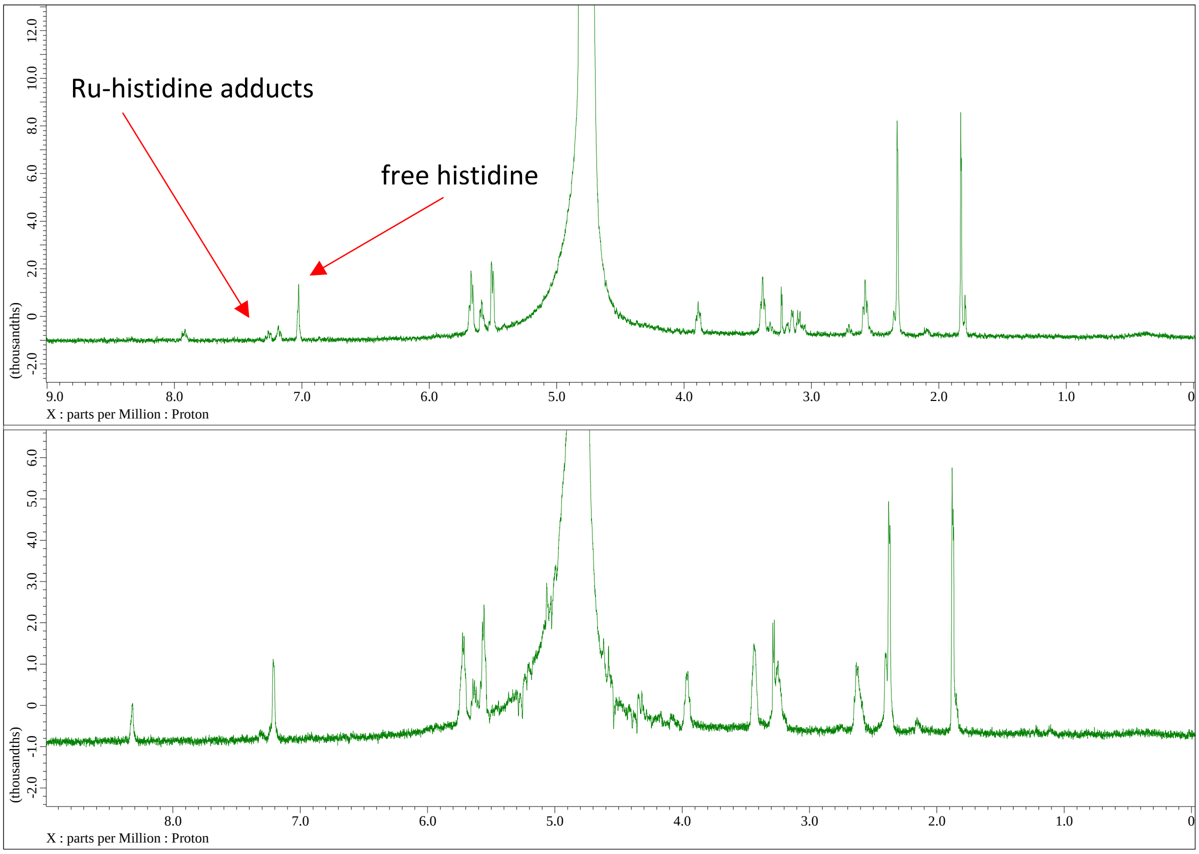

Figure S22: ^{1}H NMR (D_2O, 295 K, 2.46 mM complex, 200 mM phosphate buffer, 0.1 M NaCl) spectra of **4** with 0.5 mM bovine serum albumin t = 0 h (top) and t = 72 h (bottom) following incubation at 310 K. The spectra highlight the lack of reactivity of **4** towards bovine serum albumin under these conditions.

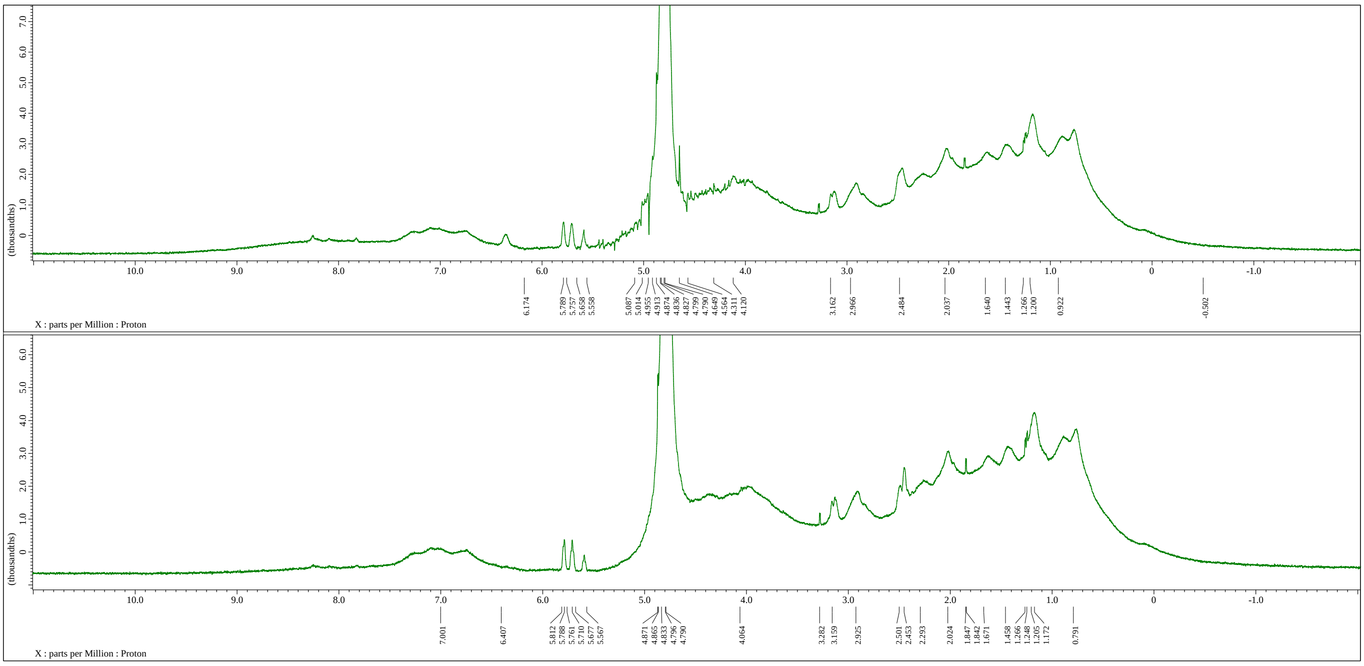

References

21. Bradbury, J.H.; Chapman, B.E.; Pellegrino, F.A.; Hydrogen-Deuterium Exchange Kinetics of the C-2 Protons of Imidazole and Histidine Compounds. *J. Am. Chem. Soc*. **1973**, 95, 6139–6140.
